# Mechanism study of tyrosine phosphatase shp-1 in inhibiting hepatocellular carcinoma progression by regulating the SHP2/GM-CSF pathway in TAMs

**DOI:** 10.1038/s41598-024-59725-w

**Published:** 2024-04-21

**Authors:** Qiang Wei, ShuBin Luo, Gang He

**Affiliations:** 1 Bethune International Peace Hospital of PLA Hepatobiliary Surgery, Shijiazhuang, 050082 China; 2 Department of General Surgery (Section 1), The First People′s Hospital of Jinghong, Jinghong, 666100 China; 3 Bethune International Peace Hospital of PLA ICU, No. 398 West Zhongshan Road, Shijiazhuang, 050082 China

**Keywords:** SHP-1, SHP2, GM-CSF, HOXA10, Hepatocellular carcinoma, Cancer microenvironment, Tumour-suppressor proteins, Cell migration

## Abstract

Hepatocellular carcinoma (HCC) is one of the most common malignant tumors worldwide. Macrophage-mediated innate immune responses play a crucial role in tumor development. This study revealed the mechanism of SHP-1 in regulating HCC progression. SHP-1 inhibits tumour development in vivo. Increasing SHP-1 expression in macrophages promotes the expression of p-SHP-1, SHP2, and p-SHP-2. In macrophages GM-CSF recruits SHP-2 to the GM-CSF receptor GM-CSFR induces p-SHP-2 dephosphorylation. GM-CSF recruits p-SHP-2 for dephosphorylation by up-regulating HoxA10HOXA10 activates the transcription of TGFβ2 by interacting with tandem cis-elements in the promoter thereby regulating the proliferation and migration of liver cancer cells. GM-CSF inhibits SHP-1 regulation of p-SHP-1, SHP2, and p-SHP-2 in macrophages. Detailed studies have shown that SHP-1 regulates SHP2 expression, and SHP-1 and SHP2 are involved in macrophage M2 polarisation. SHP-1 inhibits HOXA10 and TGFβ2 which in turn regulates the expression of the migration-associated proteins, MMP2/9, and the migration of hepatocellular carcinoma cells. Overexpression of SHP-1 inhibits macrophage M2 polarisation via the p-STAT3/6 signalling pathway Classical markers arginase-1, CD206, CD163 and regulate the expression of M2 polarisation cytokines IL-4 and IL-10. In addition, hypoxia-induced ROS inhibited SHP-1 regulation by suppressing the expression of p-SHP-1. The combined effect of GM-CSF and ROS significantly increased p-HOXA10/TGFβ2 and macrophage M2 polarisation, and the regulatory effect of ROS was significantly suppressed by GM-CSF knockdown. These findings suggest that increasing the expression of tyrosine phosphatase SHP-1 can inhibit hepatocellular carcinoma progression by modulating the SHP2/GM-CSF pathway in TAM and thus inhibit the progression of hepatocellular carcinoma.

## Introduction

Hepatocellular carcinoma (HCC) is a highly malignant tumor and ranks second in terms of global mortality rates. Despite significant advances in HCC treatment over the past few decades, its effectiveness remains limited. Furthermore, the prognosis for HCC patients is poor due to recurrence and metastasis. Therefore, the search for new treatment strategies and molecular targets is of great importance in improving the survival rate of HCC patients^1,2^.

Recent studies have shown that immunotherapy has significant potential in the field of tumor treatment^3,4^. Macrophages, as important immune cells, play a crucial role in tumor occurrence and development, particularly their innate immune response in tumor suppression^5^. Tumor-associated macrophages (TAMs) are one of the major immune cell components in liver cancer tissues, and they function in angiogenesis, promoting tumor cell proliferation and migration, and suppressing anti-tumor immunity^6^. To reflect the Th1/Th2 immune response, the classical concept suggests that macrophages have two distinct polarization states: M1 (induced by lipopolysaccharide and IFN-γ) and M2 (induced by IL-4 or IL-13). Studies have shown that TAMs primarily exhibit an M2-like phenotype, characterized by immune suppression and favoring tumor progression. Therefore, targeting M2-like TAMs and depleting them within TAMs, or reversing M2-like TAMs into an M1-like phenotype, is a potential immunotherapeutic strategy against tumors^7,8^.

Src homology 2 domain-containing protein tyrosine phosphatase 1 (SHP-1) is a widely expressed inhibitory protein tyrosine phosphatase (PTP)^9^. It has been implicated in inhibiting tumor development in various cancer types, such as lymphoma, lung cancer, prostate cancer, and breast cancer^10–12^. Evidence suggests that SHP-1 may contribute to the inhibition of aberrant innate immune cell activity. However, whether SHP-1 can inhibit the M2 polarization of TAMs remains largely unknown. The specific regulatory mechanisms of SHP-1 in liver cancer are still largely unknown. Therefore, this study aims to investigate the role and mechanism of SHP-1 in the progression of liver cancer, in order to provide new clues for a better understanding of liver cancer development and lay a foundation for the development of liver cancer treatment strategies based on SHP-1 regulatory pathways (Supplementary Information).

## Methods

### Animal modeling and grouping

Male Balb/c nude mice aged 6–8 weeks were purchased from Henan Scitek Bio-technology Co., Ltd. The mice were kept in a specific pathogen-free (SPF) animal facility with ad libitum access to water and food, under a 12-h light/12-h dark cycle. All animals received humane care. The study followed the guidelines for the ethical and humane use of laboratory animals, and all animal procedures were approved by the Medical Ethics Committee, Bethune International Peace Hospital (2021-KY-79). All methods reported in this manuscript are in accordance with ARRIVE guidelines.

Sixteen mice were randomly divided into two groups: THP-1-NC mimic and THP-1-SHP-1 mimic groups, with 8 mice in each group. The model group was induced to develop liver cancer by intraperitoneal injection of DEN (Sigma, N0258, i.p. 25 mg/kg)^13^. The THP-1-SHP-1 mimic group was injected with THP-1 cells infected with SHP-1 mimic. The cell suspension (5 × 10^6^) was injected into the subcutaneous area of the upper right abdomen^14^. The mice were euthanized 21 days later (mice were deeply anesthetized and euthanized by cervical dislocation). Tumor tissues from the mice were collected, stained with hematoxylin and eosin (HE), embedded in paraffin, sectioned, and stained with suoxin red for 4 min. After rinsing, the slides were stained, mounted with resin, and observed and photographed under a microscope.

### Cell culture

Transfection: SMMC-7721, a human liver cancer cell line, was purchased from Thermo Fisher Scientific Inc. (Shanghai, China, 61870036), THP-1, a human monocytic leukemia cell line, was purchased from Wuhan Punoise Life Technologies Co., Ltd. (Wuhan, China, CL-0233), RAW264.7, a mouse monocyte macrophage leukemia cell line, was purchased from Wuhan Punoise Life Technologies Co., Ltd. (Wuhan, China,CL-0190). SHP-1 mimic and NC mimic were purchased from Genecopoeia (Guangzhou, China). Mimics were diluted with serum-free dilution solution and mixed thoroughly with EntransterTM-R4000. After incubating at room temperature for 15 min, the transfection complex was dropped onto THP-1 cells and incubated for 24 h.

Macrophage were seeded at a density of 1 × 10^6^ cells/mL in RPMI 1640 complete medium treated with PMA (100 ng/mL) for 6 h, followed by the addition of IL-13 (20 ng/mL) and IL-4 (20 ng/mL) for 18 h to generate M2 polarized macrophages.

The co-culture system of macrophages and HCC cells: PMA-treated THP-1 macrophages were seeded into the upper insert of a 6-well Transwell device and co-cultured with SMMC-7721 cells in the lower chamber of a 6-well plate. The two cell types did not directly contact each other. After co-culturing for 16 h, the cells were co-cultured with GM-CSF or without GM-CSF (25 ng/mL R&D Systems) and under normoxic (20% O_2_) or hypoxic (1% O_2_) conditions for 24 h.

### Western blot

RIPA lysis buffer (Thermo Fisher Scientific) was used to lyse and extract proteins from THP-1 and SMMC7721 cells. The protein concentration was determined using a BCA protein assay kit (KeyGen Biotech). Then, 20 μg of protein was separated by 10% SDS-PAGE gel electrophoresis and transferred to PVDF membranes. The membranes were incubated with 5% skim milk for 2 h, followed by overnight incubation at 4 °C with primary antibodies. After incubation with secondary IgG (1:5000, Abcam) at room temperature for 2 h, the protein bands were quantified using ImageJ software. The primary antibodies used were SHP-1 (1:1000, Abcam), p-SHP-1 (1:1000, Thermo Fisher Scientific Inc.), SHP-2 (1:1000, Abcam), p-SHP2 (1:1000, Abcam), HOXA10 (1:1000, Abcam), TGFβ (1:1000, Abcam), MMP2 (1:1000, Abcam), MMP-9 (1:200, Abcam), p-STAT3 (1:1000, Abcam), p-STAT6 (1:1000, Abcam), IL-10 (1:1000, Abcam), IL-4 (1:2000, Abcam), and GAPDH (1:1000, Abcam).

### Cell scratching and immunofluorescence

Single-cell suspensions were dropped onto culture plates containing slides, and after 24 h of incubation at 37 °C in a 5% CO_2_ incubator, the slides were taken out. The cells were fixed with 4% paraformaldehyde for 30 min, blocked with 5% goat serum at room temperature for 30 min, incubated with p-SHP-1 antibody (Abcam) overnight at 4 °C, and then incubated with fluorescent goat anti-rabbit IgG secondary antibody (diluted 1:500 in PBS) (Abcam, ab150081) in a dark environment at room temperature for 1 h. Images were captured using a fluorescence microscope.

### Cell scratching

HCC cells from the co-culture system were collected and suspended in DMEM. After seeding the cells in a 6-well culture plate, a scratch was made using a 200 μL plastic pipette tip. The width of the scratch was measured and recorded by microscopy after 0 h, and the cells were incubated in the incubator for 48 h before measuring the scratch width again.

### Transwell assay

SMMC-7721 cells were resuspended in serum-free medium and seeded in the upper chamber of a Transwell device. The lower chamber was pre-filled with 10% FBS medium. After incubation at room temperature for 24 h, the cells were fixed with 4% paraformaldehyde for 10 min, stained with 0.1% crystal violet for 20 min, and counted under a microscope.

### Cell cloning formation

The co-cultured cells were digested with trypsin-collagenase and then diluted to a density of 1 × 10^4^/mL. The cells were seeded in a 6-well plate at a density of 400 cells per well and cultured for 2 weeks. The cells were fixed with 4% paraformaldehyde for 30 min, stained with 0.1% crystal violet for 15 min, and photographed.

### CCK8 assay

Cell suspensions were prepared using culture medium containing 5% fetal bovine serum. Then, 100 μL of cell suspension was seeded in a 96-well plate, and 10 μL of CCK-8 solution was added to each well. After incubation at 37 °C and 5% CO_2_ for 4 h, the absorbance at 450 nm was measured.

### Statistical analysis

The data are presented as the mean ± standard deviation (SD) of at least three independent experiments. Multiple group comparisons were performed using one-way analysis of variance (ANOVA), and two-group comparisons were analyzed using *t*-tests. Statistical analysis was conducted using GraphPad Prism software. *P < 0.05 was considered statistically significant.

## Results

### Upregulation of TAM SHP-1 inhibits the progression of liver cancer

**Figure 1:**
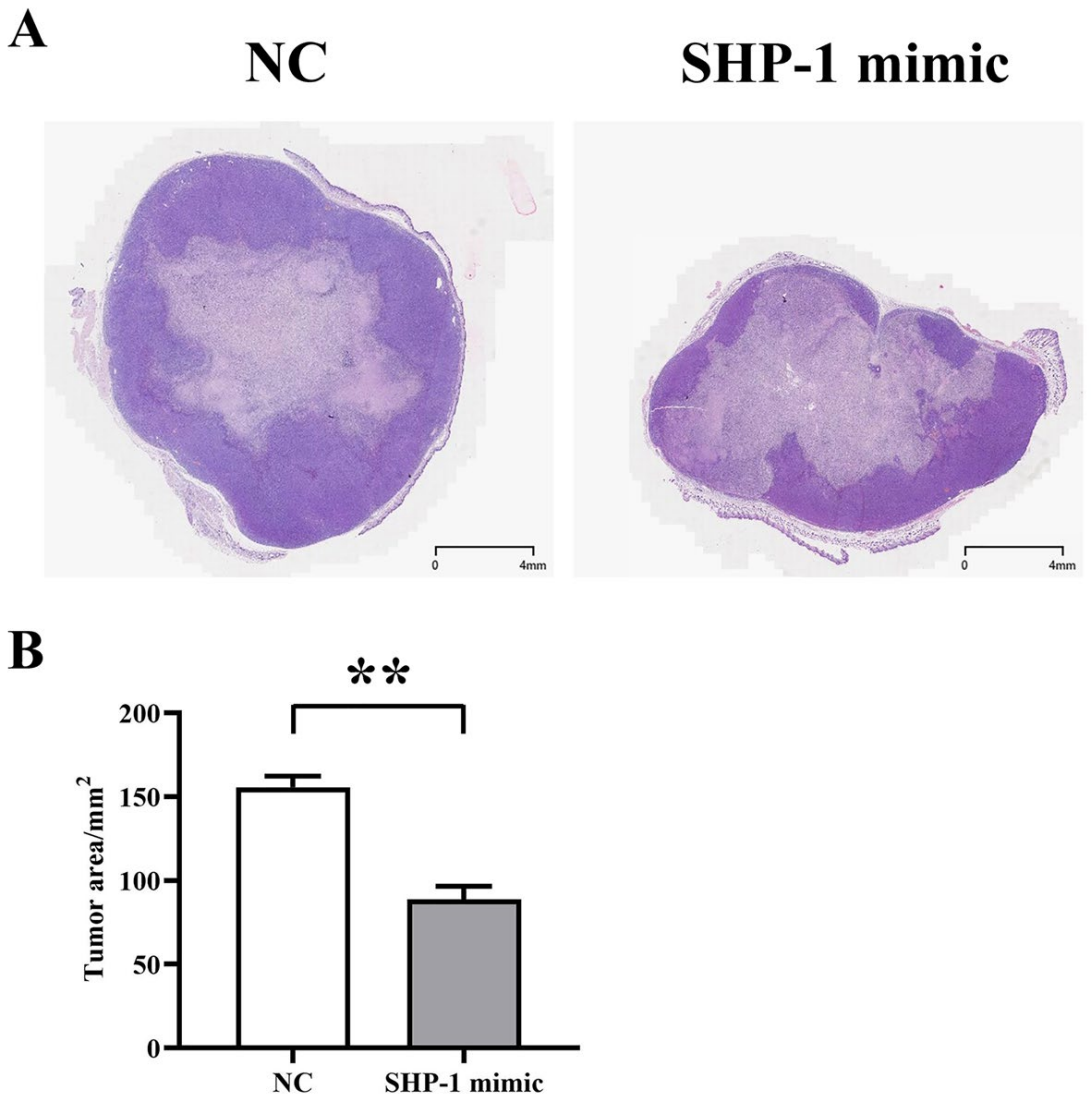
Representative images showing the inhibition of liver cancer progression in mice by overexpression of SHP-1. (**A**) Comparison of tumor volume between the two groups of mice. (**B**) Comparison of tumor area between the two groups of mice.

To study the effects of SHP-1 on tumor development in mice, we injected NC-macrophage and SHP-1 mimic-macrophage into liver cancer mice for animal modeling. The results of HE staining showed that the tumor area in SHP-1 mimic mice was significantly smaller than in NC mice (Fig. 1).

### SHP-1 overexpression promotes SHP2 protein expression in macrophage, and GM-CSF and hypoxia inhibit the expression of p-SHP-1 and p-SHP2

**Figure 2:**
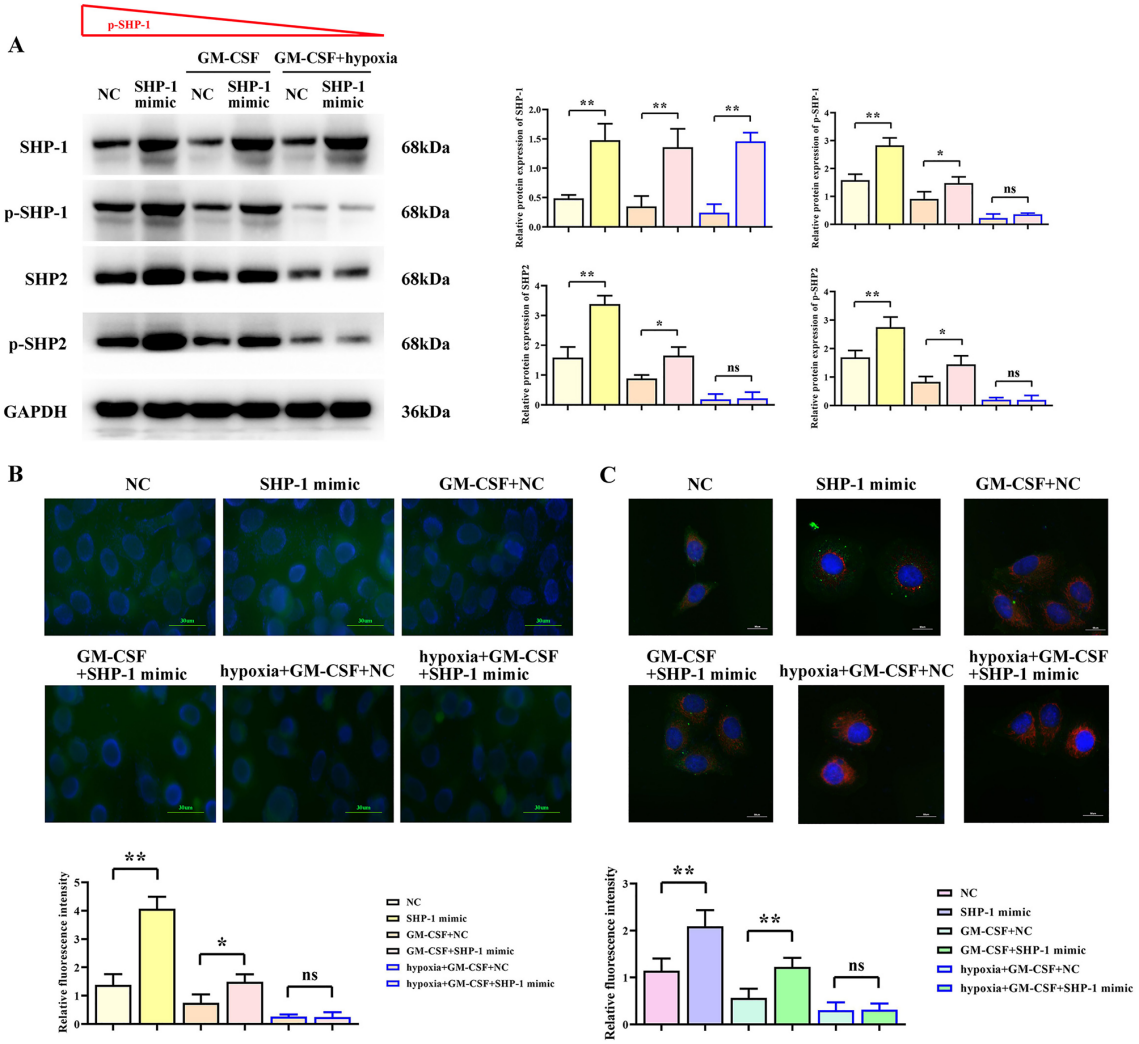
Regulation of protein expression in macrophage by overexpression of SHP-1 and the influence of GM-CSF and hypoxia. (**A**) Western blot results showing the expression of SHP-1, p-SHP-1, SHP2, and p-SHP-2 proteins in macrophage under the influence of SHP-1, GM-CSF, and hypoxia. (**B**) Immunofluorescence staining showing the effect of SHP-1, GM-CSF, and hypoxia on the expression of p-SHP-1 protein in macrophage.

To investigate the effects of GM-CSF and hypoxia on the protein expression of p-SHP-1, SHP2, p-SHP2, we analyzed the expression of SHP-1, p-SHP-1, SHP2, p-SHP2 after GM-CSF and hypoxia interference. The results showed that compared to the NC group, the overexpression of SHP-1 in the SHP mimic group promoted the expression of p-SHP-1, SHP2, and p-SHP2. GM-CSF inhibited the protein expression of p-SHP-1, SHP2, and p-SHP2 in macrophage. When comparing the NC group with the GM-CSF + NC group and the SHP-1 mimic group with the GM-CSF + SHP-1 mimic group, GM-CSF was found to promote the protein expression of p-SHP-1, SHP2, and p-SHP2 in macrophage. Comparing the NC group with the GM-CSF + SHP-1 mimic group, GM-CSF was found to inhibit the regulation of p-SHP-1, SHP2, and p-SHP2 by SHP-1 in macrophage. Hypoxia drives an increase in reactive oxygen species (ROS) in tumor cells^15^, so we added a low oxygen culture experiment to investigate the effects of oxidative stress and GM-CSF on protein expression in macrophage. The combined effect of GM-CSF and oxidative stress significantly enhanced the inhibitory effect of GM-CSF (Fig. 2A). Immunofluorescence staining of p-SHP-1 yielded consistent results (Fig. 2B).

### Effects of SHP-1 overexpression, GM-CSF, and hypoxia on p-HOXA10/TGFβ2 protein expression in macrophage and migration of SMMC7721 cells

**Figure 3:**
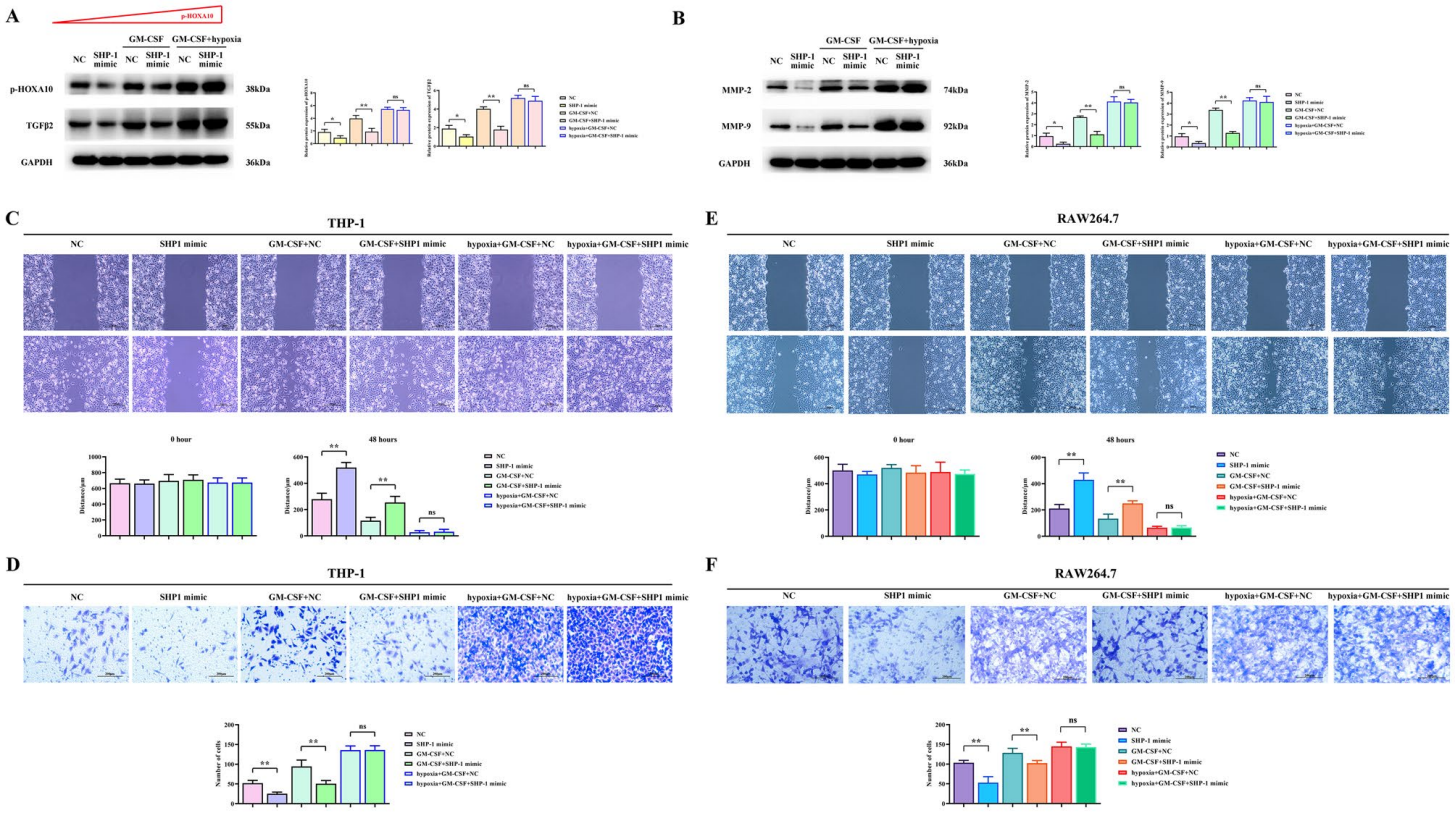
Effects of SHP-1 overexpression, GM-CSF, and hypoxia on the expression of p-HOXA10/TGFβ2 proteins in macrophage and migration of SMMC7721 cells. (**A**) Influence of SHP-1, GM-CSF, and hypoxia on the expression of p-HOXA10/TGFβ2 proteins in macrophage. (**B**) Influence of SHP-1, GM-CSF, and hypoxia on the expression of MMP-2 and MMP-9 proteins in SMMC7721 cells. (**C**) Evaluation of the migration ability of SMMC7721 cells by cell scratch assay under the influence of SHP-1, GM-CSF, and hypoxia. (**D**) Evaluation of the migration ability of SMMC7721 cells by Transwell assay under the influence of SHP-1, GM-CSF, and hypoxia.

To further study the mechanism of SHP-1 inhibition of liver cancer, we analyzed the expression of p-HOXA10/TGFβ2 in macrophage and the migration ability of SMMC7721 cells under the interference of GM-CSF and hypoxia. The results showed that compared to the NC group, SHP-1 inhibited the protein expression of p-HOXA10/TGFβ2 in macrophage. GM-CSF promoted the protein expression of p-HOXA10/TGFβ2 and M2 polarization-related proteins in macrophage and inhibited the inhibitory effect of SHP-1. Under the combined action of GM-CSF and hypoxia, the protein expression of p-HOXA10/TGFβ2 in macrophage was significantly increased, and the inhibitory effect of SHP-1 was insignificant (P > 0.05) (Fig. 3A). SHP-1 inhibited the protein expression of MMP-2 and MMP-9 in liver cancer cells. SHP-1 can inhibit the expression of MMP-2 and MMP-9 protein in hepatocellular carcinoma cells related to migration.. Under the combined action of GM-CSF and oxidative stress, the protein expression of MMP-2 and MMP-9 in liver cancer cells was significantly increased, and the inhibitory effect of SHP-1 was insignificant (Fig. 3B). Cell scratching and Transwell assay results showed that SHP-1 inhibited the migration ability of liver cancer cells, GM-CSF promoted the migration of liver cancer cells and suppressed the inhibitory ability of SHP-1, and the combined action of GM-CSF and oxidative stress promoted the migration of liver cancer cells (Fig. 3C,D).

### Effects of SHP-1 overexpression, GM-CSF, and hypoxia on the expression of M2 polarization-related proteins in macrophage and the proliferation of liver cancer cells

**Figure 4:**
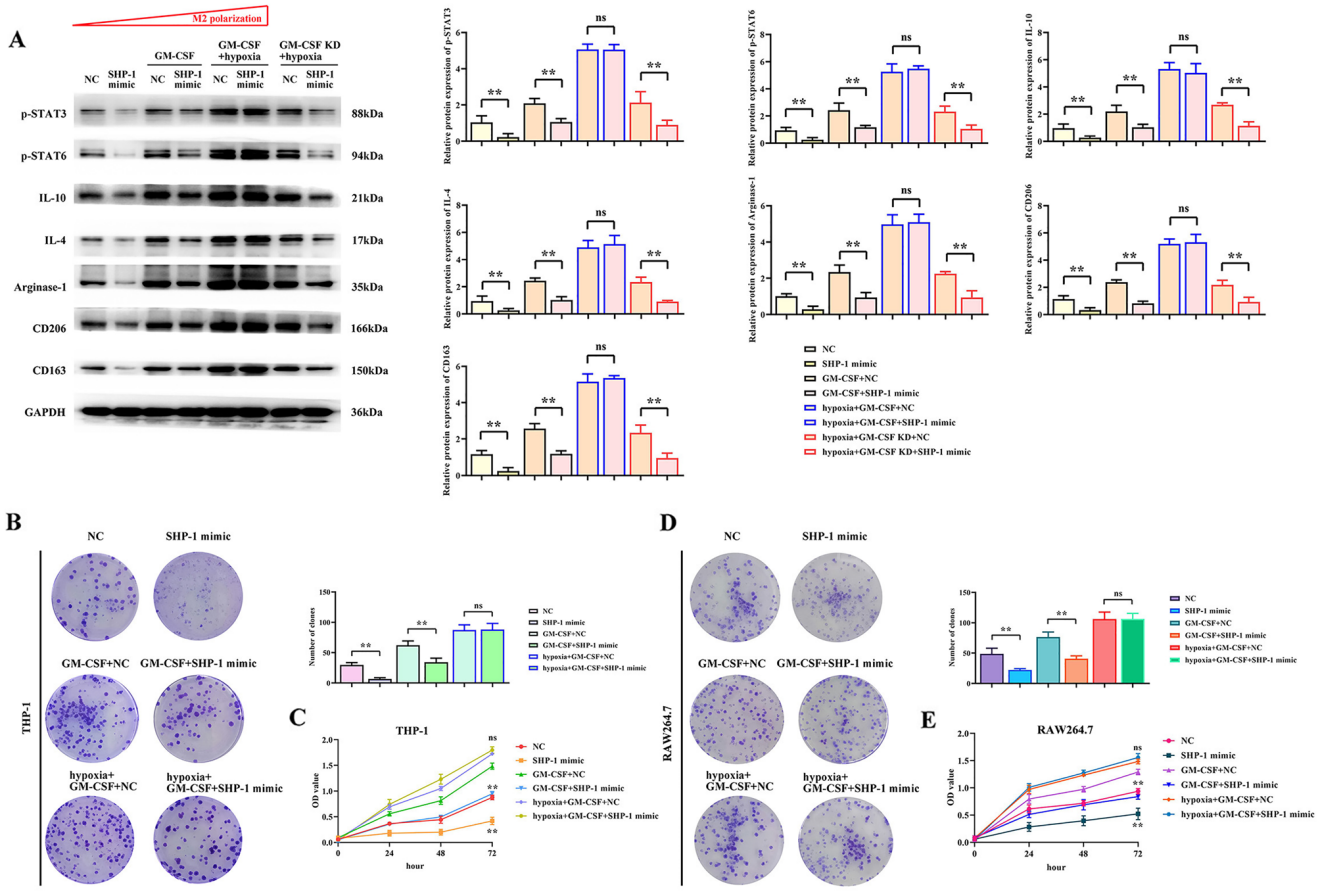
Effects of SHP-1 overexpression, GM-CSF, GM-CSF knockdown, and hypoxia on the expression of M2 polarization-related proteins in macrophage and proliferation of liver cancer cells. (**A**) Influence of SHP-1 overexpression, GM-CSF, GM-CSF knockdown, and hypoxia on the expression of p-STAT3, p-STAT6, IL-10, IL-4, and Arginase-I proteins involved in M2 polarization in macrophage. (**B**) Evaluation of the proliferation ability of SMMC7721 cells by clonogenic assay under the influence of SHP-1, GM-CSF, GM-CSF knockdown, and hypoxia. (**C**) Evaluation of the proliferation ability of SMMC7721 cells by CCK-8 assay under the influence of SHP-1, GM-CSF, GM-CSF knockdown, and hypoxia.

To further study the mechanism of SHP-1 inhibition of liver cancer, we analyzed the expression of M2 polarization-related proteins in macrophage and the proliferation of SMMC7721 cells under the interference of GM-CSF, GM-CSF knockdown and hypoxia. The results showed that compared to the NC group, SHP-1 inhibited the protein expression of p-STAT3, p-STAT6, and M2 polarization-related proteins IL-10, IL-4, Arginase-1, CD206 and CD163 in macrophage. GM-CSF promoted the protein expression of p-STAT3, p-STAT6, IL-10, IL-4, Arginase-1, CD206 and CD163 in macrophage and suppressed the inhibitory effect of SHP-1. Under the combined action of GM-CSF and hypoxia, the protein expression of p-STAT3, p-STAT6, IL-10, IL-4, Arginase-1, CD206 and CD163 in macrophage was significantly increased, and the inhibitory effect of SHP-1 was insignificant (P > 0.05). Protein expression of p-STAT3, p-STAT6 and M2 polarization-associated proteins IL-10, IL-4, Arginase-I, CD206 and CD163 was significantly suppressed in macrophages under the combined effect of GM-CSF knockdown and hypoxia (Fig. 4A).

Cell cloning and CCK8 assay results showed that SHP-1 inhibited the proliferation of liver cancer cells, GM-CSF promoted the proliferation of liver cancer cells and suppressed the inhibitory effect of SHP-1, and the combined action of GM-CSF and oxidative stress promoted the proliferation of liver cancer cells. GM-CSF knockdown and oxidative stress in conjunction with hypoxia inhibits the proliferation of hepatocellular carcinoma cells (Fig. 4B,C).

## Discussion

**Figure 5:**
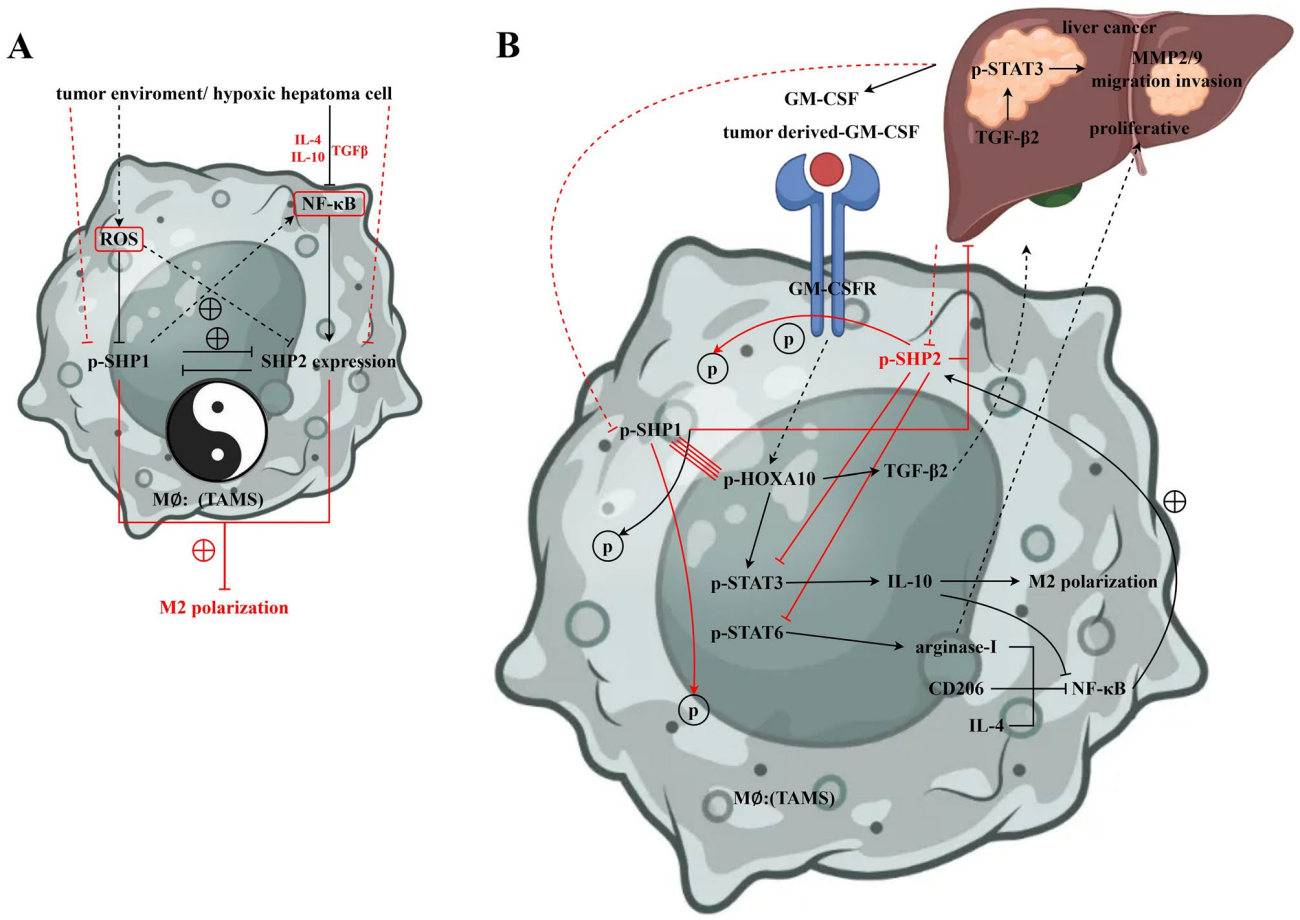
Research on the mechanism of tyrosine phosphatase SHP-1 inhibiting liver cancer progression through the regulation of SHP2/GM-CSF pathway in TAMs.

Liver cancer, as a highly malignant tumor, faces many challenges in its treatment process. Therefore, it is of great significance to search for new treatment strategies and molecular targets. In recent years, immunotherapy has been recognized as having tremendous potential in cancer treatment. This study aimed to explore the role of tyrosine phosphatase SHP-1 in the progression of liver cancer and lay the foundation for the development of liver cancer treatment strategies based on the SHP-1 regulatory pathway. Here, we demonstrated that hypoxia and GM-CSF synergistically promoted M2 polarization and migration and proliferation of liver cancer through the inhibition of SHP-1 and SHP-2 phosphorylation and the increase of p-HOXA10 expression (Fig. 5).

Protein tyrosine phosphatases (PTPs) serve as regulators of cellular functions including differentiation, metabolism, migration, and survival. PTPs counteract tyrosine kinases by removing phosphate molecules from molecular signaling residues, thereby inhibiting signal transduction. Two PTPs, SHP-1 and SHP-2 (protein tyrosine phosphatase 1 and 2 with SH2 domains, respectively), as well as another inhibitory phosphatase, SH2-containing inositol phosphatase (SHIP), are crucial for cellular functions. Recent studies have shown the dysregulation of SHP-1, SHP-2, and SHIP is associated with uncontrolled cell growth and metabolism^16^. In most systems, SHP-1 has been demonstrated as a negative regulator and shown to directly bind to the regulated receptor itself or associated co-receptors, whereas SHP-2 is considered to positively promote signal transduction^17^. However, recent studies have revealed complex associations between SHP-1, SHP-2, and tumor occurrence and development. For example, the research results of Beldi-Ferchiou A showed that the gene expression of SHP-1 and SHP-2 effectively inhibited the activation of the JAK/STAT pathway and induced apoptosis in tumor cells^18^. Xiao-hua Tao’s study demonstrated that SHP-1 and SHP-2 play promoting roles in the development of human papillomavirus-induced condyloma acuminatum and cervical cancer^19^. SHP-1 down-regulated CSF1R expression, which induces macrophage M2 polarization, and inhibited STAT3/NFκB signaling, leading to suppression of macrophage survival and disturbance of macrophage polarization^20^.

It has been shown that tumor-associated macrophages (TAMs), especially M2 subtype macrophages, promote cancer cell metastasis by inducing EMT^21^. M2 polarization of TAMs is a multifactorial, multistep, and complex pathological process. IL-10 is a major cell factor involved in the negative feedback control of inflammation. Yannick Degboé’s research has shown that IL-10 signals through the STAT3 pathway to promote macrophage phenotypic transformation^22^. The anti-inflammatory cytokine IL-10 activates innate immune signaling pathways to promote M2 polarization of macrophages^23^. CD206 and Arginase-I are markers of macrophage polarization^24,25^. Previous studies have shown that STAT can inhibit nuclear factor kappaB (NF-κB) expression through the CD206/Arginase-I/IL-4/IL-10 signaling pathway^26–29^. Importantly, the activation of the NFκB-dependent inflammatory signaling pathway drives SHP2 gene expression^30^.

Our experimental results showed that increasing the expression of SHP-1 can inhibit tumor development in vivo, further confirming the importance of SHP-1 in the suppression of liver cancer, which is consistent with other research results^31^. Furthermore, overexpression of SHP-1 promoted the expression of SHP2/GM-CSF pathway-regulated proteins, including p-SHP-1, SHP2, and p-SHP2. Both GM-CSF and hypoxia regulated the expression of these proteins, with GM-CSF inhibiting the regulatory role of SHP-1 in macrophage. To investigate the effects of GM-CSF and hypoxia-induced oxidative stress on SHP-1, we conducted further studies. In this study, we found that SHP-1 lost its inhibitory effect on the expression of proteins p-STAT3, p-STAT6, IL-10, IL-4, and Arginase-I associated with M2 polarization in macrophages under the combined effect of GM-CSF and hypoxic environment. In addition, GM-CSF also promoted the expression of p-HOXA10 and TGFβ2 in liver cancer cells and inhibited the inhibitory effect of SHP-1. These results further support the important role of SHP-1 in regulating the progression of liver cancer.

Although we focused on p-HOXA10 and TGF-β2, we also recognize the possible effects of other M2-type polarization-related cytokines or proteins on SHP-1. Our study did not exclude the presence of these factors, but rather focused on analyzing the effects of GM-CSF and the hypoxic environment on the SHP-1 regulatory network. Future studies could further explore the role of other cytokines or proteins in this process to fully understand the complex mechanisms of hepatocellular carcinoma development and immune regulation.

In summary, this study revealed the mechanism by which SHP-1 inhibits the progression of liver cancer by regulating the SHP2/GM-CSF pathway in TAMs. Overexpression of SHP-1 can inhibit the proliferation and migration ability of liver cancer cells and suppress the expression of M2 polarization-related proteins. However, under the combined action of GM-CSF and hypoxia, the inhibitory effect of SHP-1 becomes limited and contributes to the proliferation and migration of liver cancer cells. These research findings provide new clues for the development of liver cancer treatment strategies based on the SHP-1 regulatory pathway and offer new avenues for improving the effectiveness of liver cancer treatment. However, further research is needed to validate these findings and explore deeper molecular mechanisms.

### Supplementary Information

Supplementary Information 1.

Supplementary Information 2.

## Data Availability

This published article contains all of the data that were examined throughout this investigation.
