# Supplementary figures and images for: Mechanism study of tyrosine phosphatase shp-1 in inhibiting hepatocellular carcinoma progression by regulating the SHP2/GM-CSF pathway in TAMs
Source: Sci Rep. 2024 Apr 21;14:9128. doi: 10.1038/s41598-024-59725-w (PMC11033275; doi:10.1038/s41598-024-59725-w)

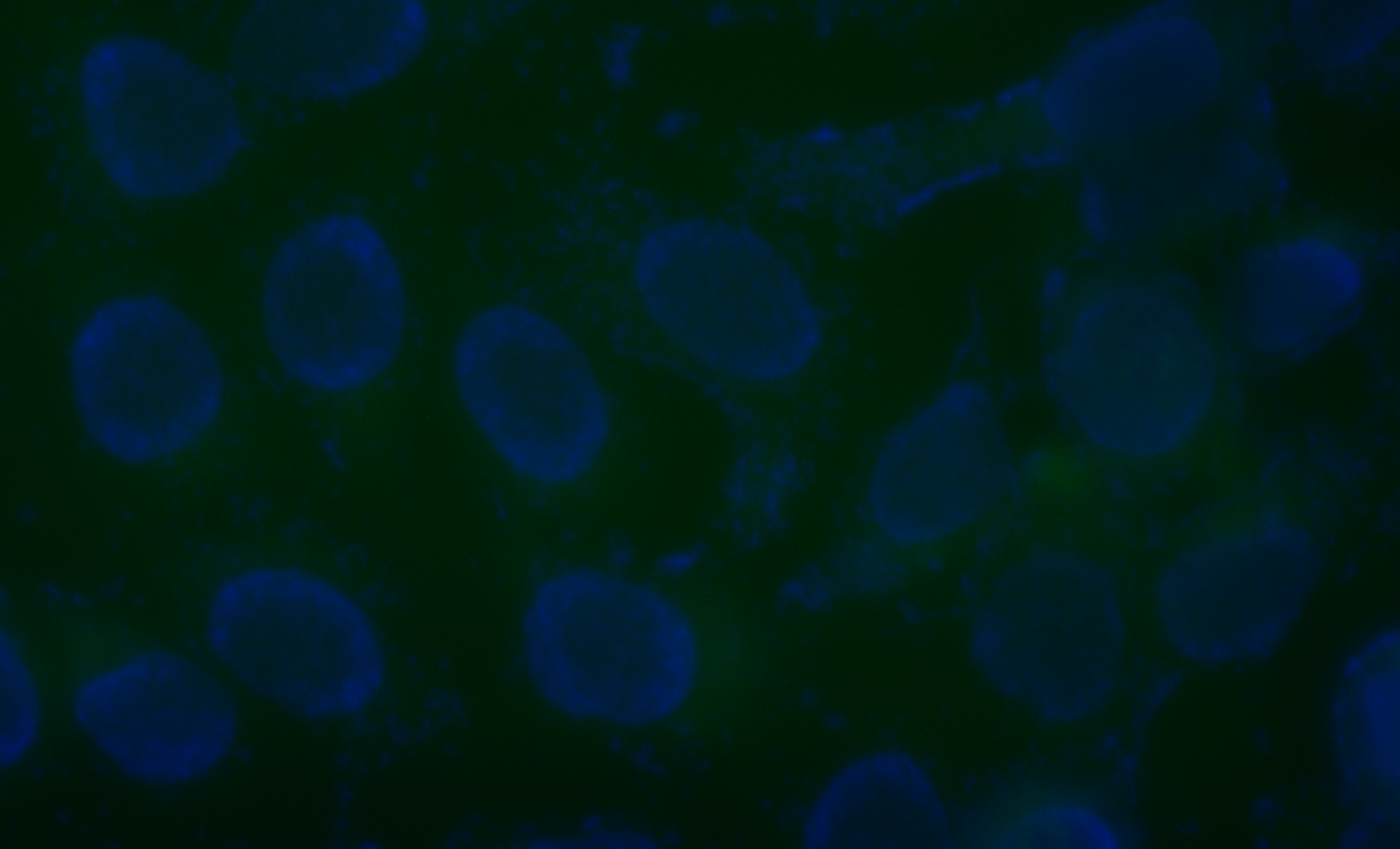

Supplement: Supplementary file 1 — Supplementary Information 1. [file 41598_2024_59725_MOESM1_ESM.zip › Original diagram of the cell experiment/fig2B/1.tif]

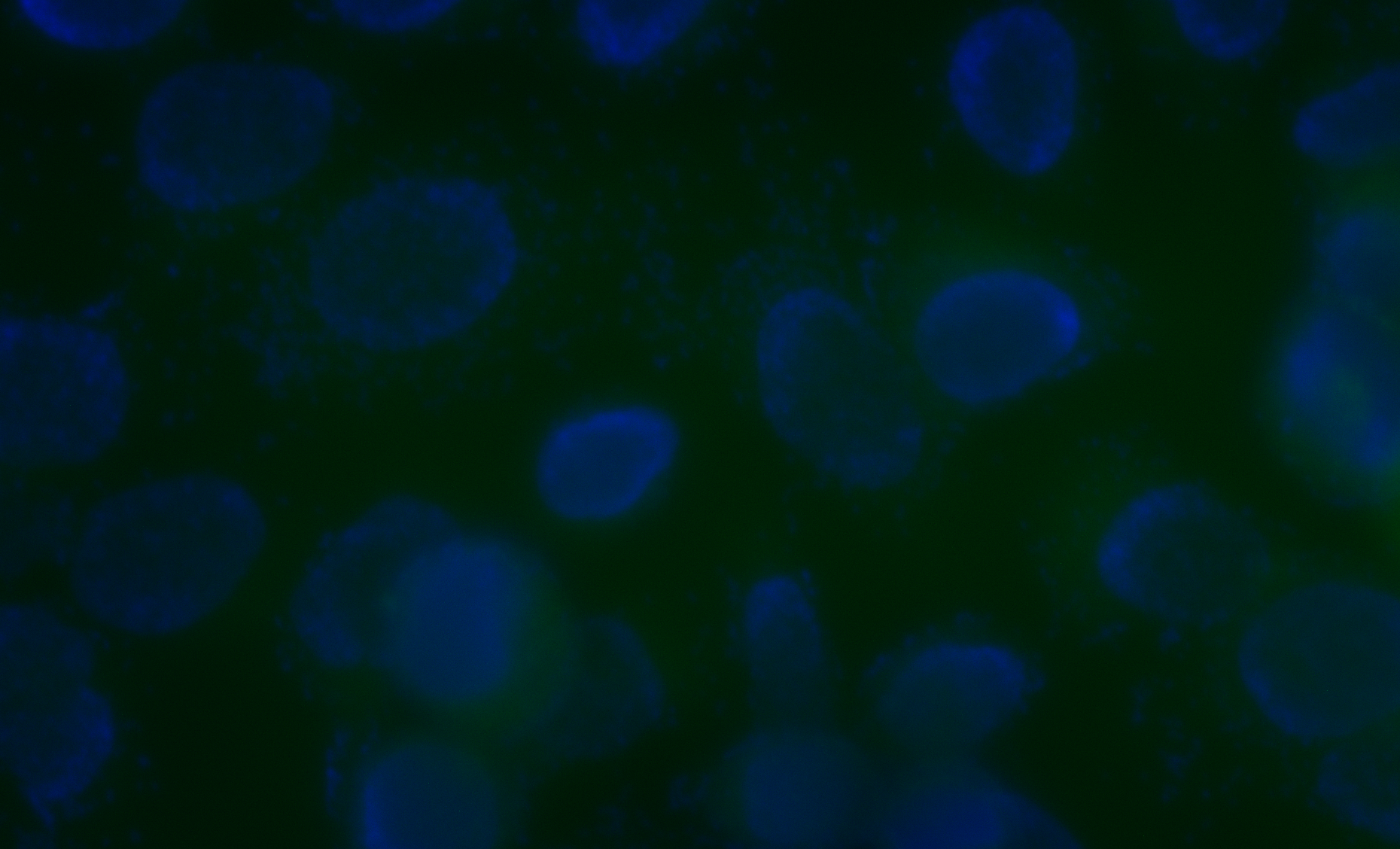

Supplement: Supplementary file 1 — Supplementary Information 1. [file 41598_2024_59725_MOESM1_ESM.zip › Original diagram of the cell experiment/fig2B/2.tif]

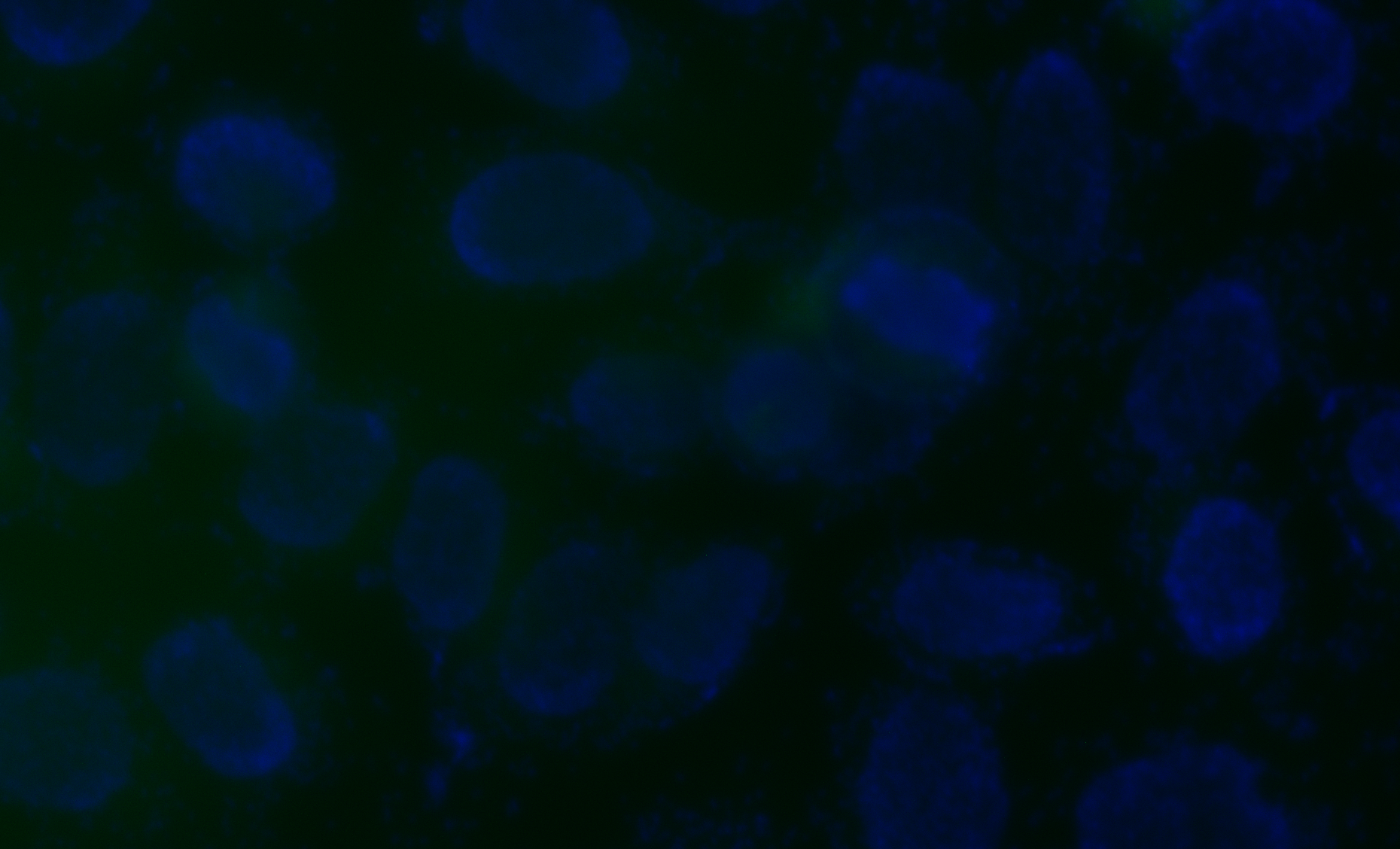

Supplement: Supplementary file 1 — Supplementary Information 1. [file 41598_2024_59725_MOESM1_ESM.zip › Original diagram of the cell experiment/fig2B/3.tif]

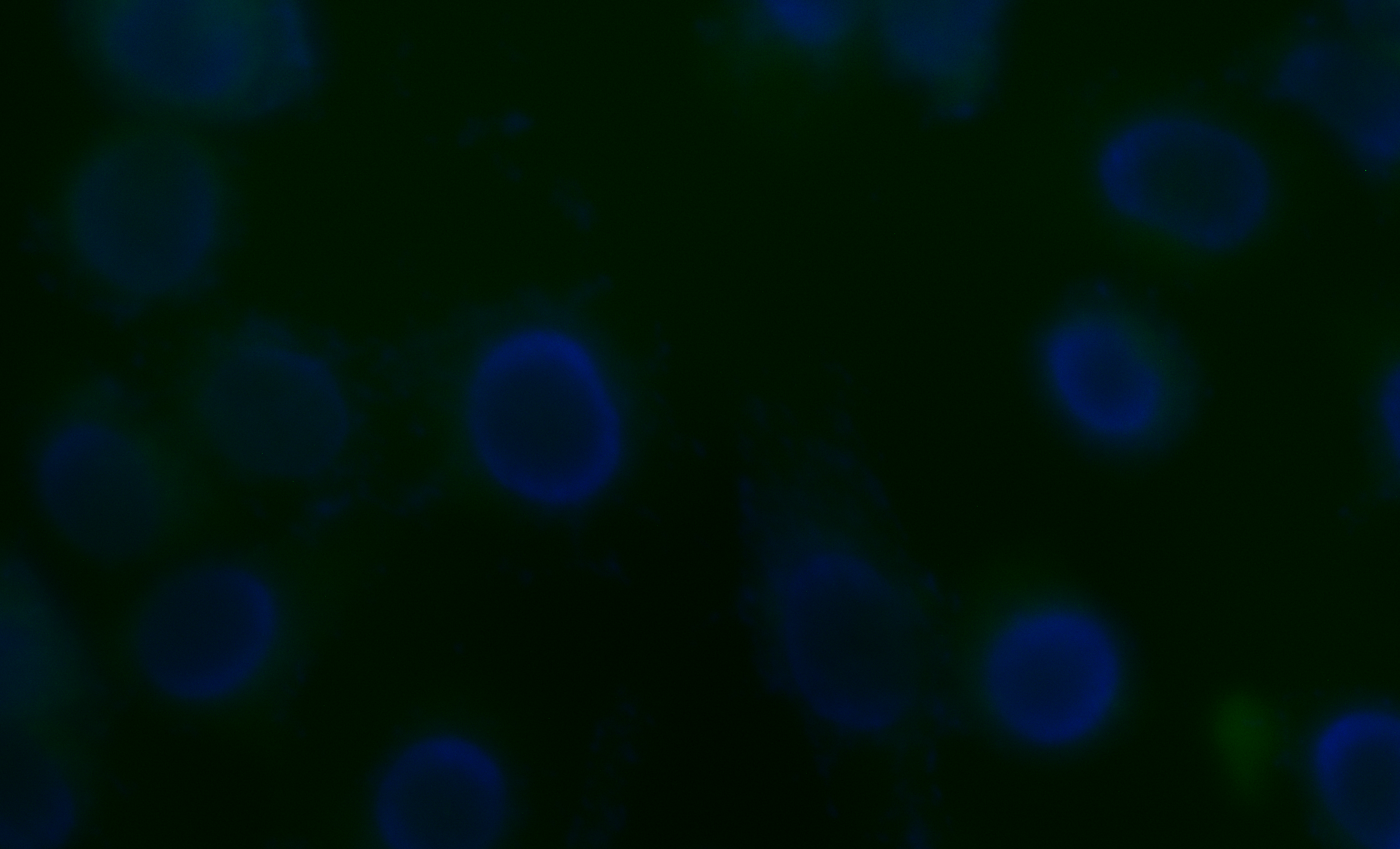

Supplement: Supplementary file 1 — Supplementary Information 1. [file 41598_2024_59725_MOESM1_ESM.zip › Original diagram of the cell experiment/fig2B/4.tif]

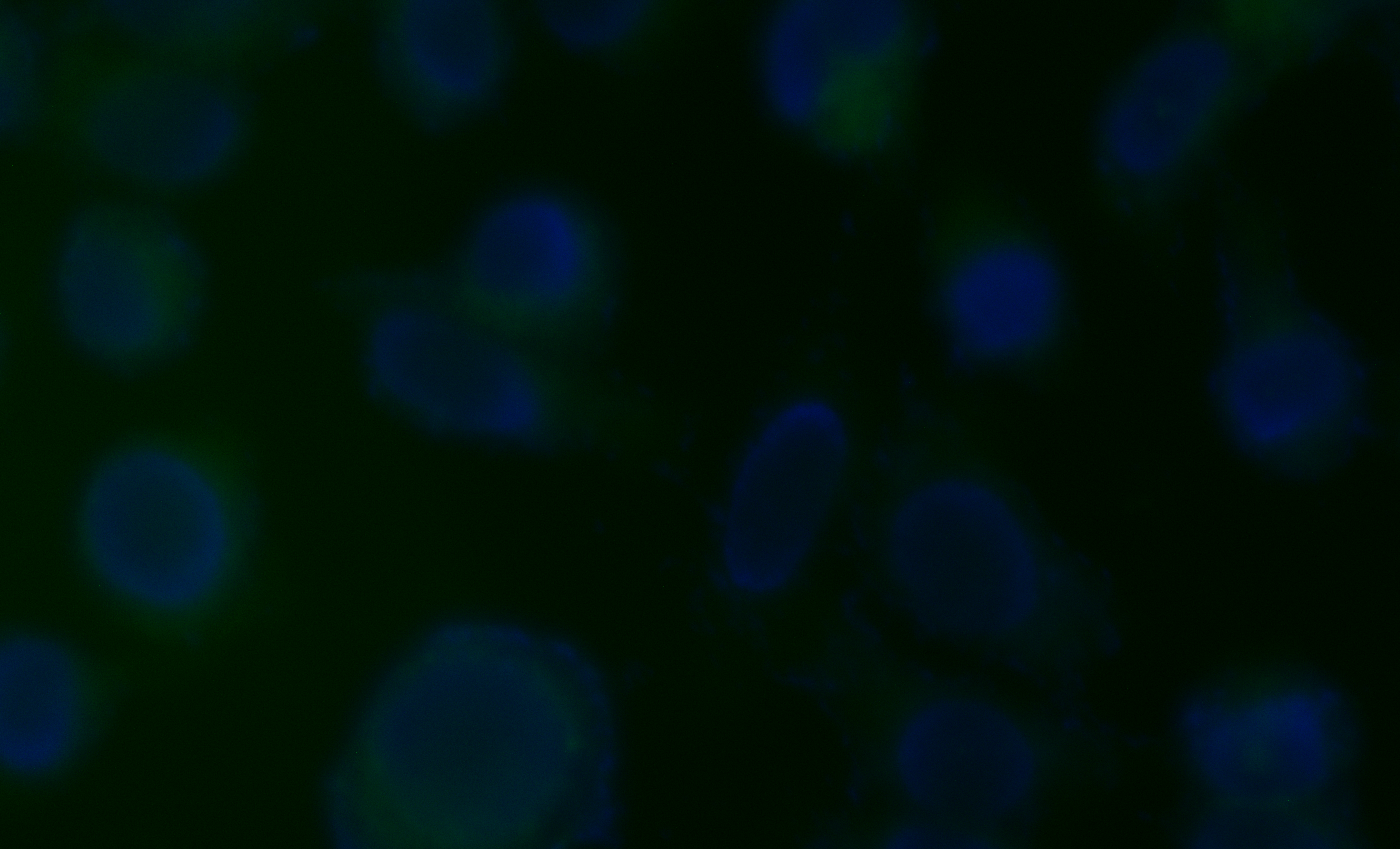

Supplement: Supplementary file 1 — Supplementary Information 1. [file 41598_2024_59725_MOESM1_ESM.zip › Original diagram of the cell experiment/fig2B/5.tif]

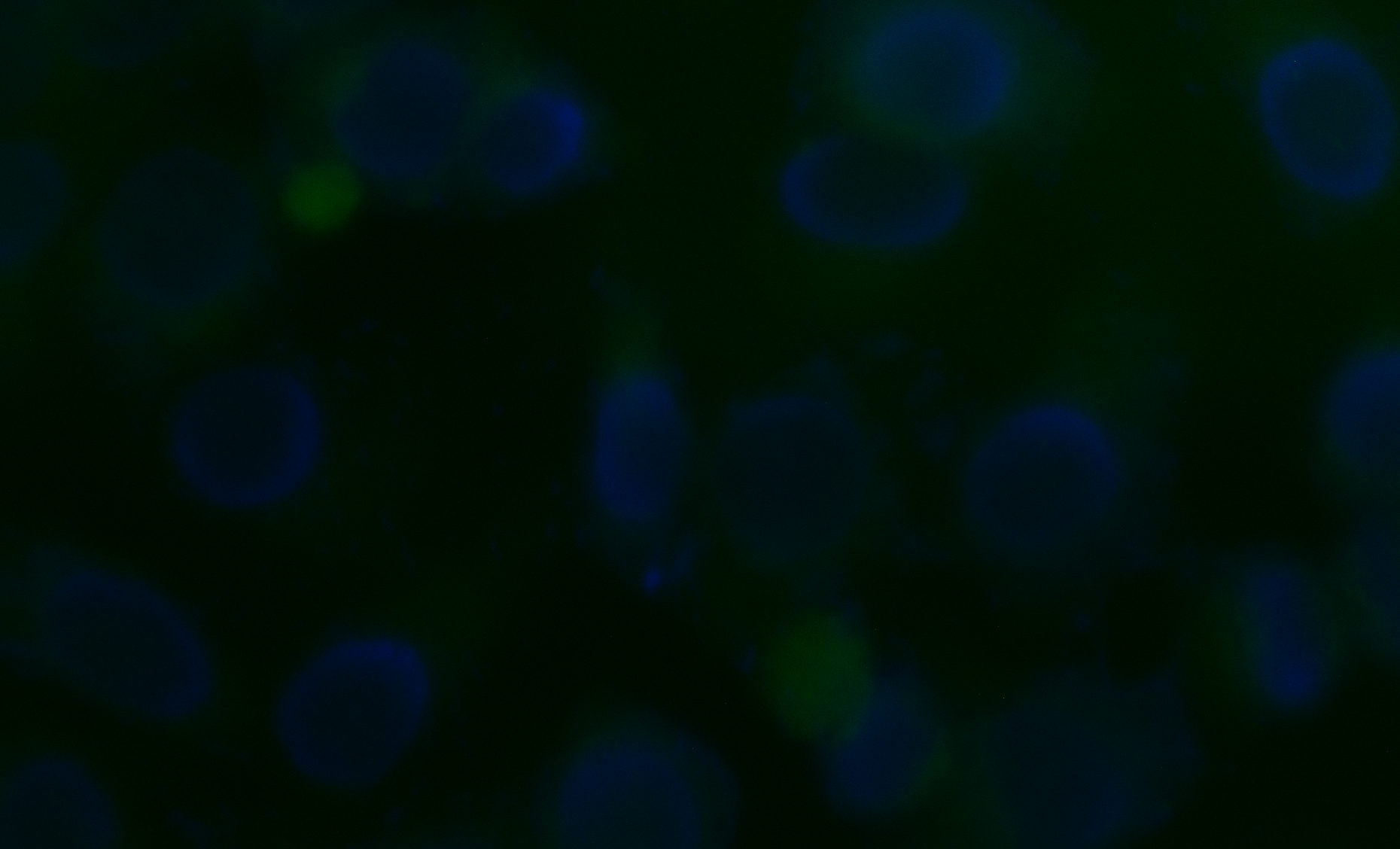

Supplement: Supplementary file 1 — Supplementary Information 1. [file 41598_2024_59725_MOESM1_ESM.zip › Original diagram of the cell experiment/fig2B/6.tif]

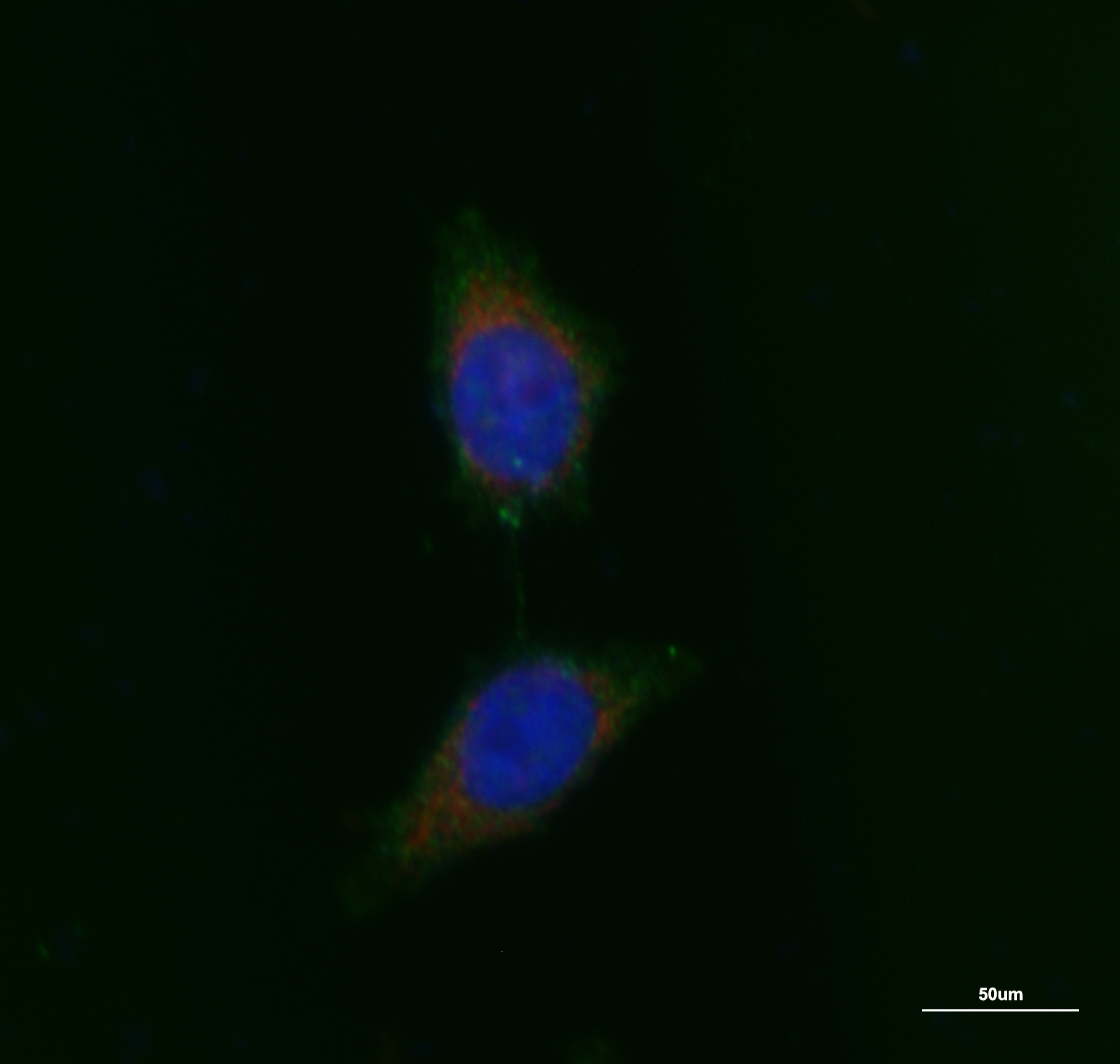

Supplement: Supplementary file 1 — Supplementary Information 1. [file 41598_2024_59725_MOESM1_ESM.zip › Original diagram of the cell experiment/fig2C/1.tif]

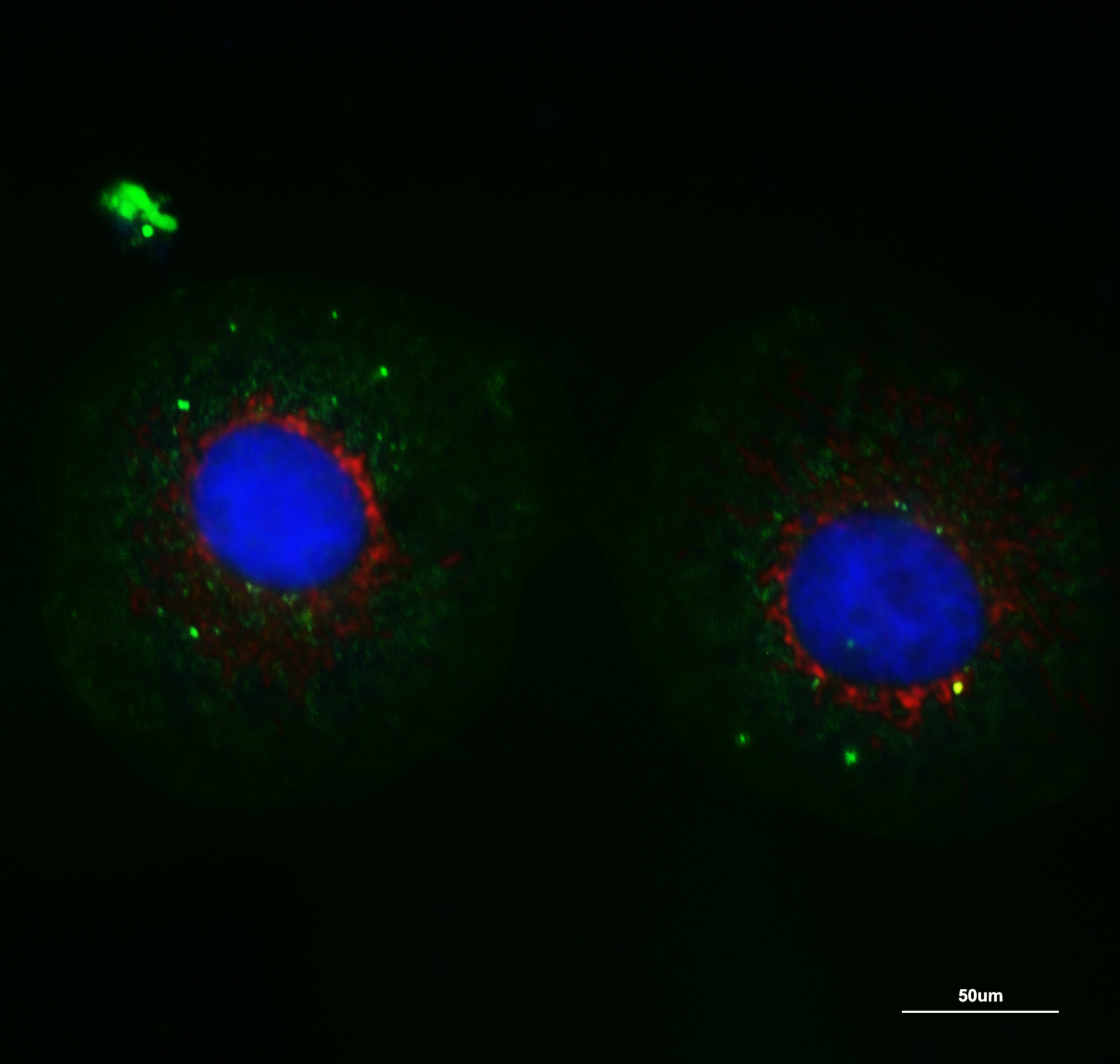

Supplement: Supplementary file 1 — Supplementary Information 1. [file 41598_2024_59725_MOESM1_ESM.zip › Original diagram of the cell experiment/fig2C/2.tif]

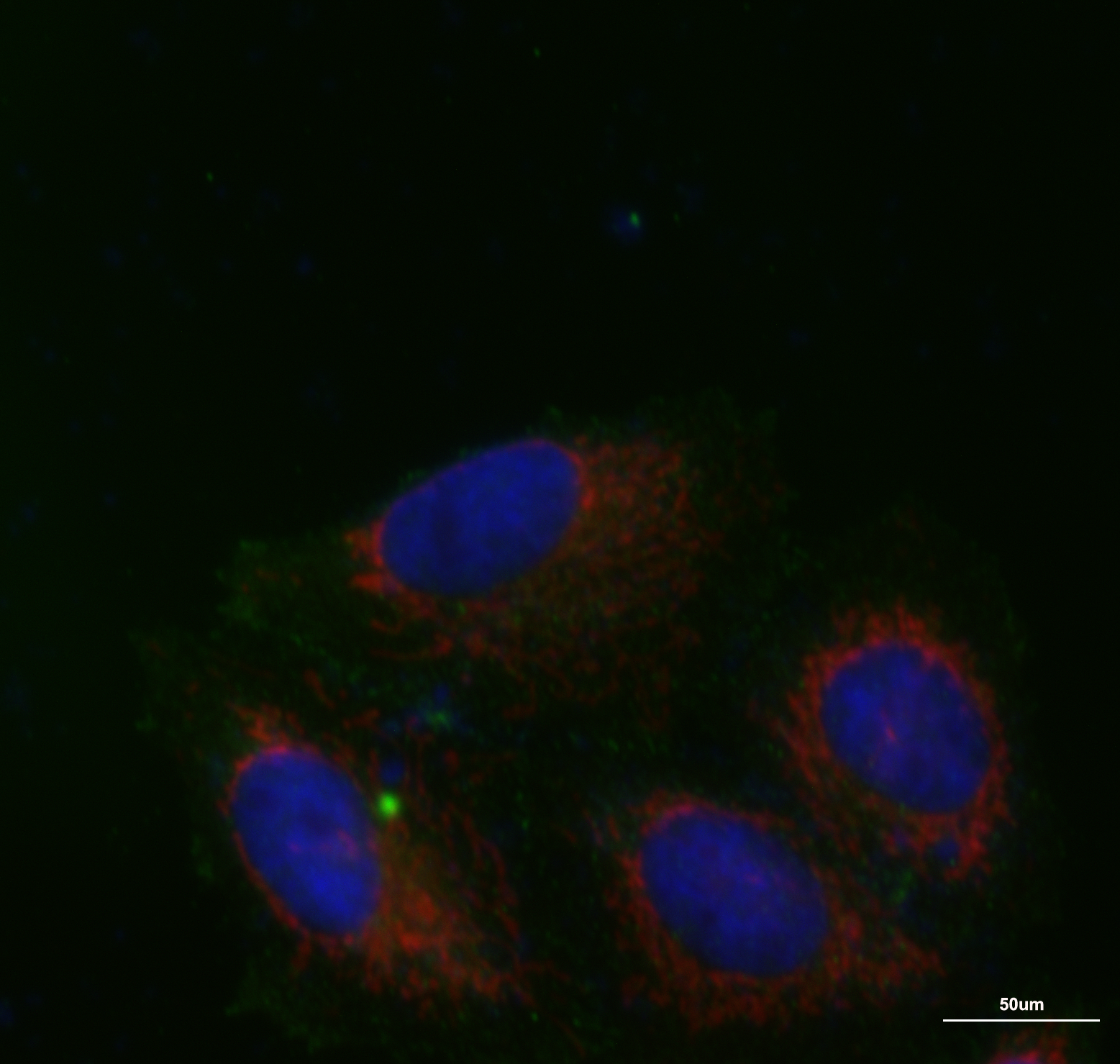

Supplement: Supplementary file 1 — Supplementary Information 1. [file 41598_2024_59725_MOESM1_ESM.zip › Original diagram of the cell experiment/fig2C/3.tif]

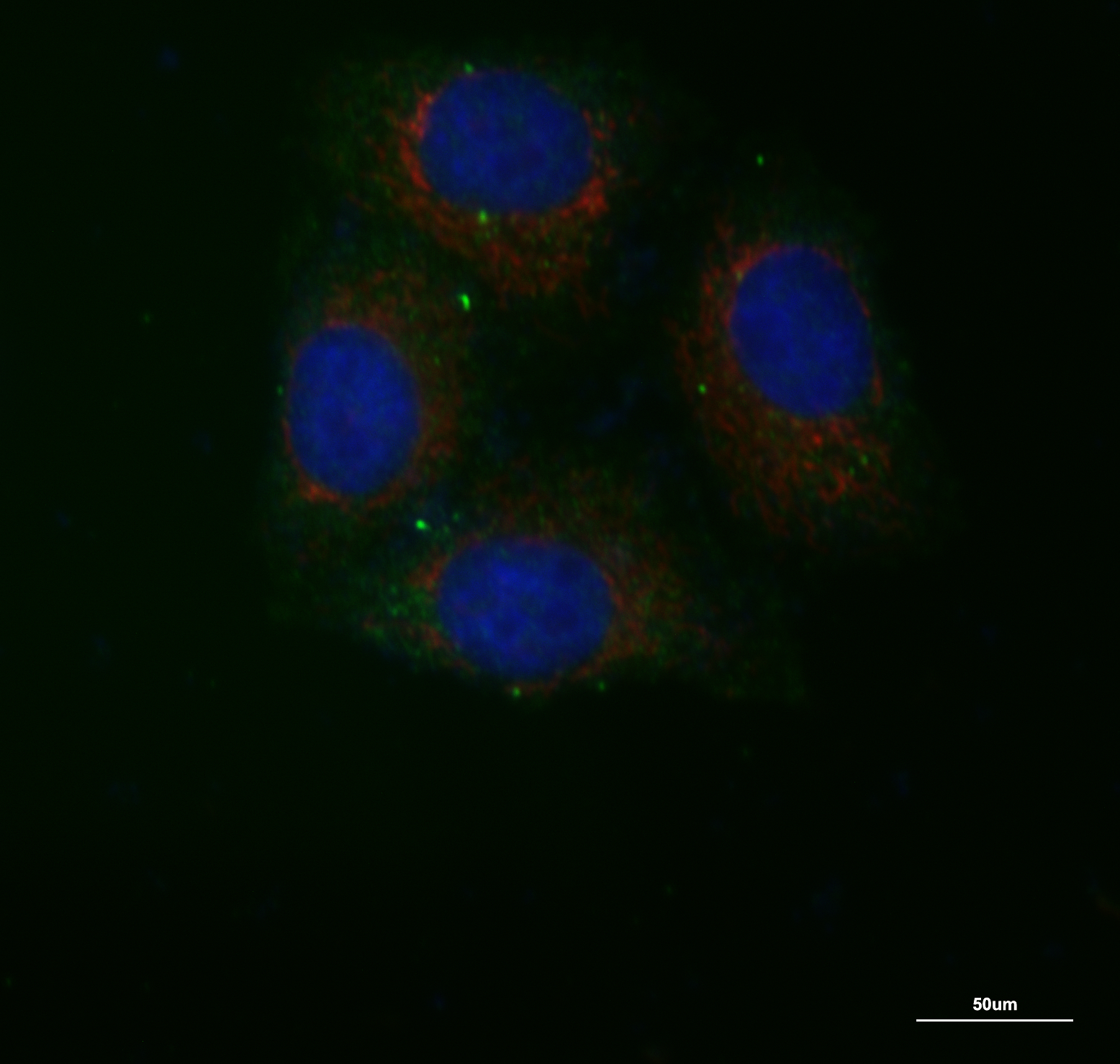

Supplement: Supplementary file 1 — Supplementary Information 1. [file 41598_2024_59725_MOESM1_ESM.zip › Original diagram of the cell experiment/fig2C/4.tif]

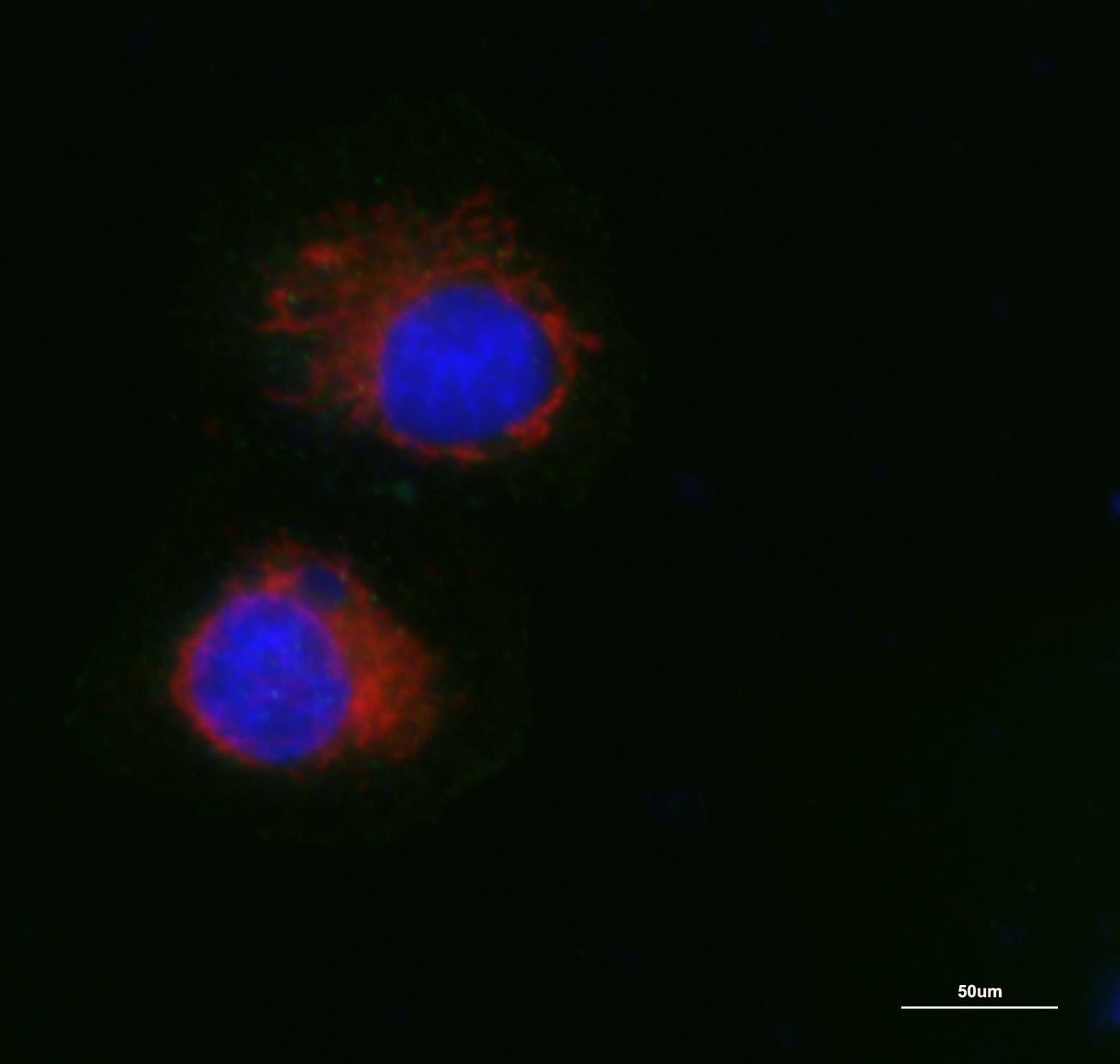

Supplement: Supplementary file 1 — Supplementary Information 1. [file 41598_2024_59725_MOESM1_ESM.zip › Original diagram of the cell experiment/fig2C/5.tif]

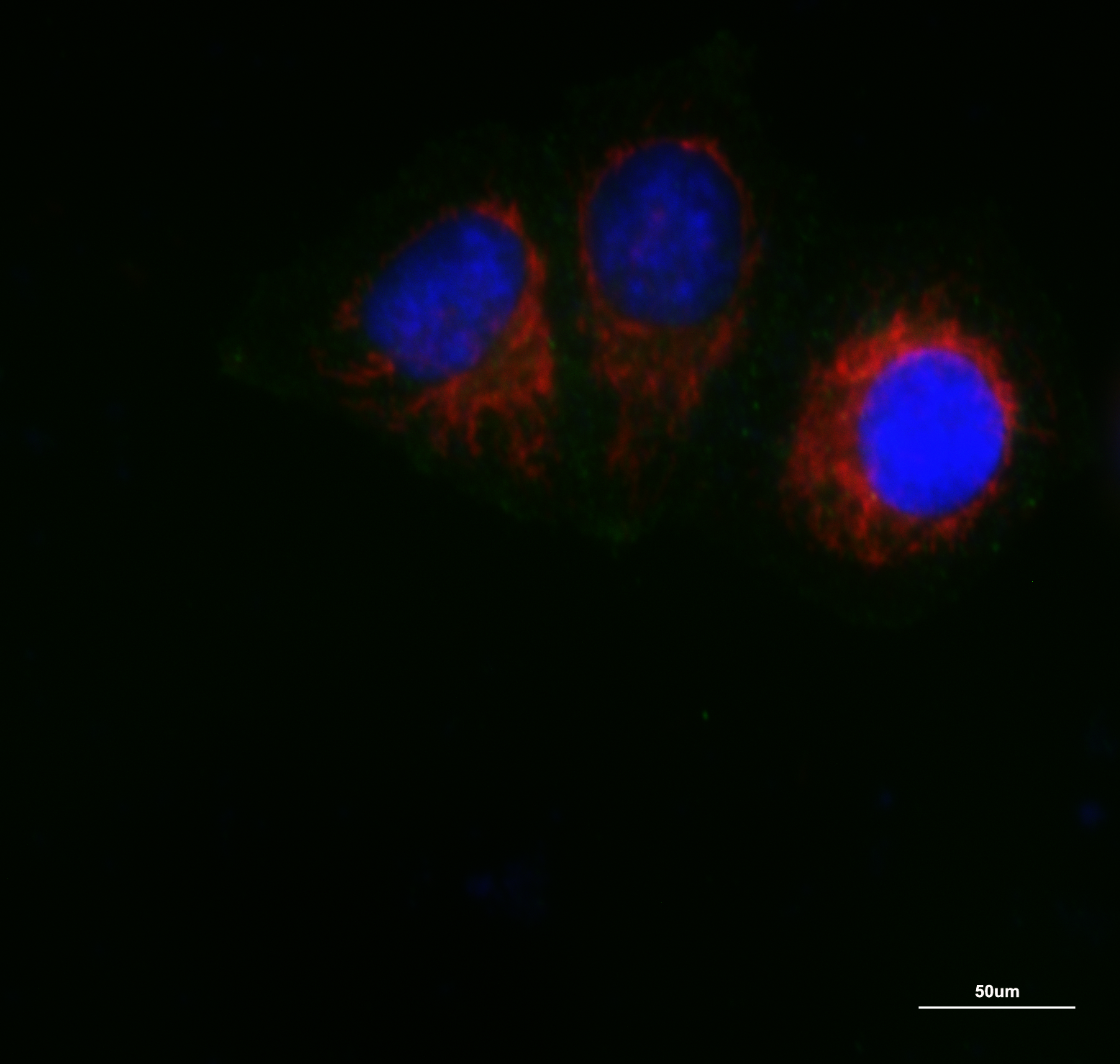

Supplement: Supplementary file 1 — Supplementary Information 1. [file 41598_2024_59725_MOESM1_ESM.zip › Original diagram of the cell experiment/fig2C/6.tif]

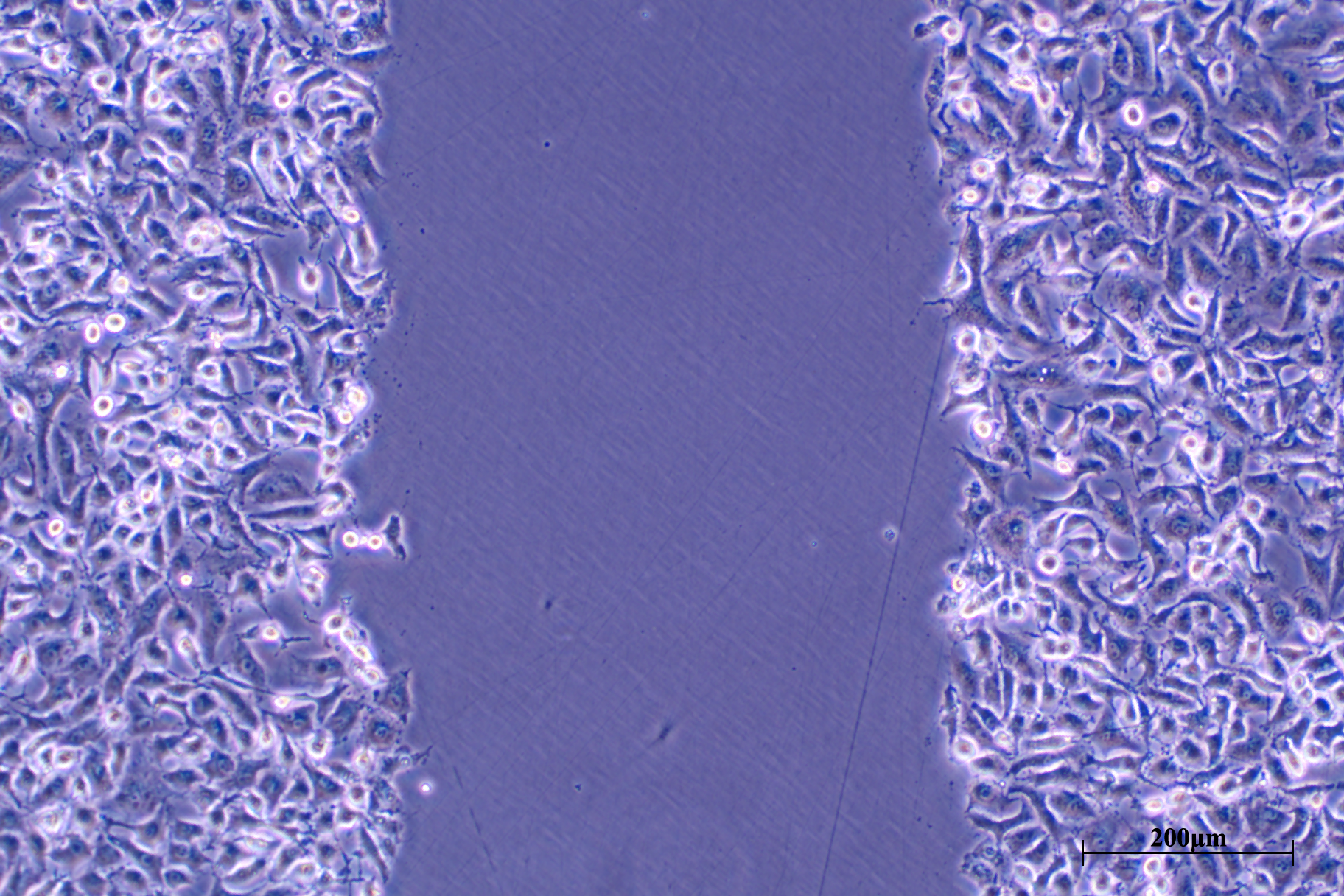

Supplement: Supplementary file 1 — Supplementary Information 1. [file 41598_2024_59725_MOESM1_ESM.zip › Original diagram of the cell experiment/fig3C/0 HOUR/0007.tif]

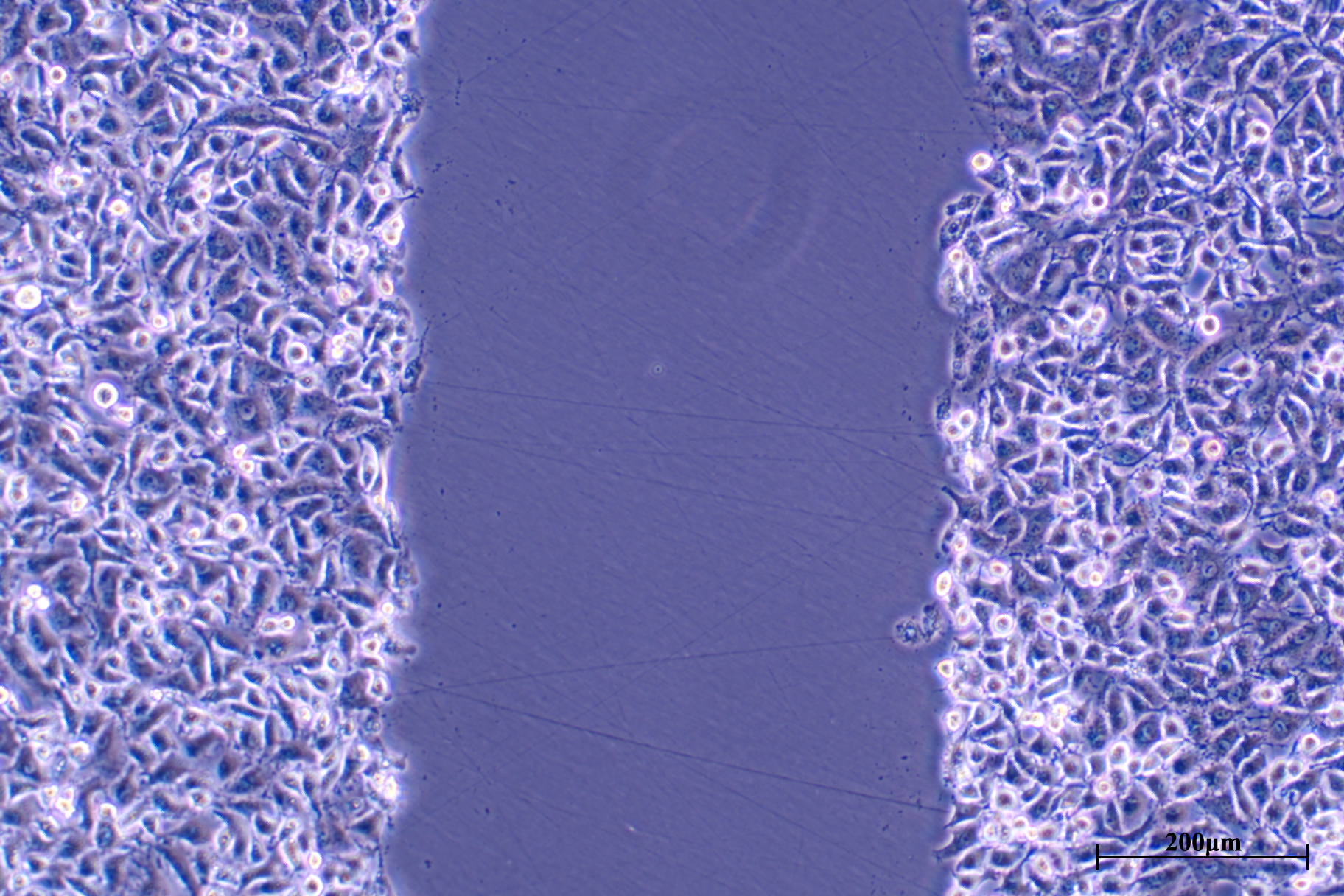

Supplement: Supplementary file 1 — Supplementary Information 1. [file 41598_2024_59725_MOESM1_ESM.zip › Original diagram of the cell experiment/fig3C/0 HOUR/0008.tif]

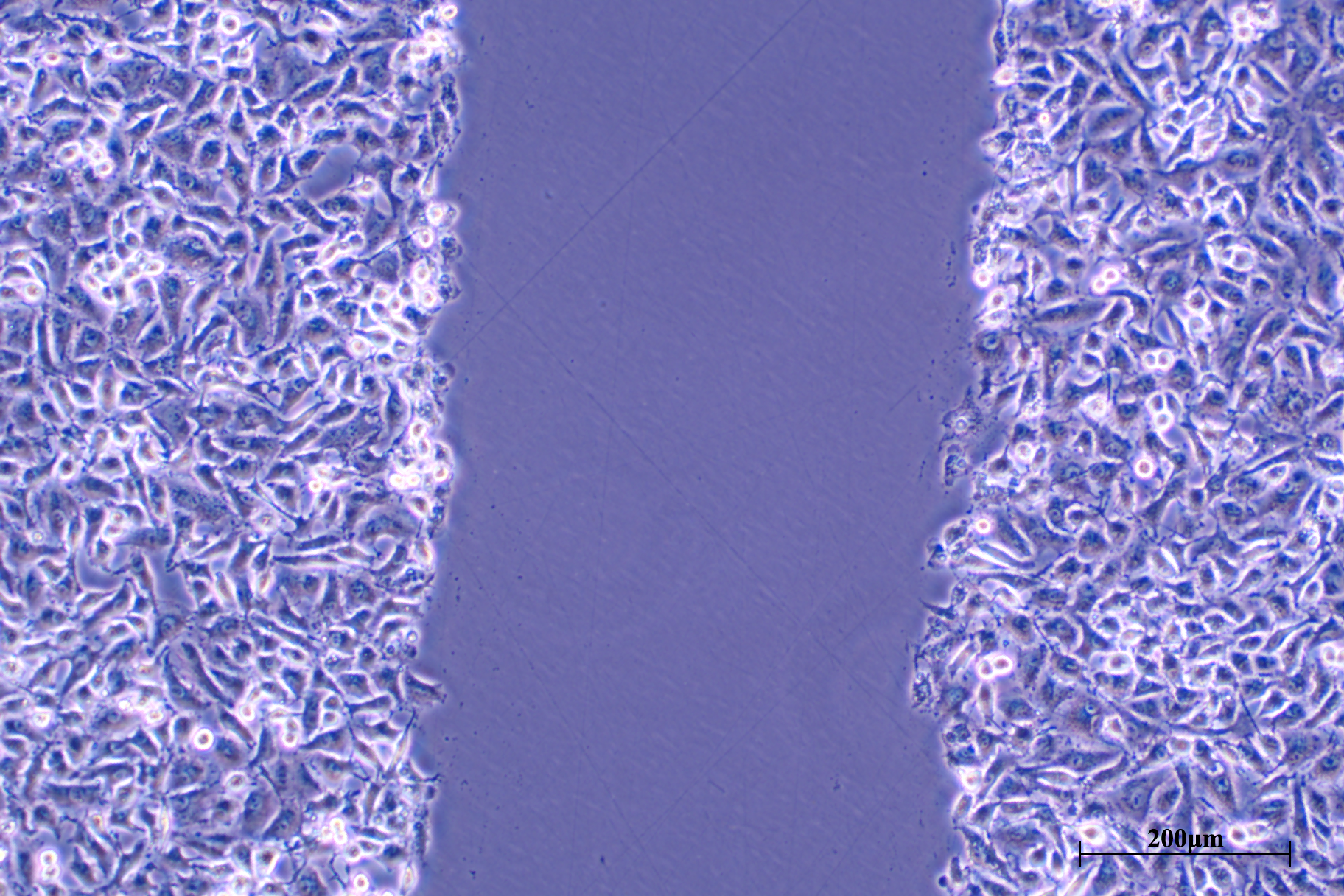

Supplement: Supplementary file 1 — Supplementary Information 1. [file 41598_2024_59725_MOESM1_ESM.zip › Original diagram of the cell experiment/fig3C/0 HOUR/0009.tif]

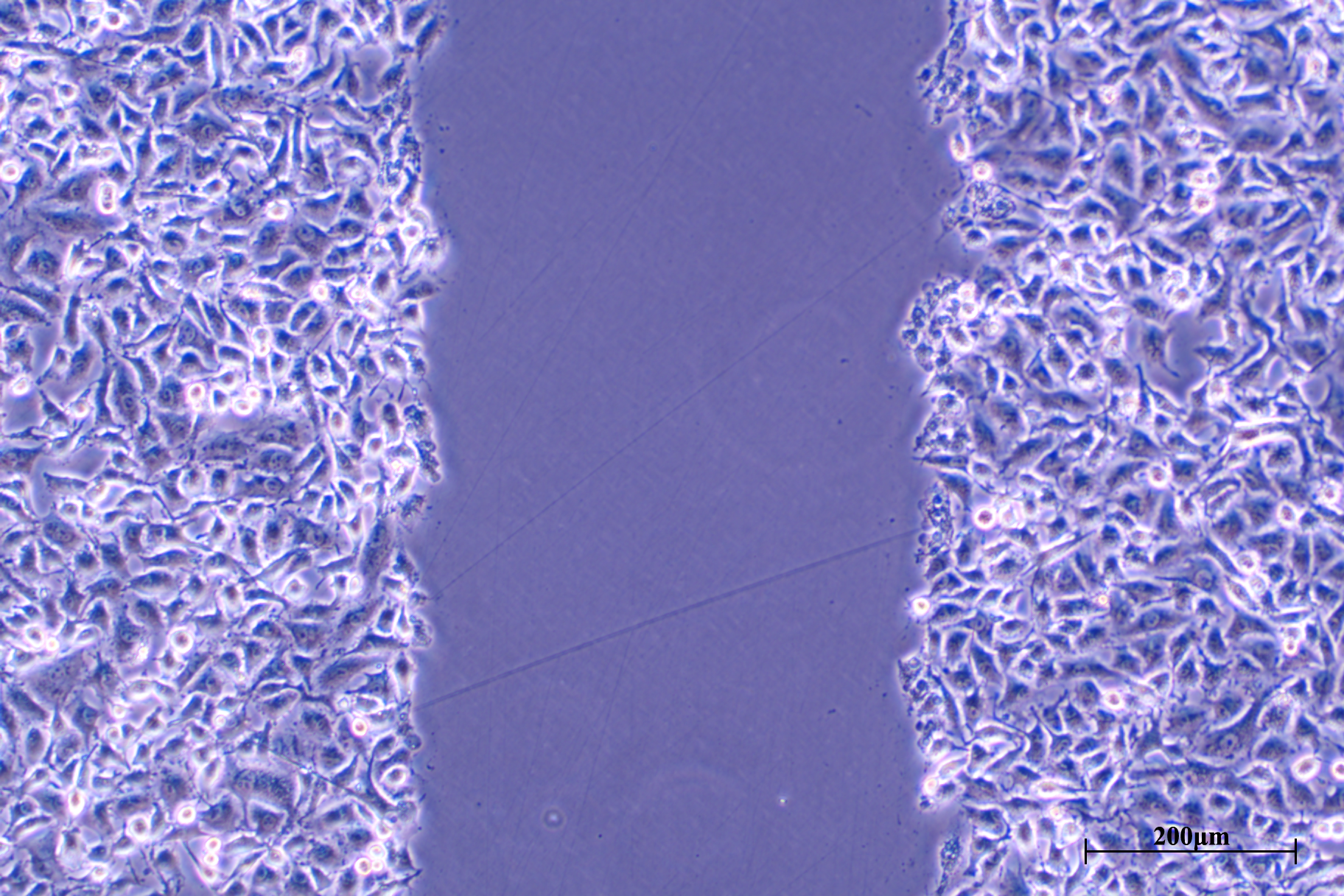

Supplement: Supplementary file 1 — Supplementary Information 1. [file 41598_2024_59725_MOESM1_ESM.zip › Original diagram of the cell experiment/fig3C/0 HOUR/0010.tif]

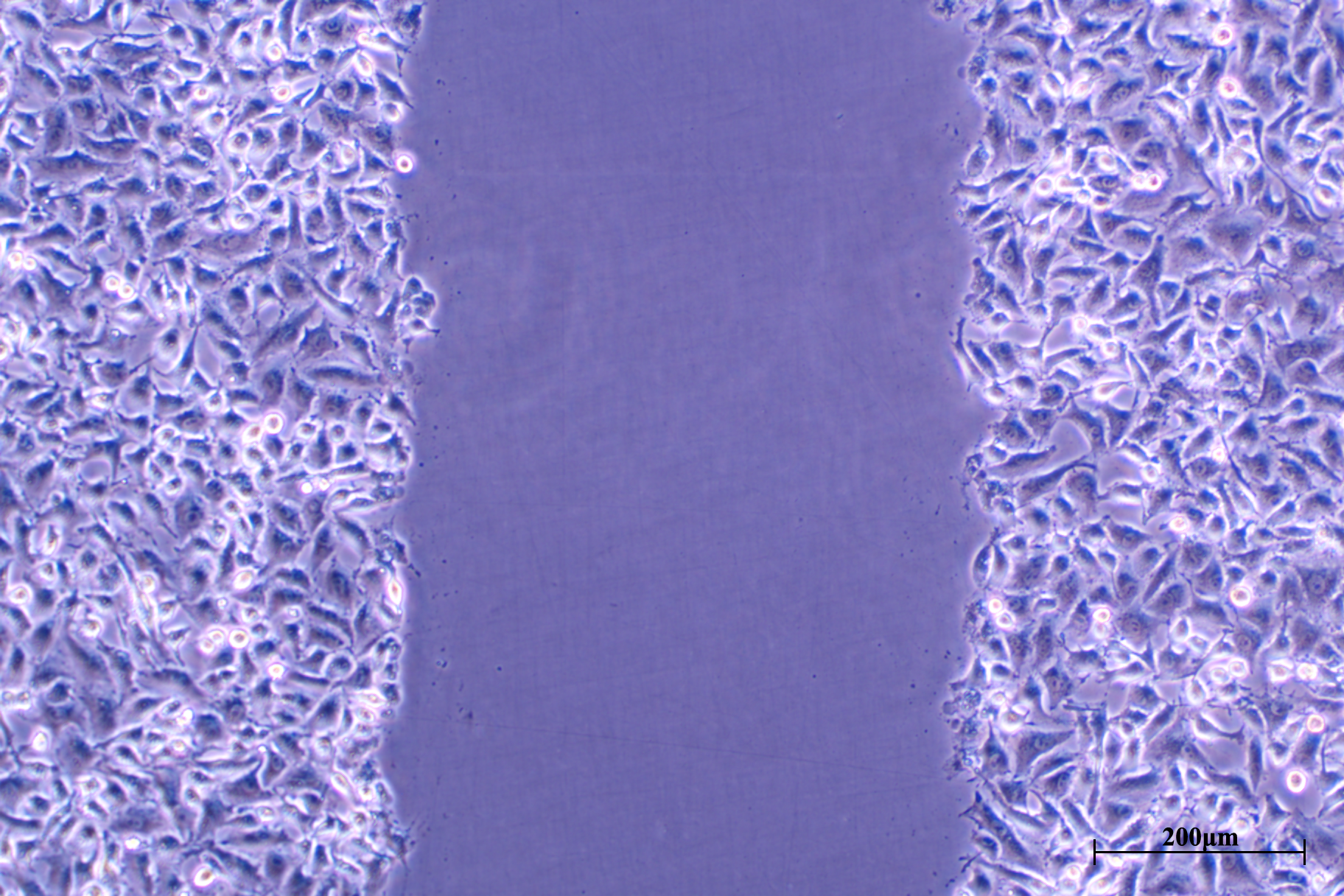

Supplement: Supplementary file 1 — Supplementary Information 1. [file 41598_2024_59725_MOESM1_ESM.zip › Original diagram of the cell experiment/fig3C/0 HOUR/0011.tif]

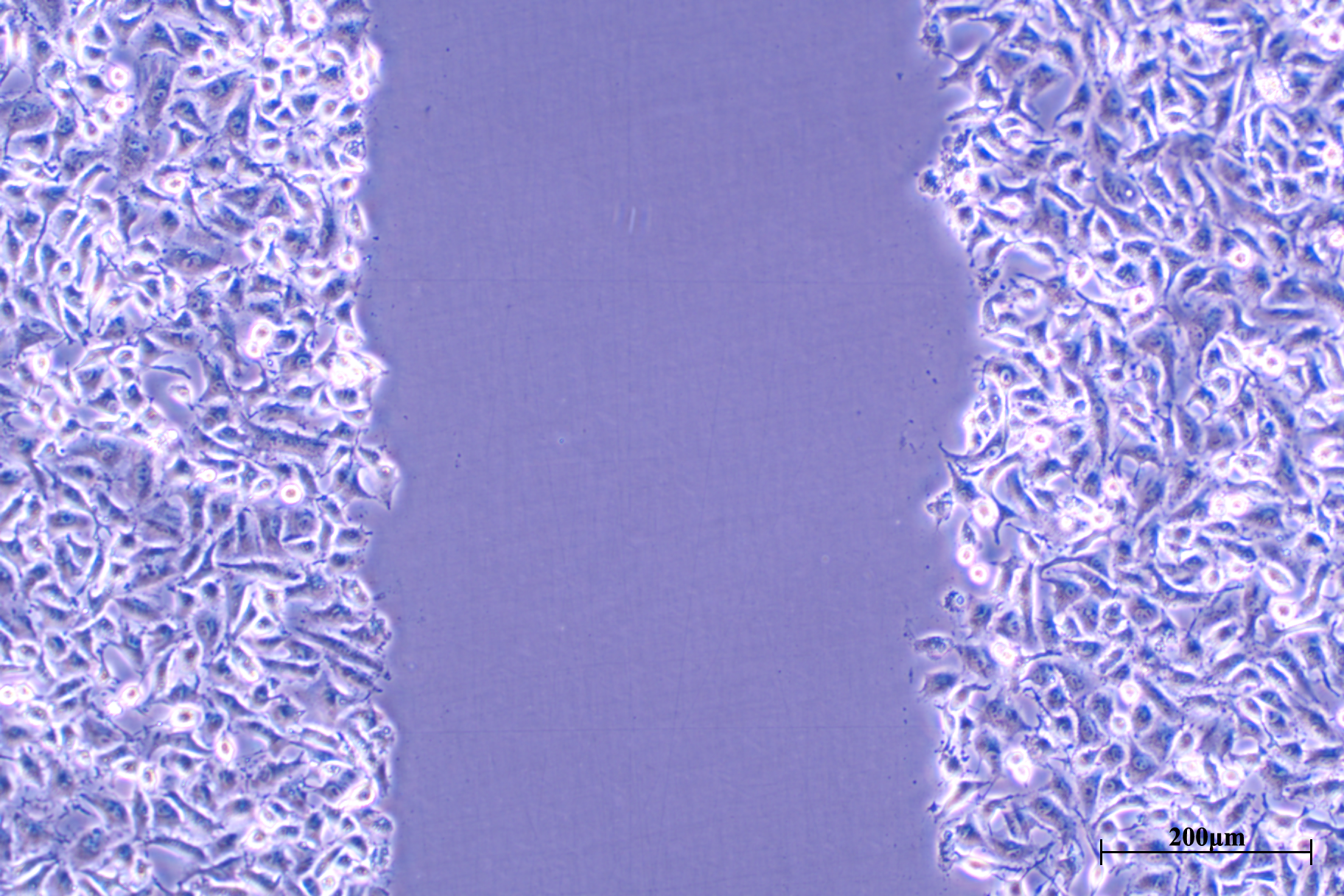

Supplement: Supplementary file 1 — Supplementary Information 1. [file 41598_2024_59725_MOESM1_ESM.zip › Original diagram of the cell experiment/fig3C/0 HOUR/0012.tif]

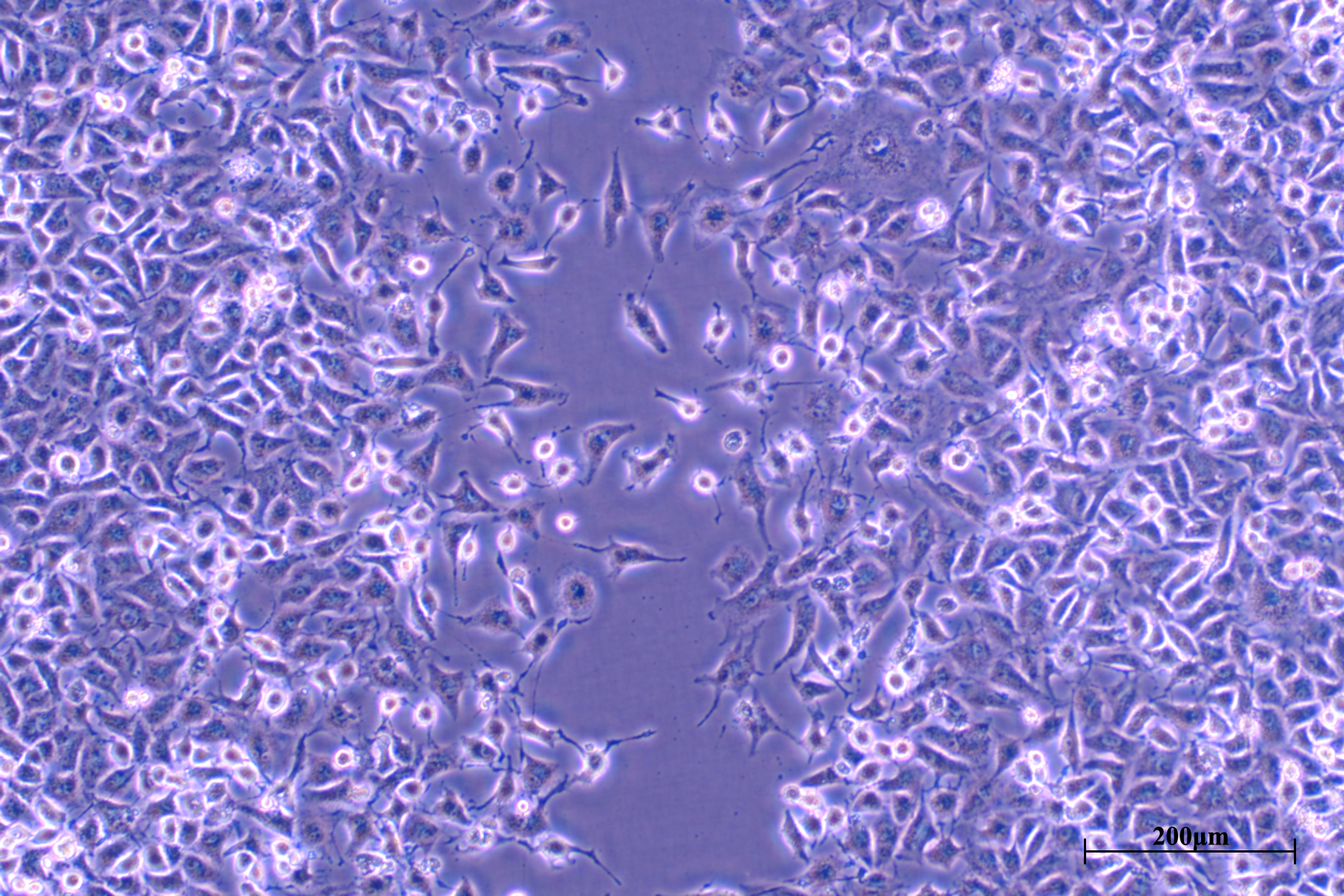

Supplement: Supplementary file 1 — Supplementary Information 1. [file 41598_2024_59725_MOESM1_ESM.zip › Original diagram of the cell experiment/fig3C/48 HOUR/1.tif]

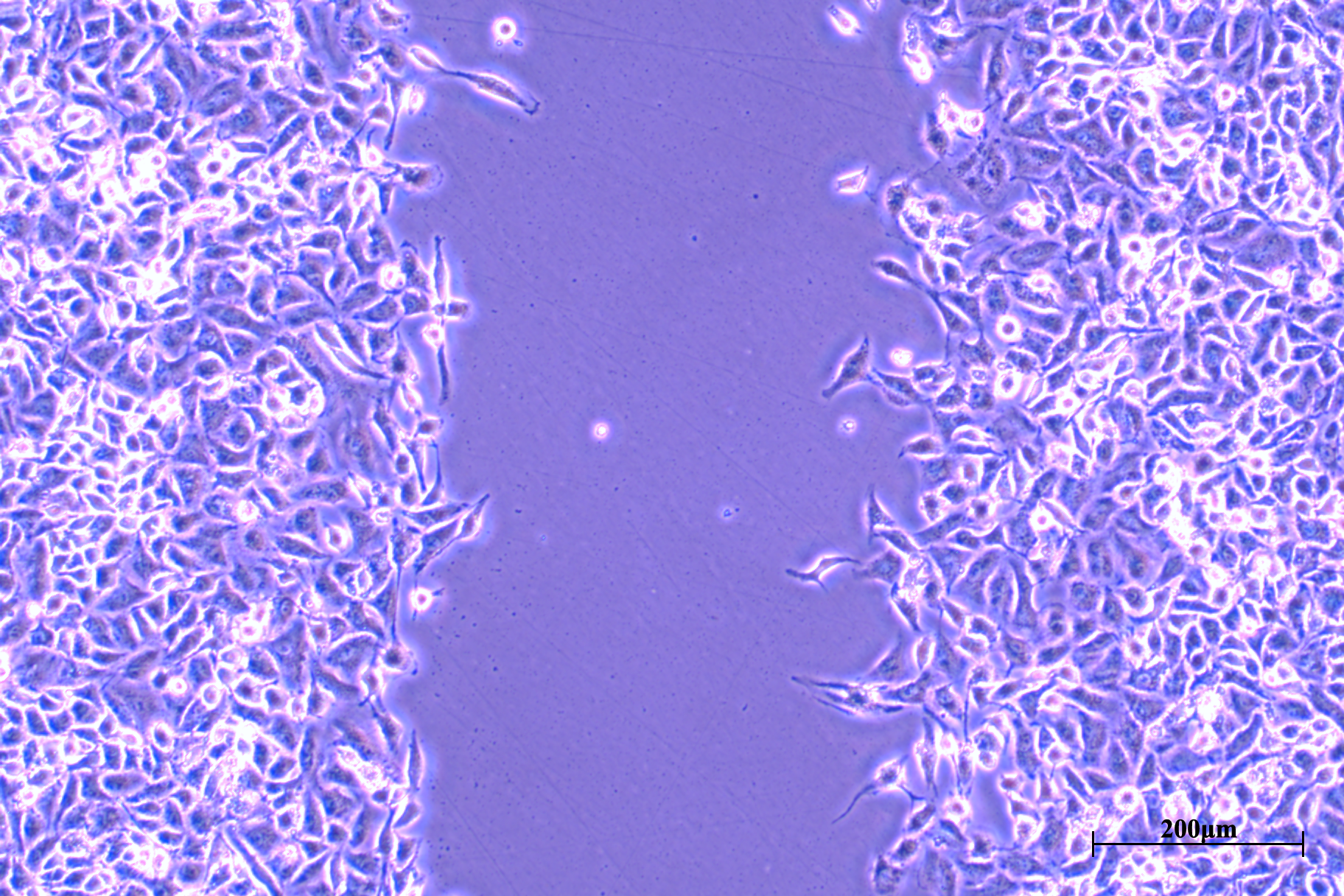

Supplement: Supplementary file 1 — Supplementary Information 1. [file 41598_2024_59725_MOESM1_ESM.zip › Original diagram of the cell experiment/fig3C/48 HOUR/2.tif]

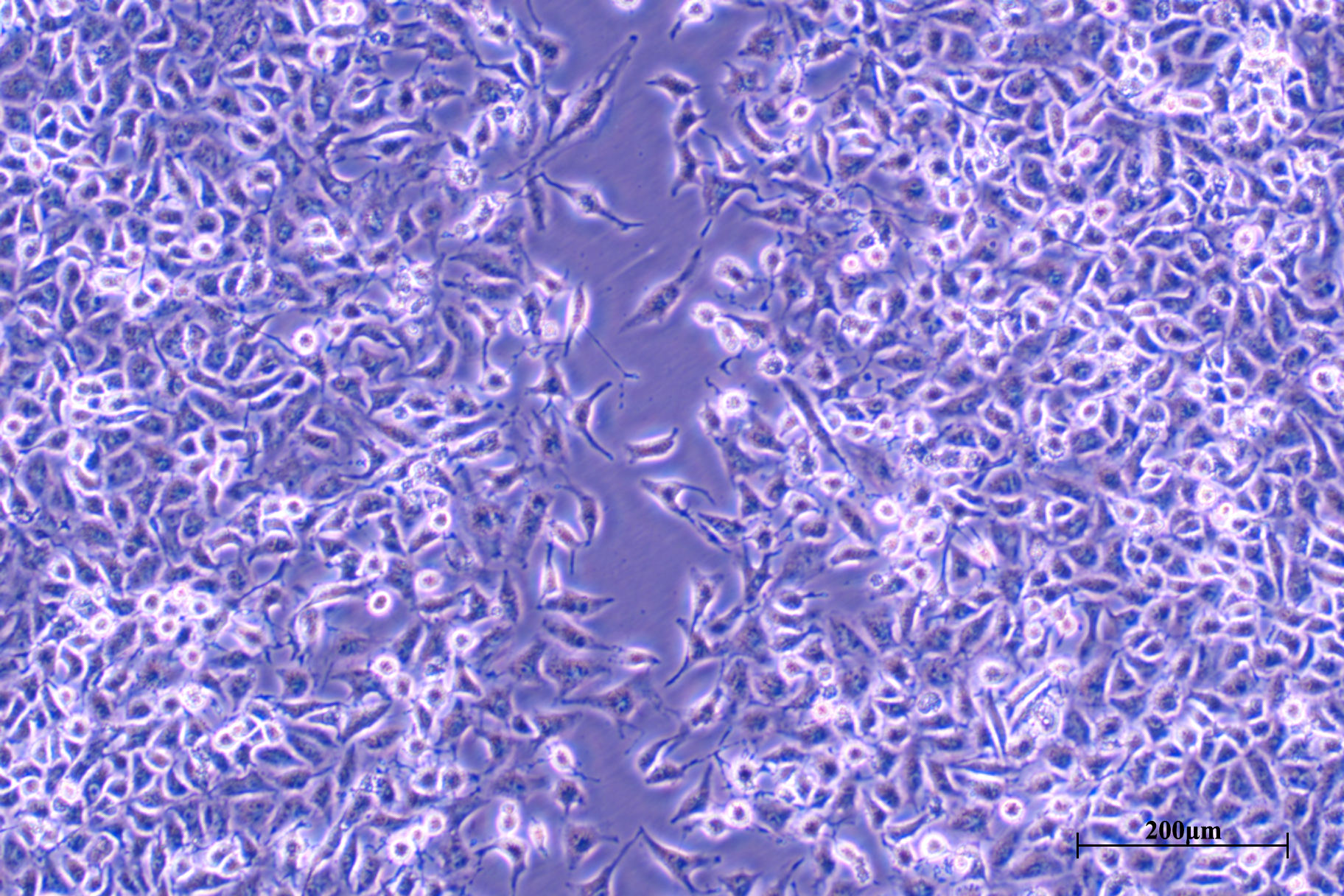

Supplement: Supplementary file 1 — Supplementary Information 1. [file 41598_2024_59725_MOESM1_ESM.zip › Original diagram of the cell experiment/fig3C/48 HOUR/3.tif]

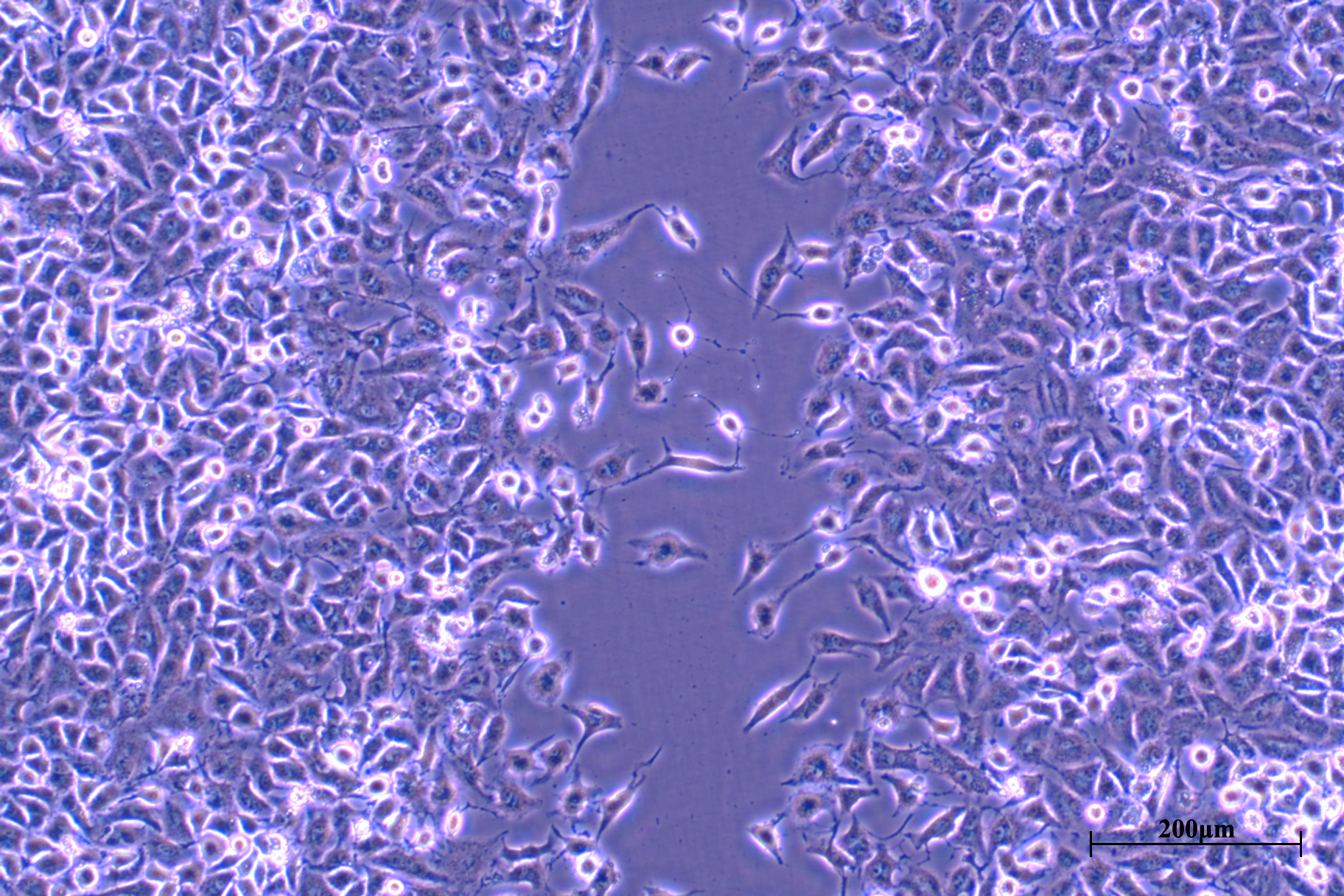

Supplement: Supplementary file 1 — Supplementary Information 1. [file 41598_2024_59725_MOESM1_ESM.zip › Original diagram of the cell experiment/fig3C/48 HOUR/4.tif]

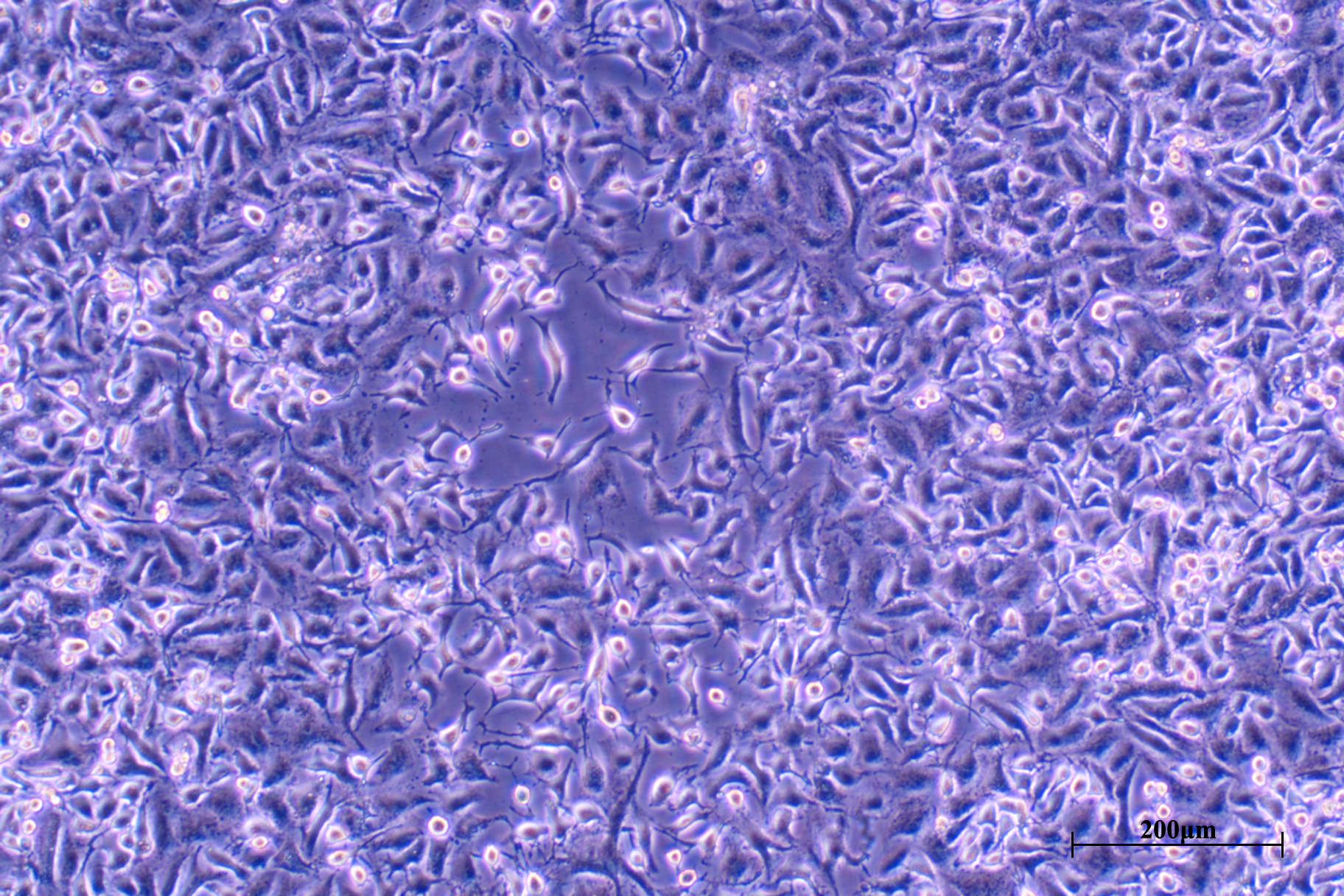

Supplement: Supplementary file 1 — Supplementary Information 1. [file 41598_2024_59725_MOESM1_ESM.zip › Original diagram of the cell experiment/fig3C/48 HOUR/5.tif]

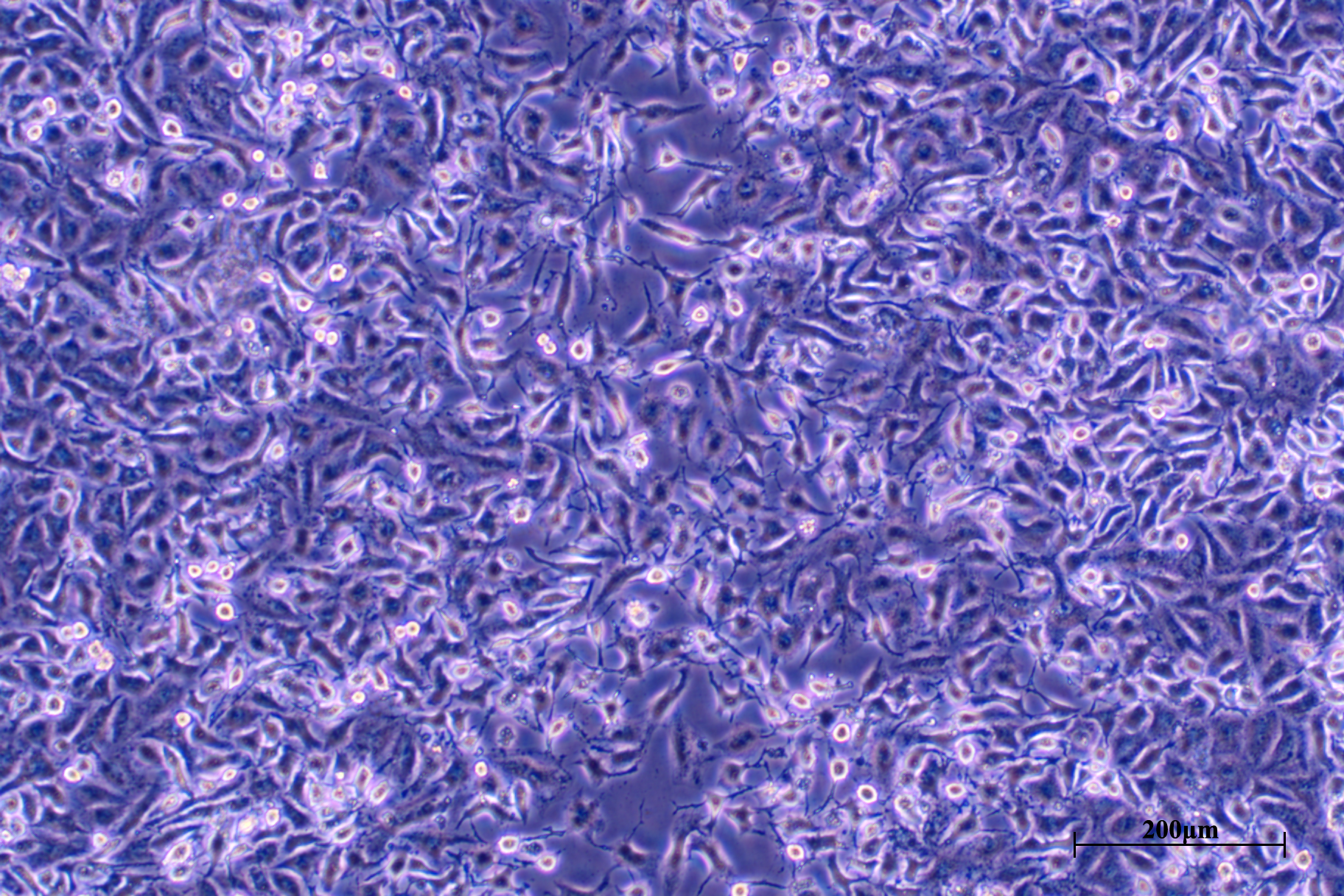

Supplement: Supplementary file 1 — Supplementary Information 1. [file 41598_2024_59725_MOESM1_ESM.zip › Original diagram of the cell experiment/fig3C/48 HOUR/6.tif]

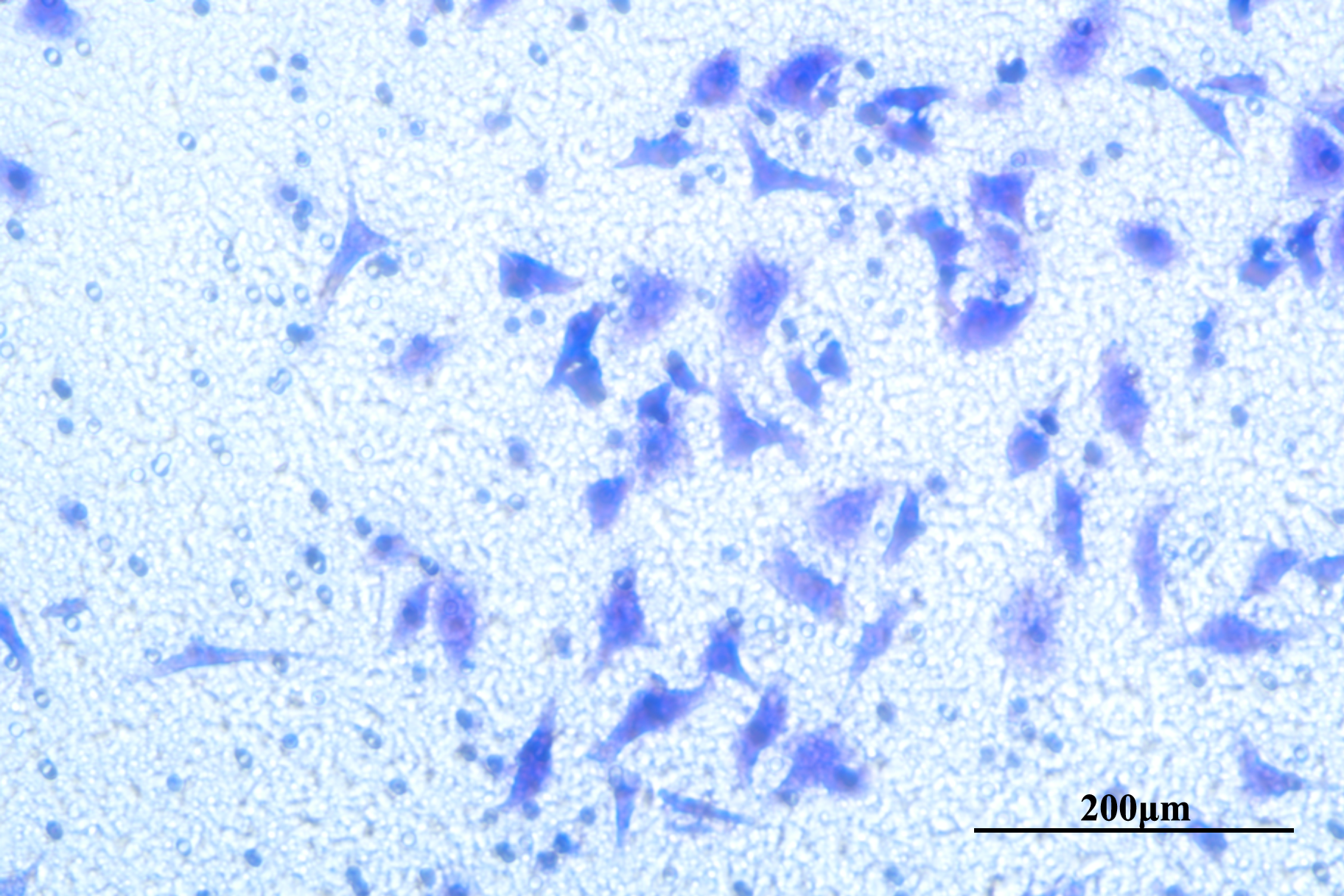

Supplement: Supplementary file 1 — Supplementary Information 1. [file 41598_2024_59725_MOESM1_ESM.zip › Original diagram of the cell experiment/fig3D/1.tif]

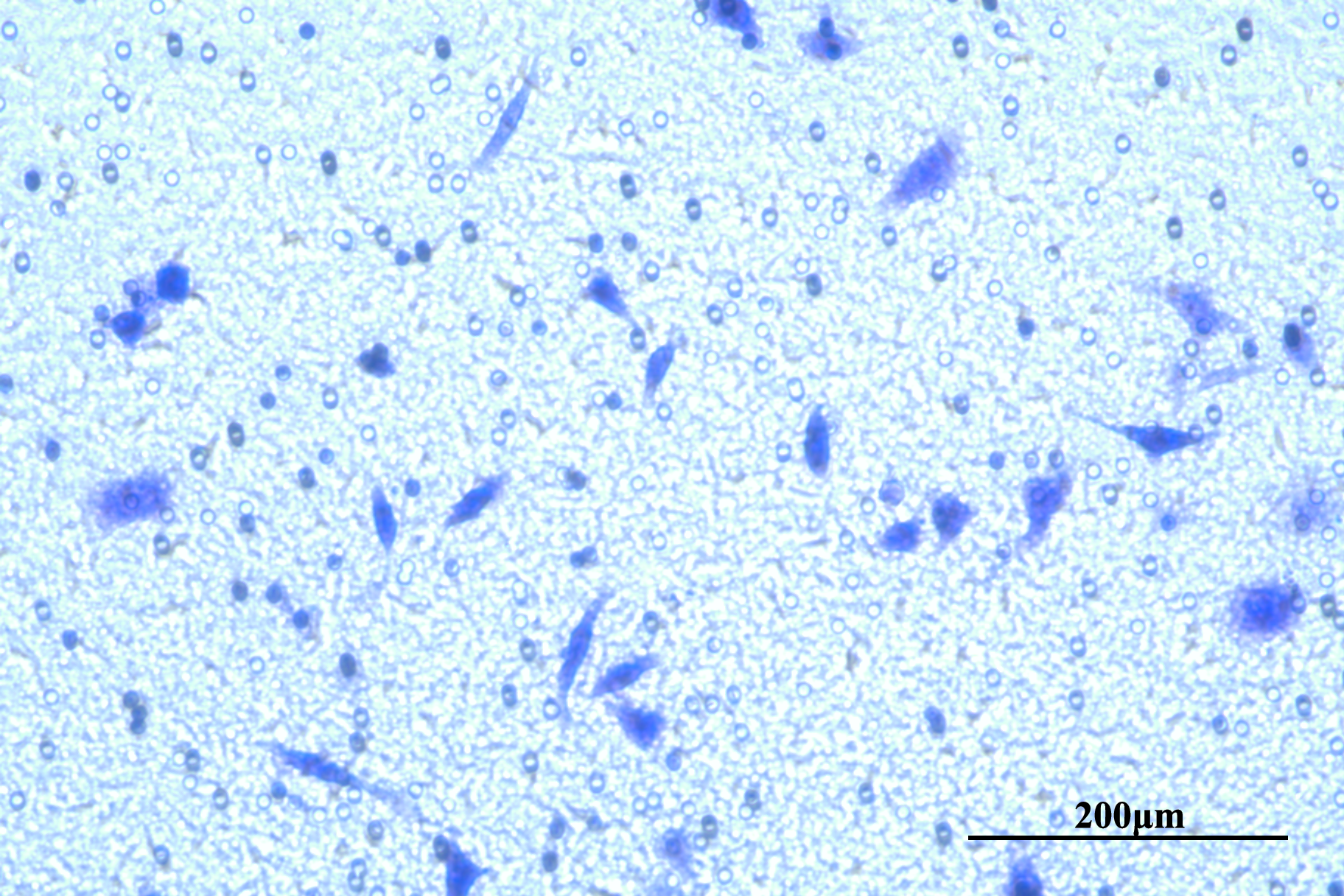

Supplement: Supplementary file 1 — Supplementary Information 1. [file 41598_2024_59725_MOESM1_ESM.zip › Original diagram of the cell experiment/fig3D/2.tif]

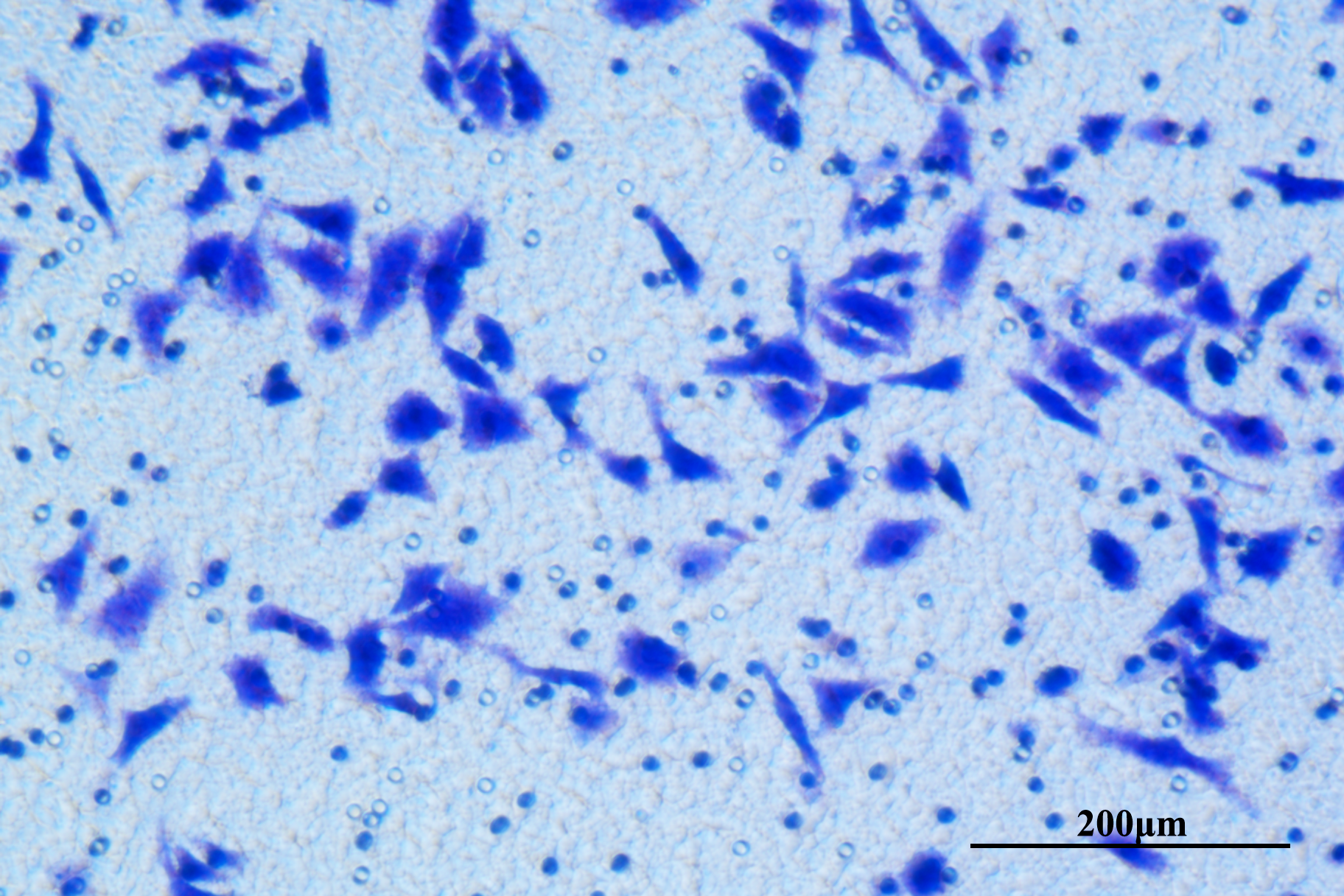

Supplement: Supplementary file 1 — Supplementary Information 1. [file 41598_2024_59725_MOESM1_ESM.zip › Original diagram of the cell experiment/fig3D/3.tif]

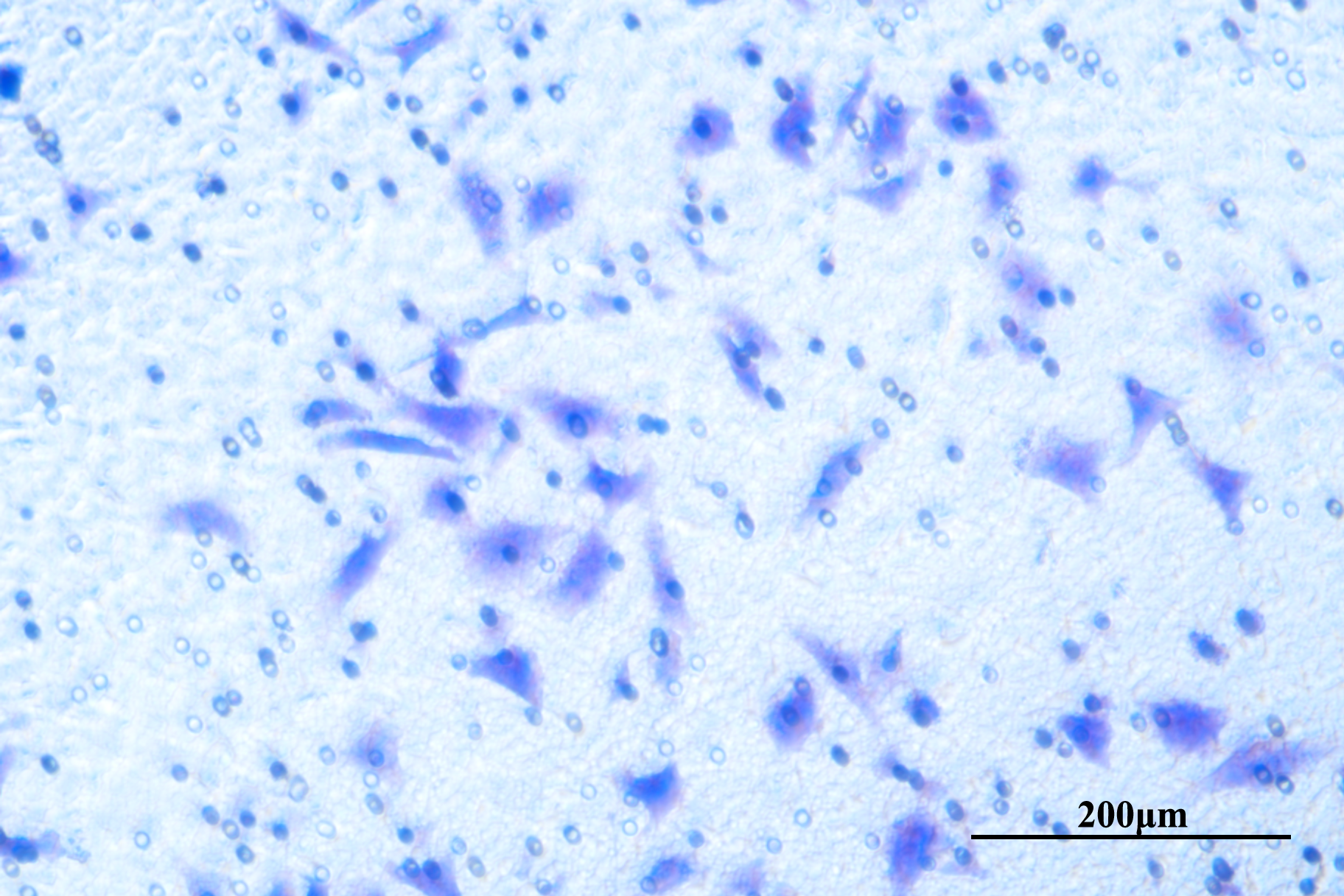

Supplement: Supplementary file 1 — Supplementary Information 1. [file 41598_2024_59725_MOESM1_ESM.zip › Original diagram of the cell experiment/fig3D/4.tif]

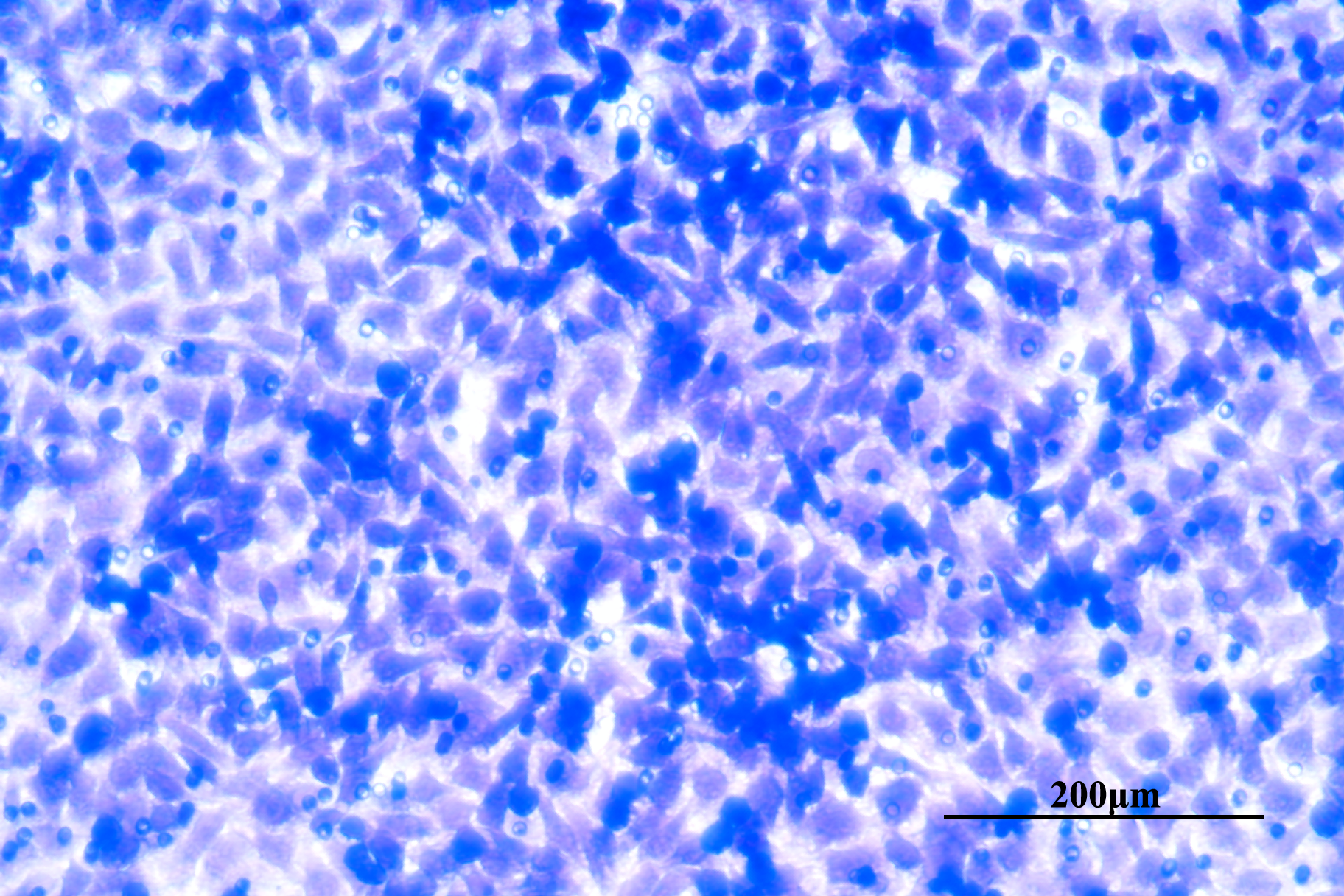

Supplement: Supplementary file 1 — Supplementary Information 1. [file 41598_2024_59725_MOESM1_ESM.zip › Original diagram of the cell experiment/fig3D/5.tif]

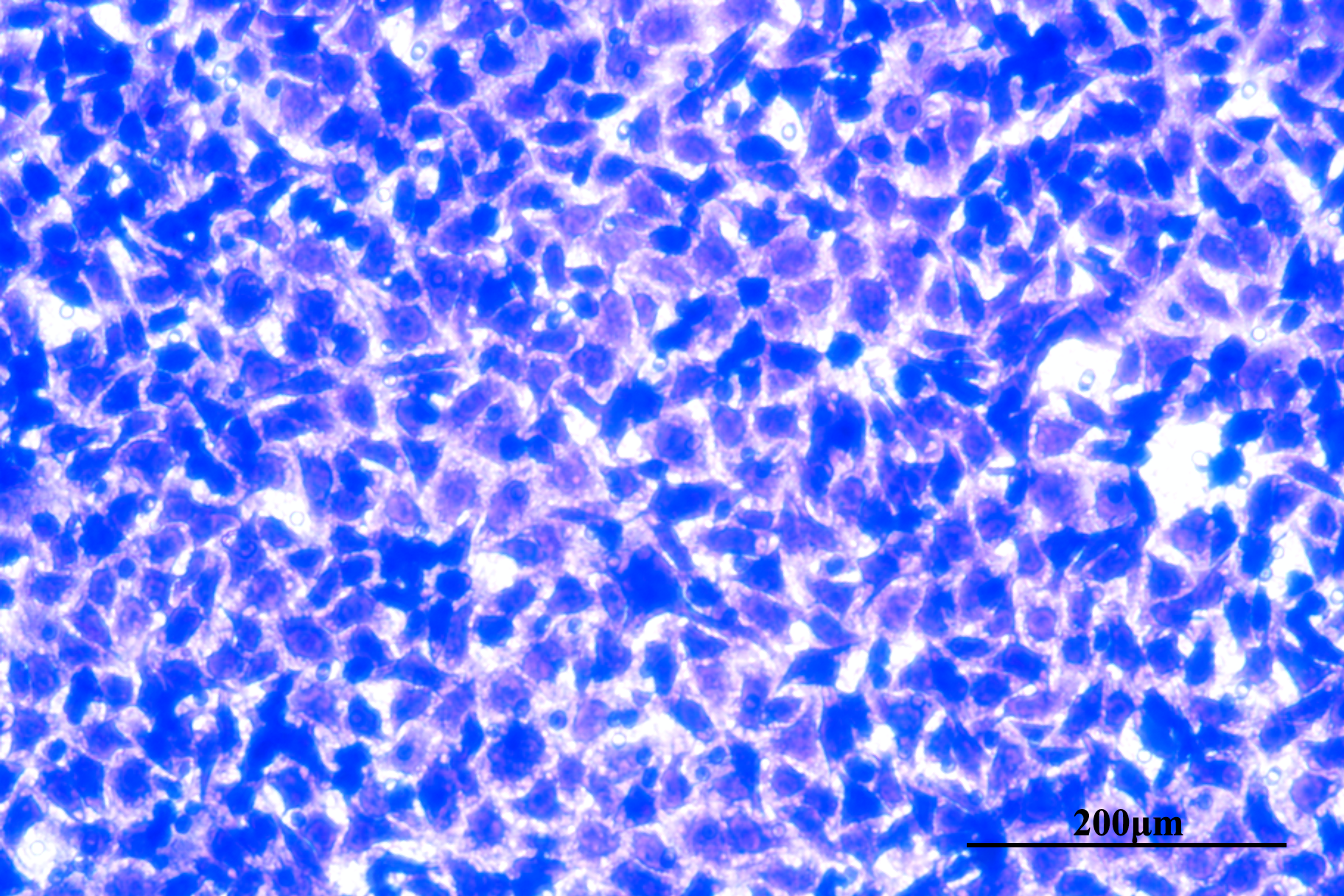

Supplement: Supplementary file 1 — Supplementary Information 1. [file 41598_2024_59725_MOESM1_ESM.zip › Original diagram of the cell experiment/fig3D/6.tif]

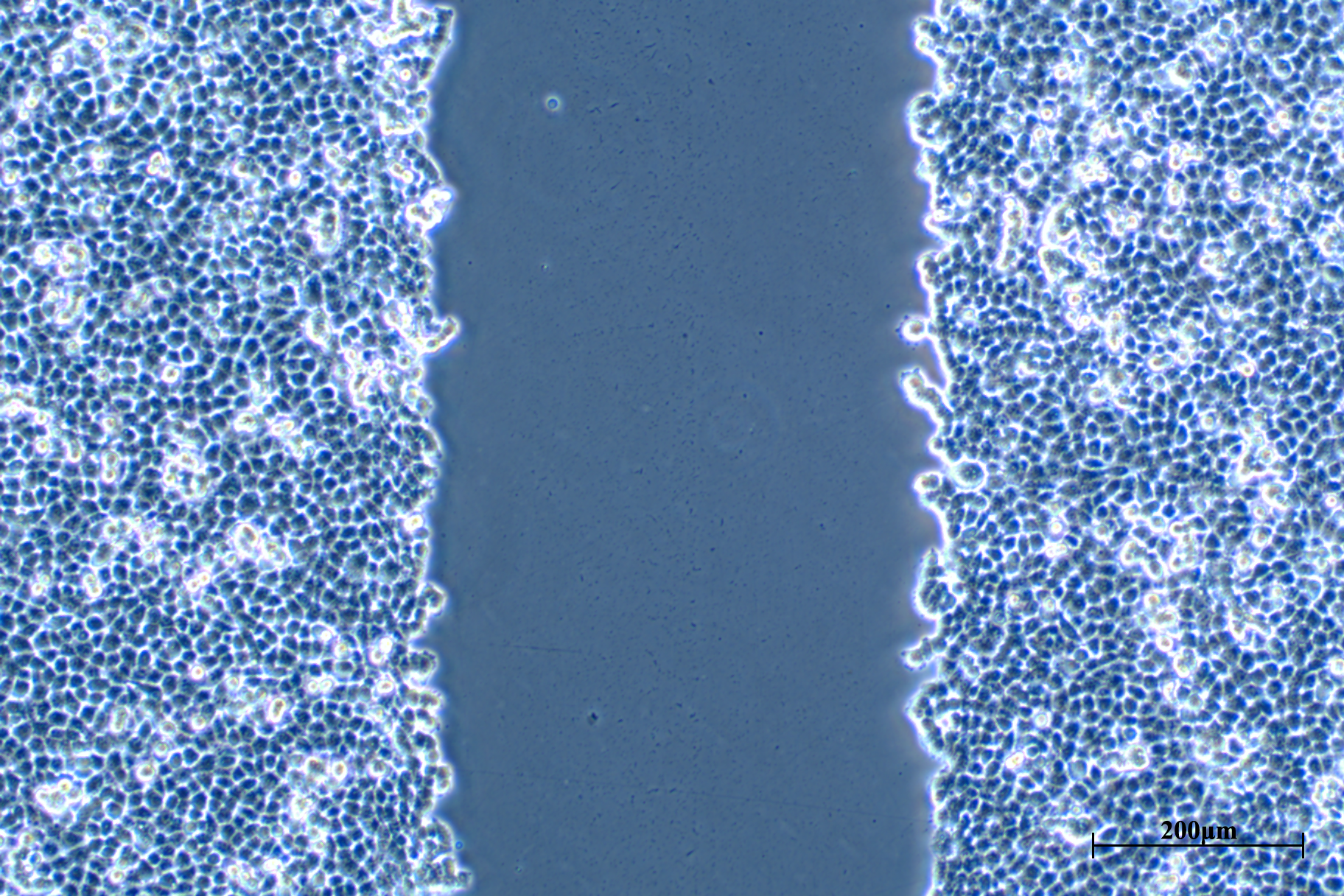

Supplement: Supplementary file 1 — Supplementary Information 1. [file 41598_2024_59725_MOESM1_ESM.zip › Original diagram of the cell experiment/fig3E/0 HOUR/0╨í╩▒ (2).tif]

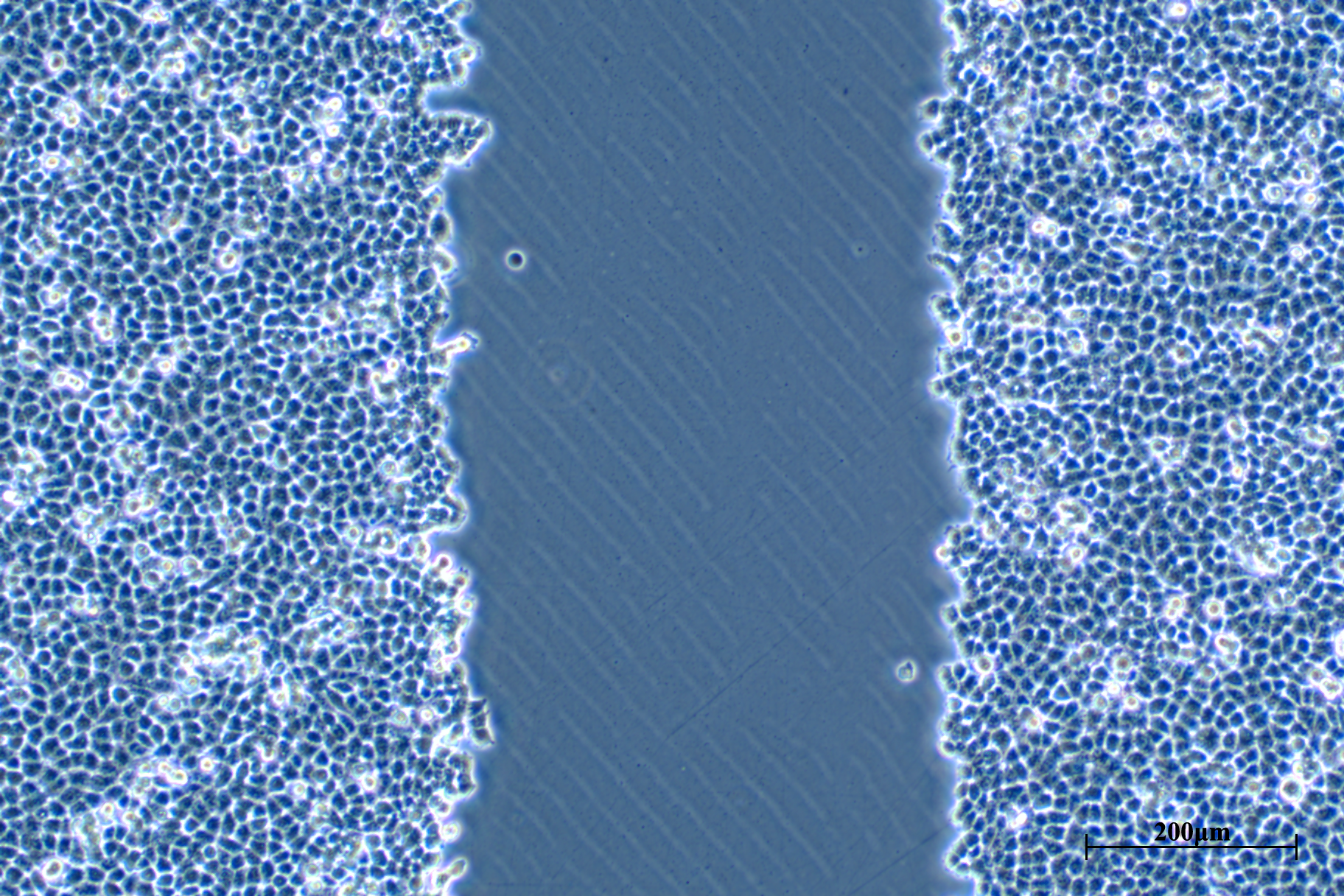

Supplement: Supplementary file 1 — Supplementary Information 1. [file 41598_2024_59725_MOESM1_ESM.zip › Original diagram of the cell experiment/fig3E/0 HOUR/0╨í╩▒ (3).tif]

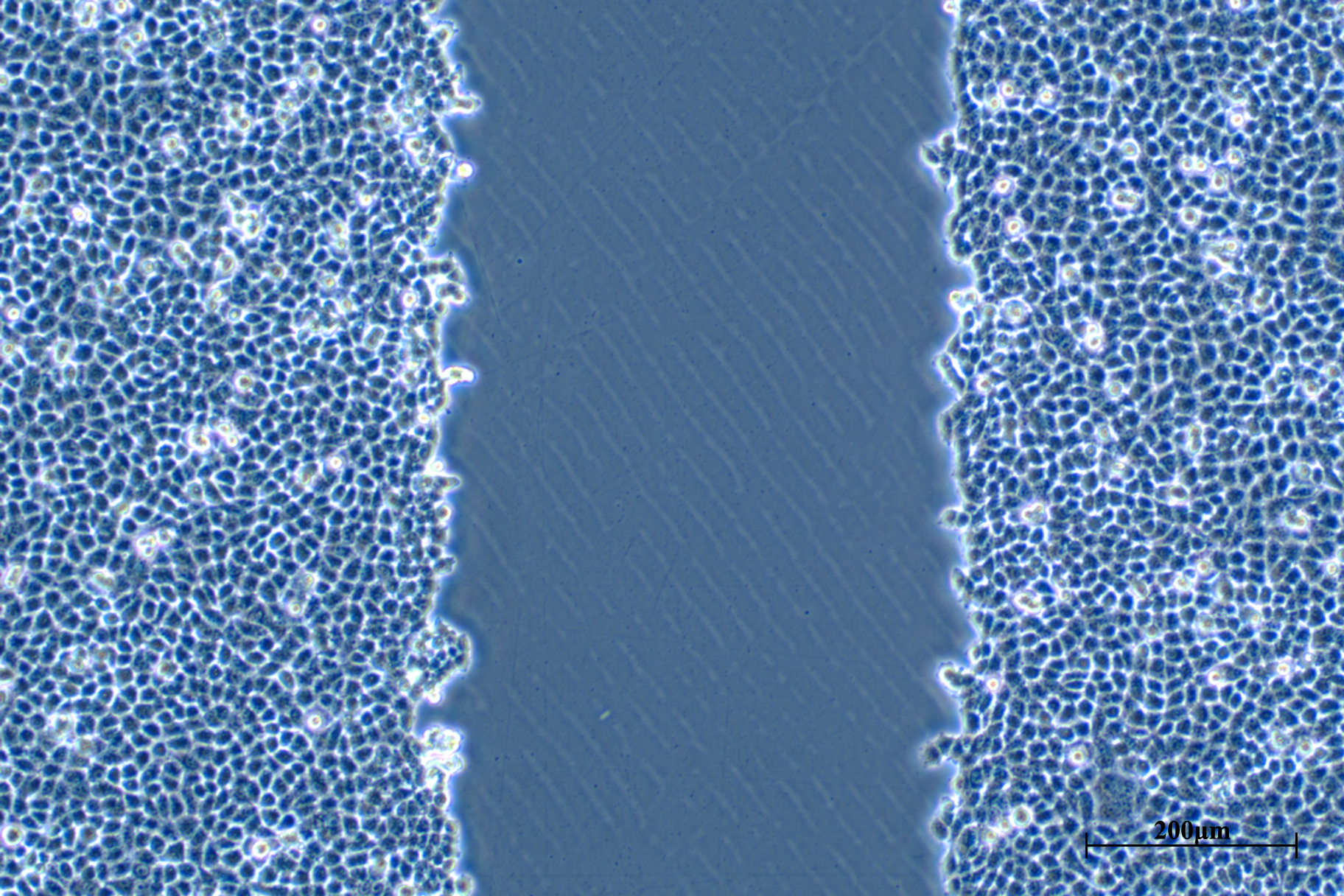

Supplement: Supplementary file 1 — Supplementary Information 1. [file 41598_2024_59725_MOESM1_ESM.zip › Original diagram of the cell experiment/fig3E/0 HOUR/0╨í╩▒ (4).tif]

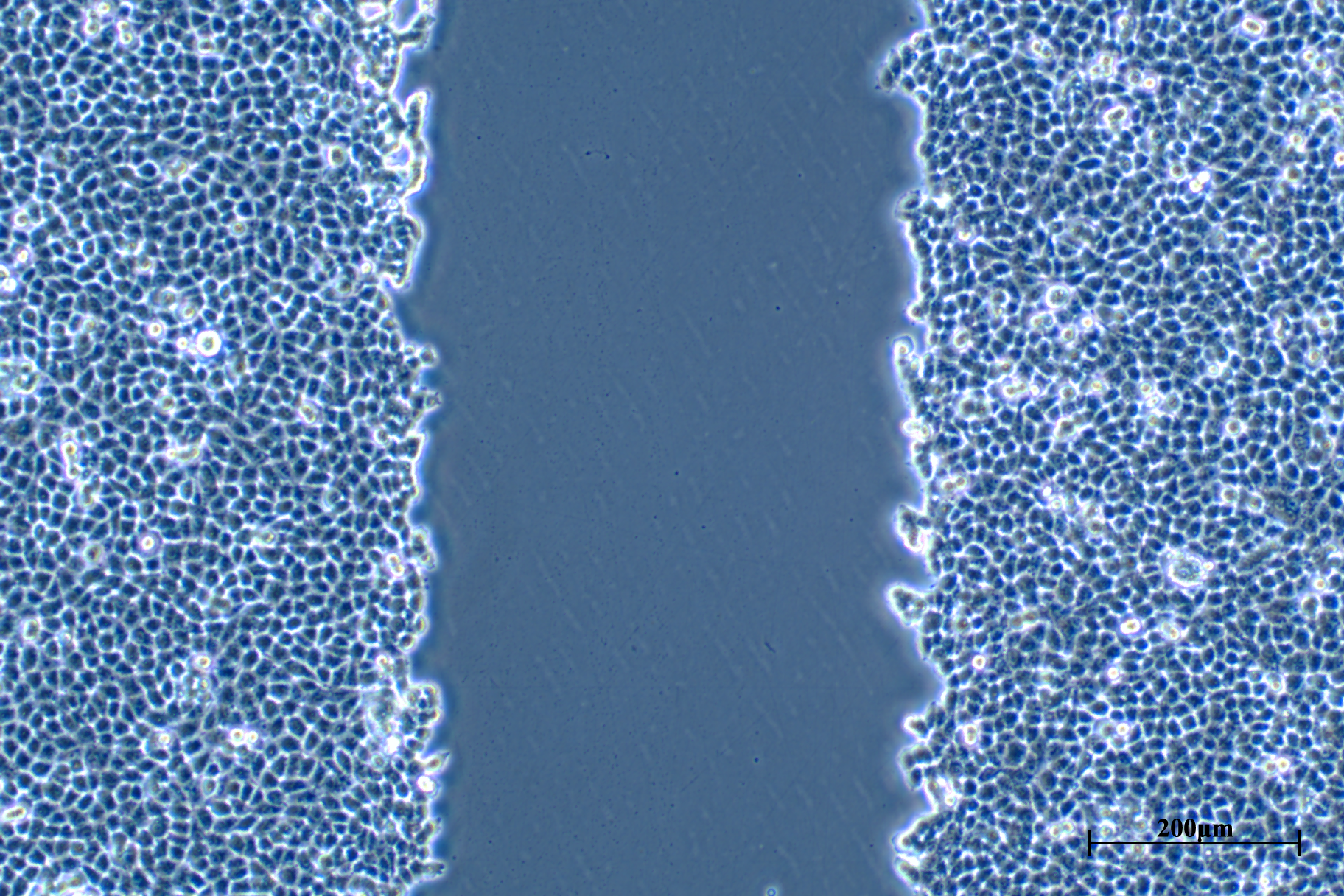

Supplement: Supplementary file 1 — Supplementary Information 1. [file 41598_2024_59725_MOESM1_ESM.zip › Original diagram of the cell experiment/fig3E/0 HOUR/0╨í╩▒ (5).tif]

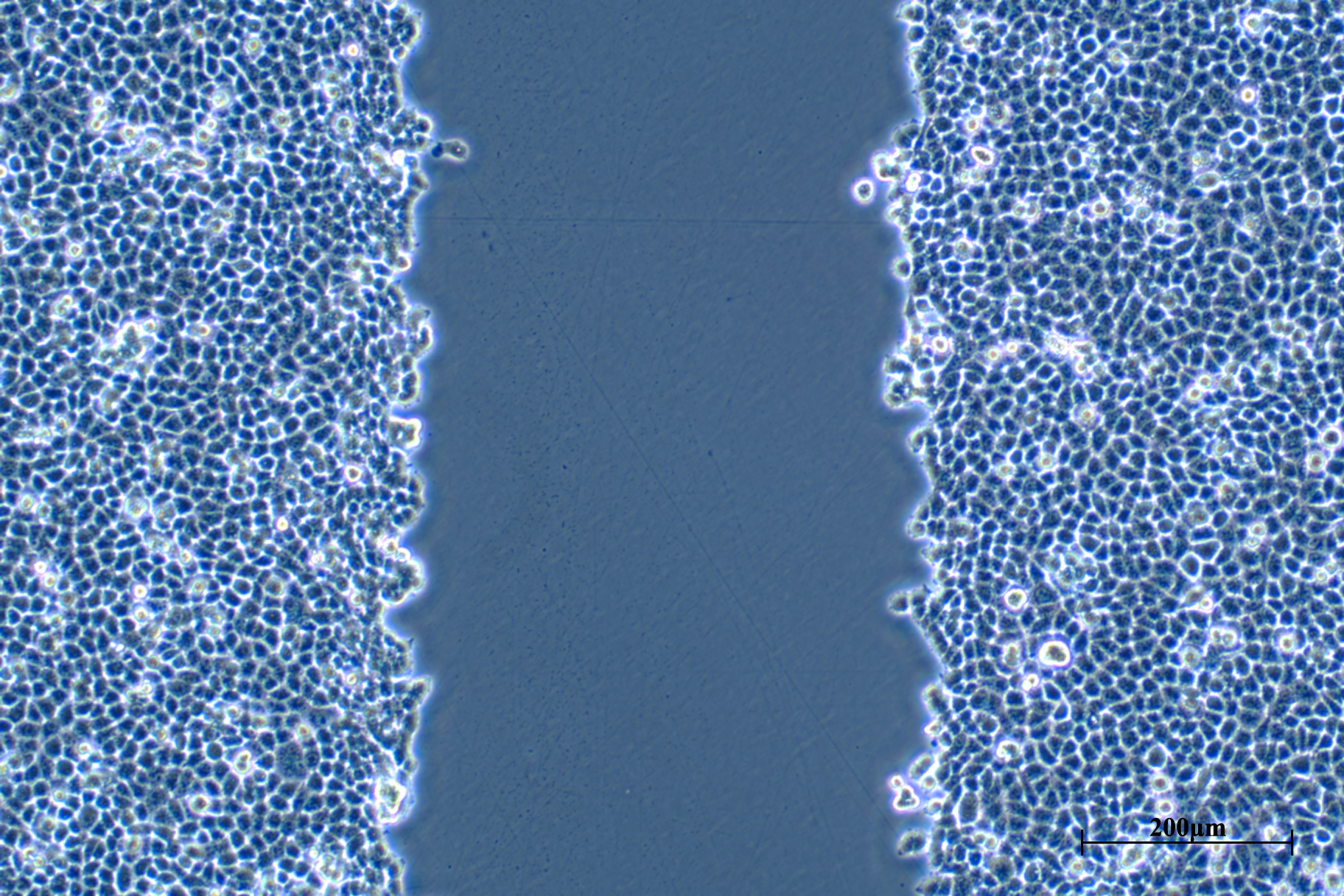

Supplement: Supplementary file 1 — Supplementary Information 1. [file 41598_2024_59725_MOESM1_ESM.zip › Original diagram of the cell experiment/fig3E/0 HOUR/0╨í╩▒ (6).tif]

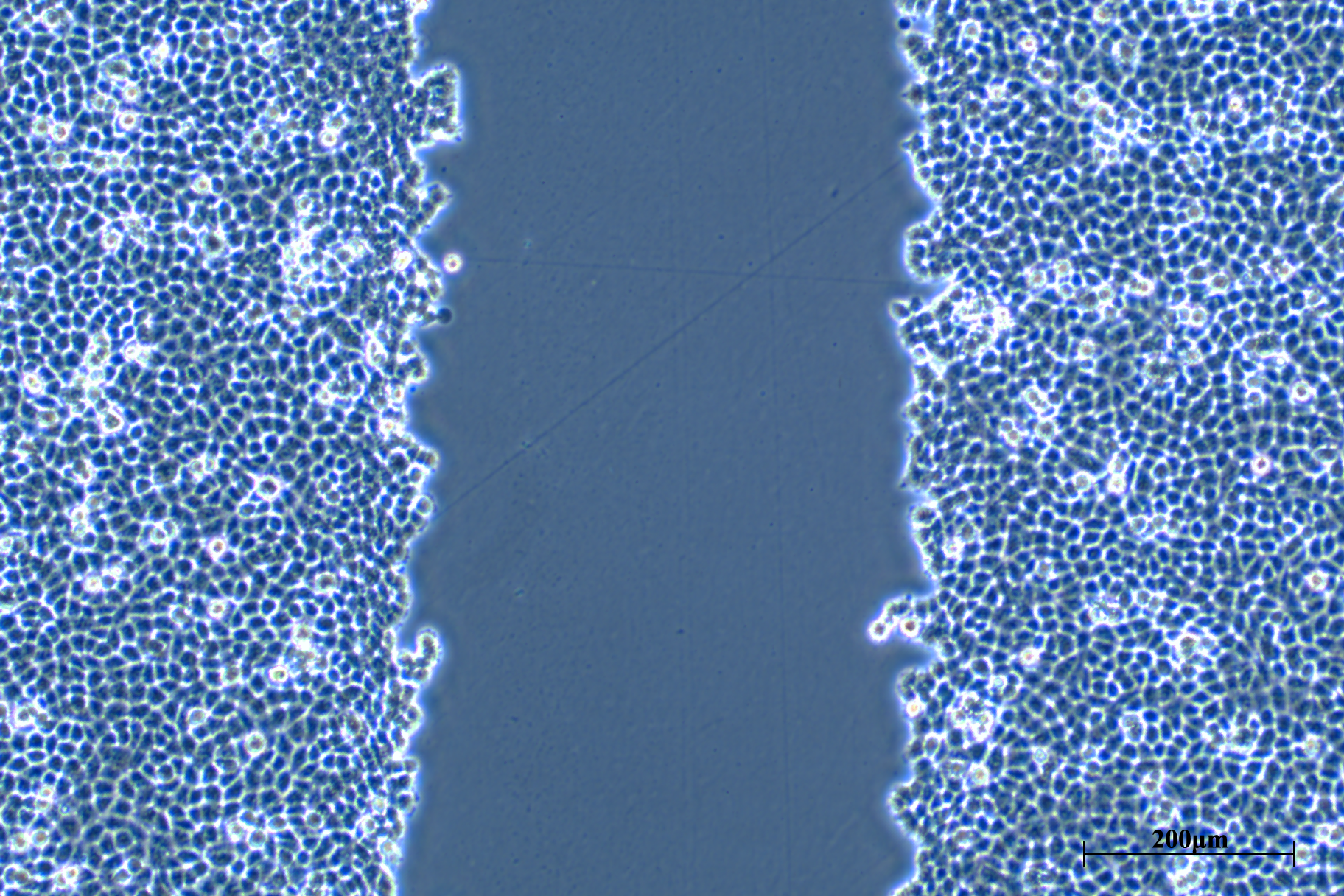

Supplement: Supplementary file 1 — Supplementary Information 1. [file 41598_2024_59725_MOESM1_ESM.zip › Original diagram of the cell experiment/fig3E/0 HOUR/0╨í╩▒ (7).tif]

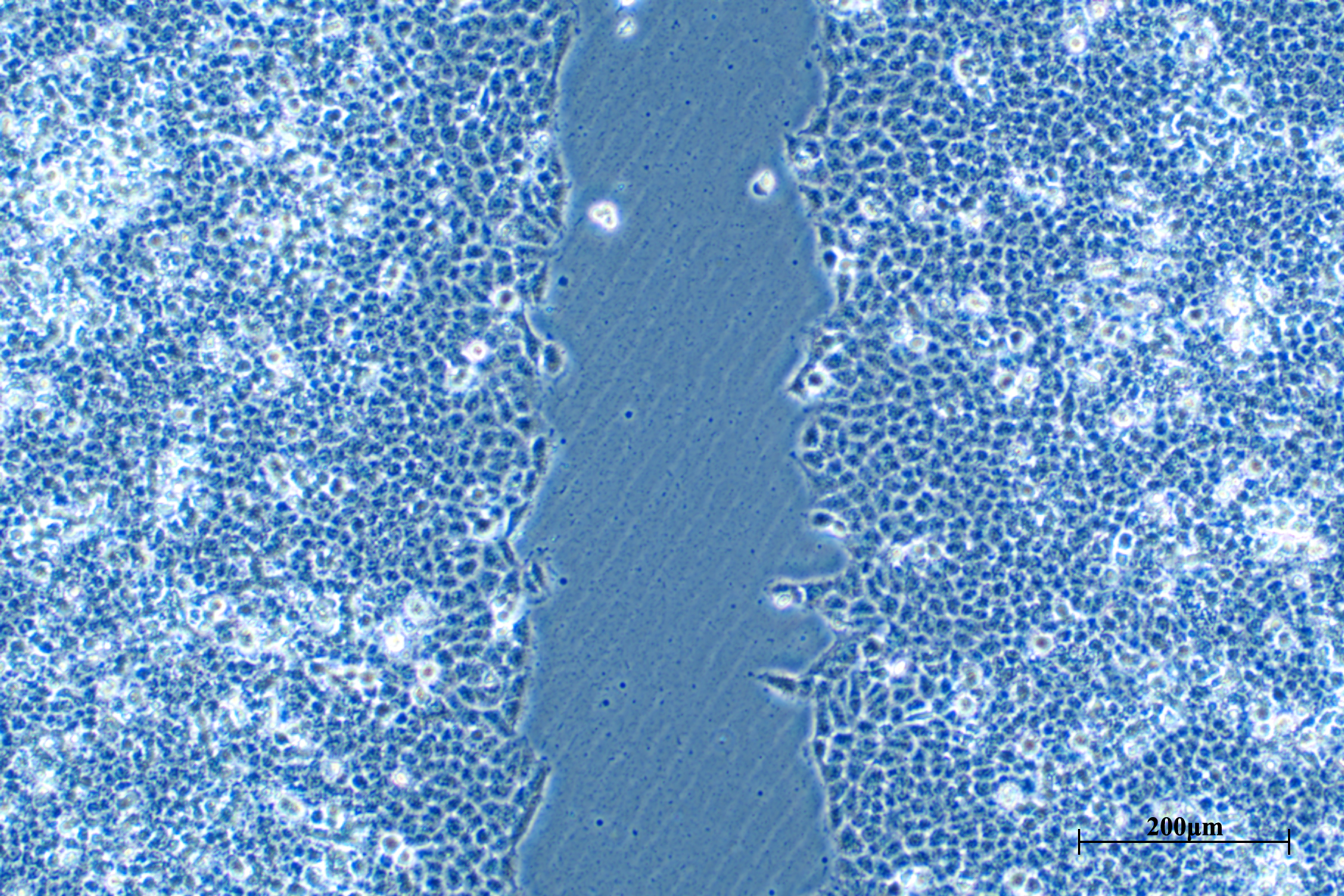

Supplement: Supplementary file 1 — Supplementary Information 1. [file 41598_2024_59725_MOESM1_ESM.zip › Original diagram of the cell experiment/fig3E/48 HOUR/1.tif]

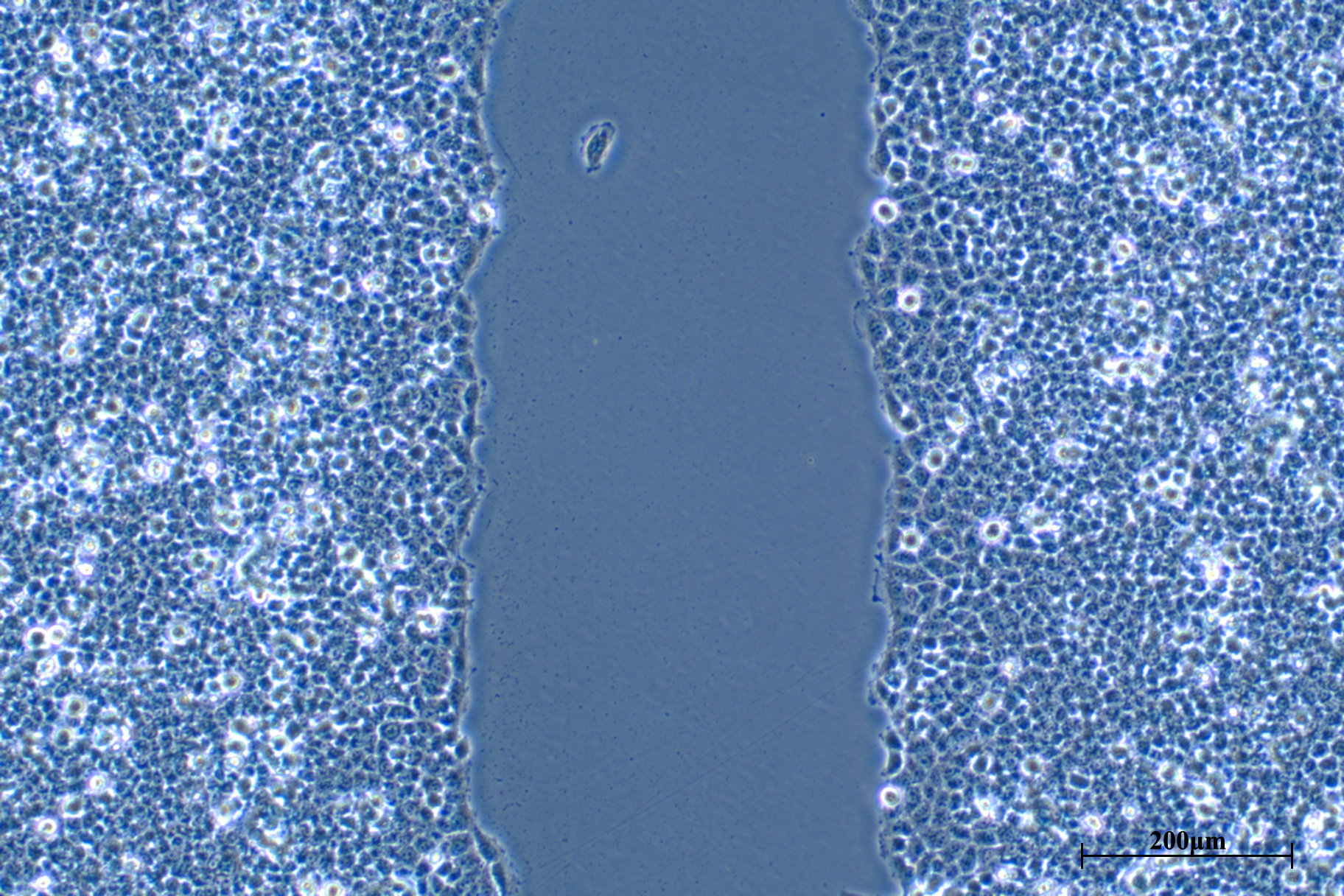

Supplement: Supplementary file 1 — Supplementary Information 1. [file 41598_2024_59725_MOESM1_ESM.zip › Original diagram of the cell experiment/fig3E/48 HOUR/2.tif]

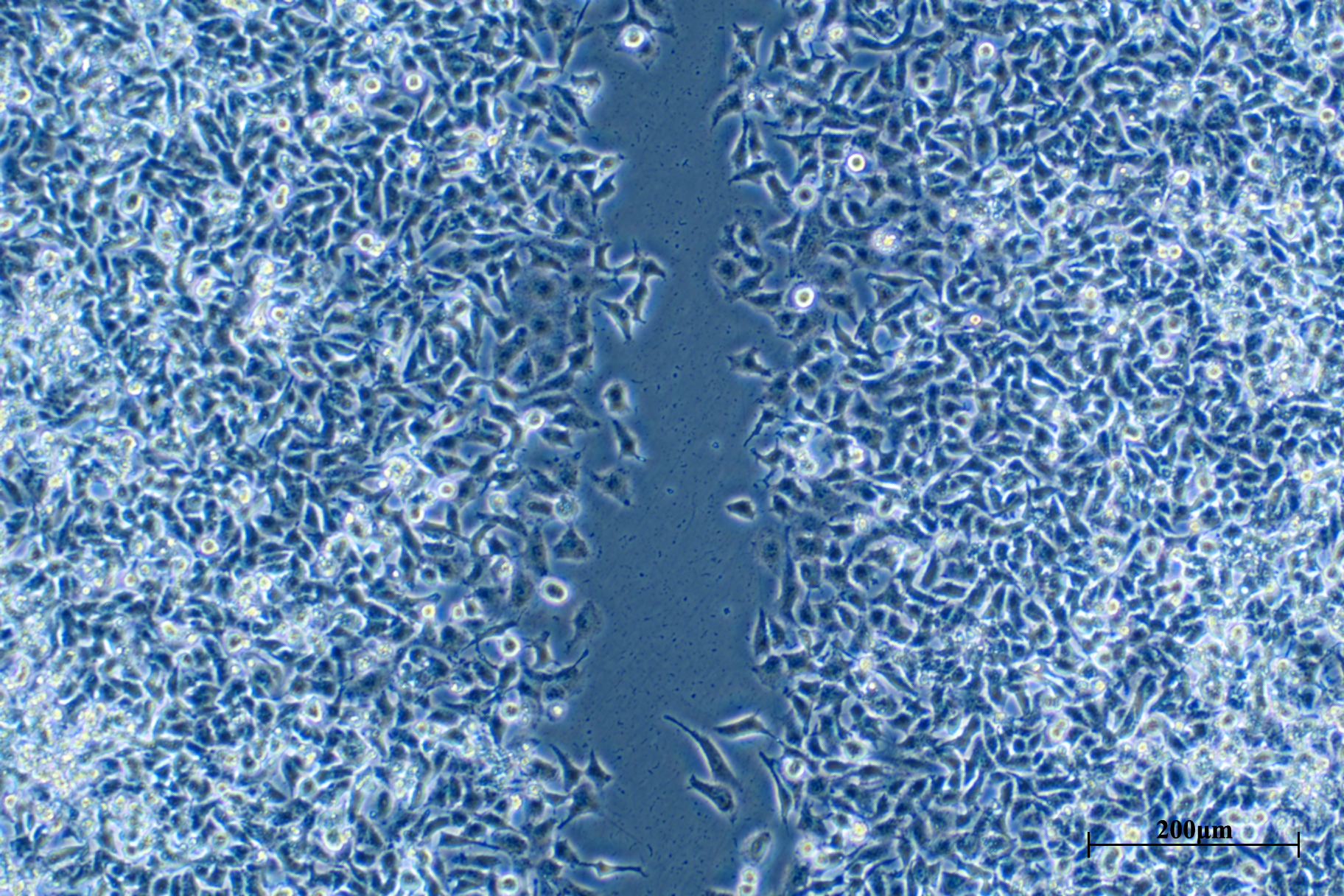

Supplement: Supplementary file 1 — Supplementary Information 1. [file 41598_2024_59725_MOESM1_ESM.zip › Original diagram of the cell experiment/fig3E/48 HOUR/3.tif]

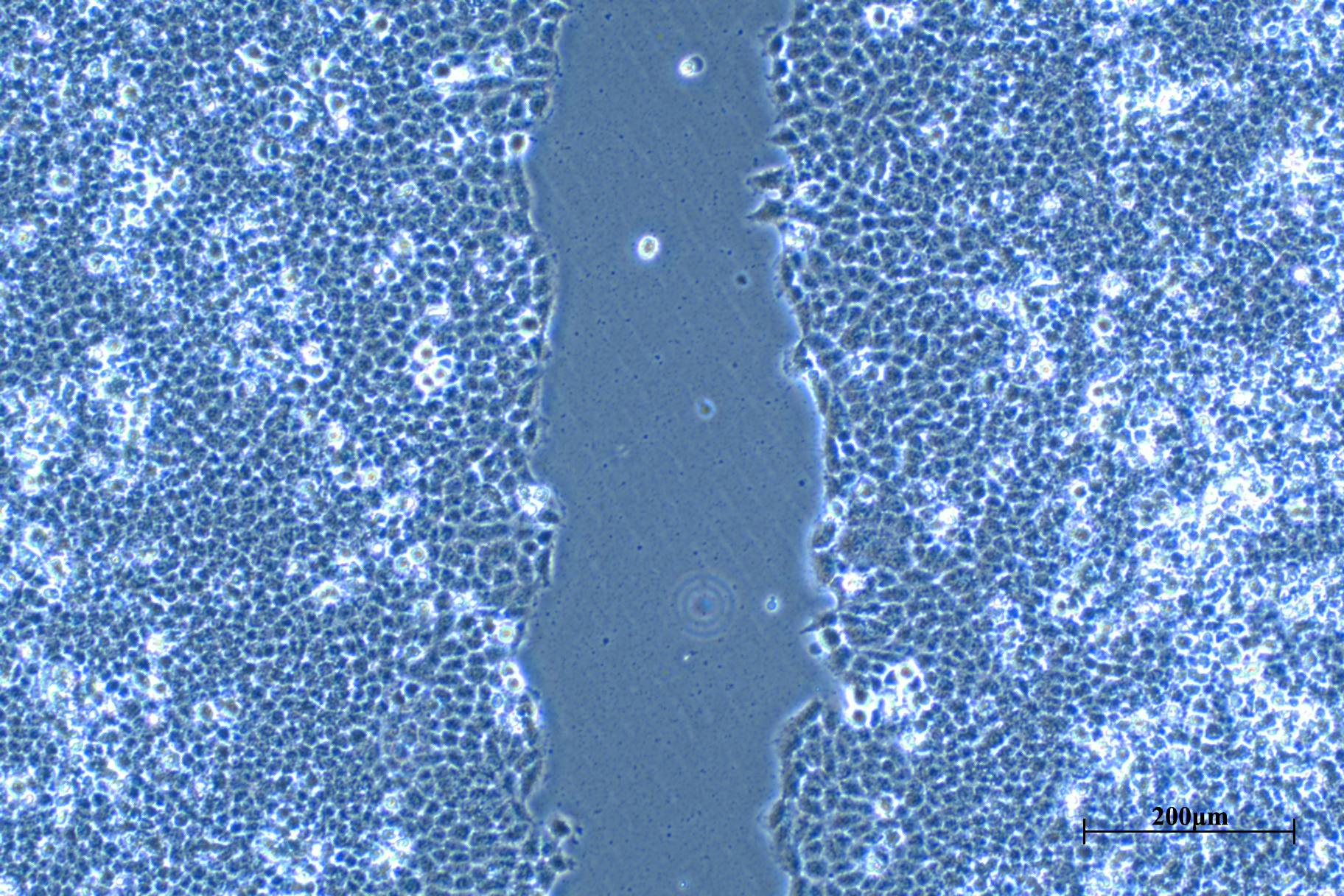

Supplement: Supplementary file 1 — Supplementary Information 1. [file 41598_2024_59725_MOESM1_ESM.zip › Original diagram of the cell experiment/fig3E/48 HOUR/4.tif]

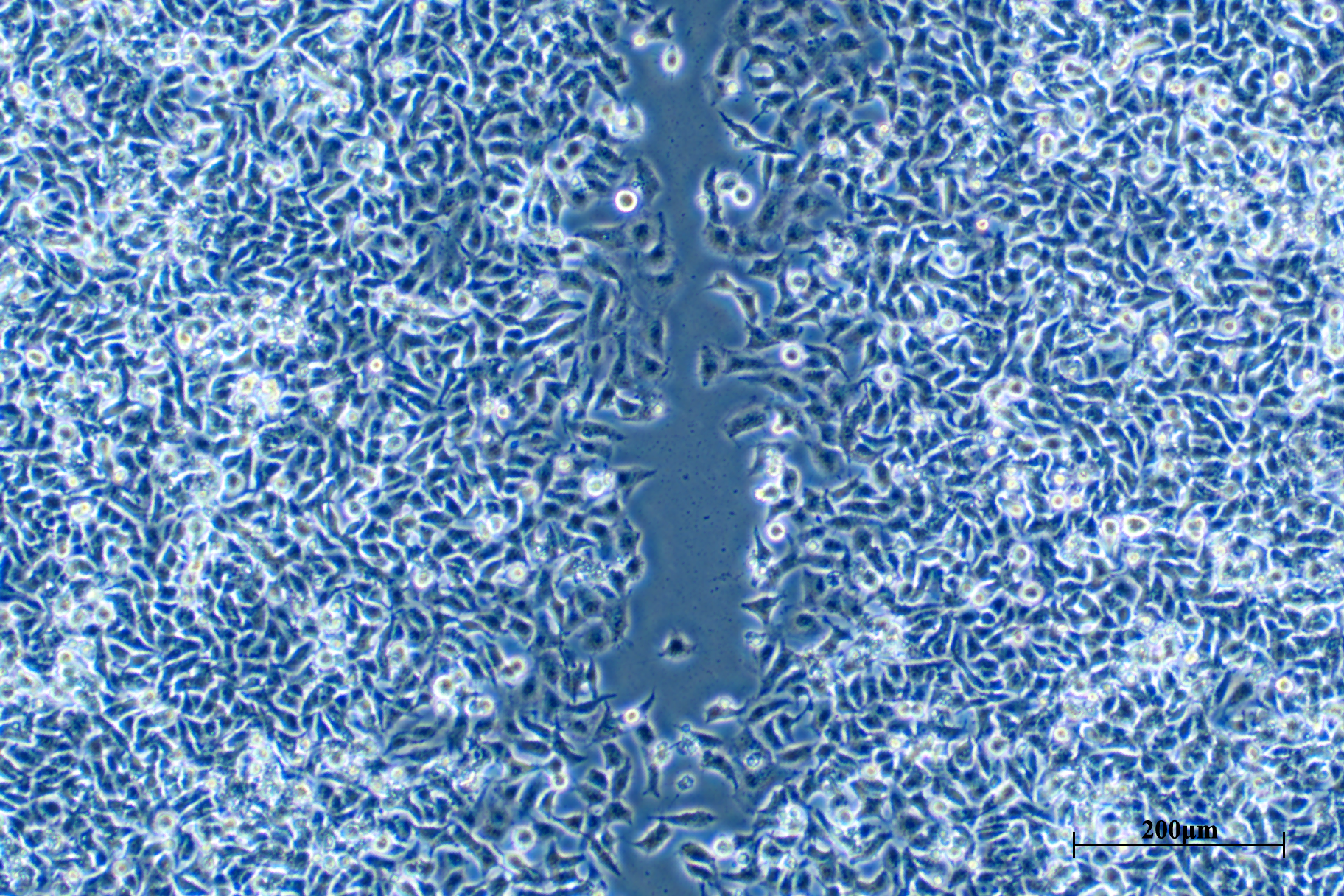

Supplement: Supplementary file 1 — Supplementary Information 1. [file 41598_2024_59725_MOESM1_ESM.zip › Original diagram of the cell experiment/fig3E/48 HOUR/5.tif]

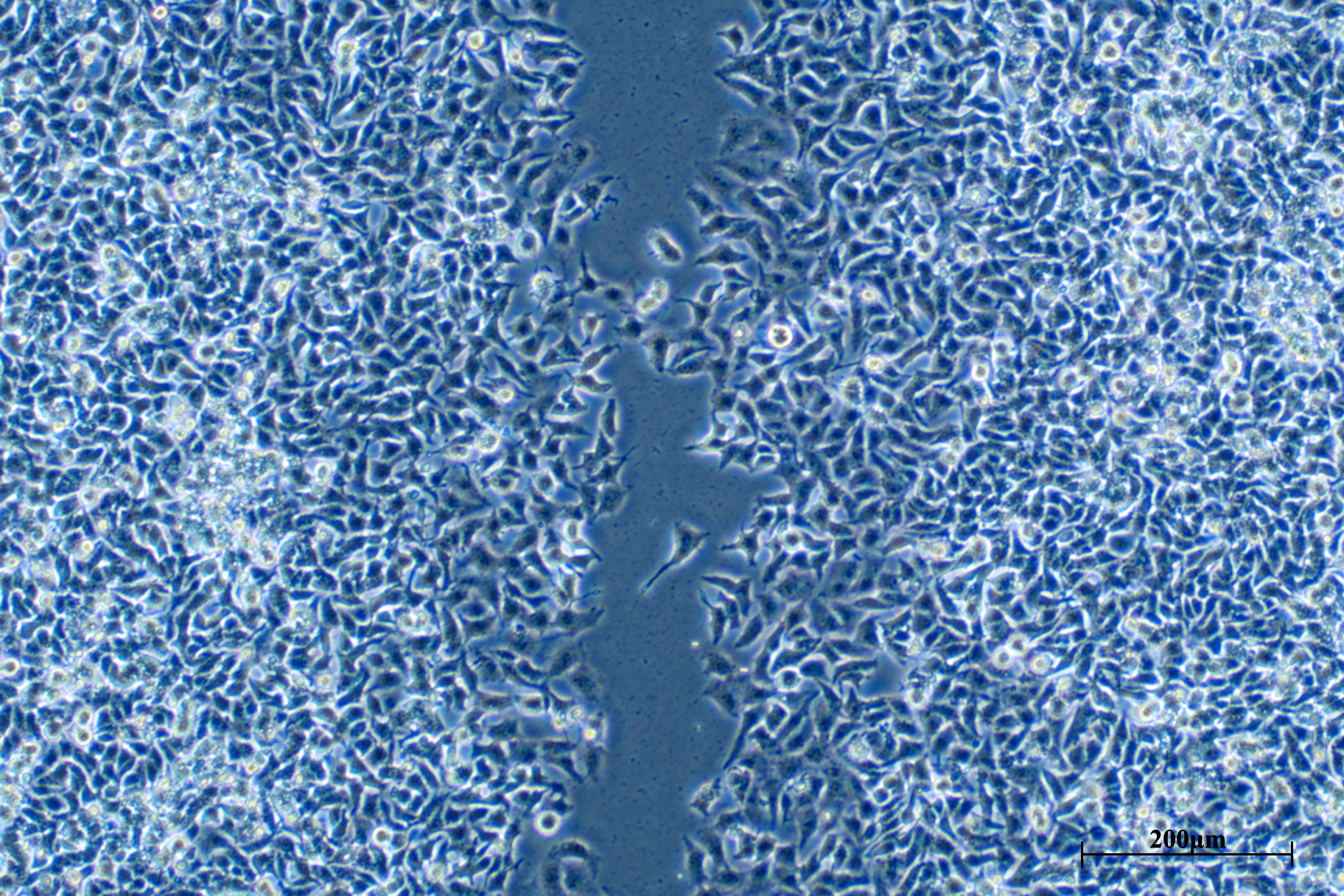

Supplement: Supplementary file 1 — Supplementary Information 1. [file 41598_2024_59725_MOESM1_ESM.zip › Original diagram of the cell experiment/fig3E/48 HOUR/6.tif]

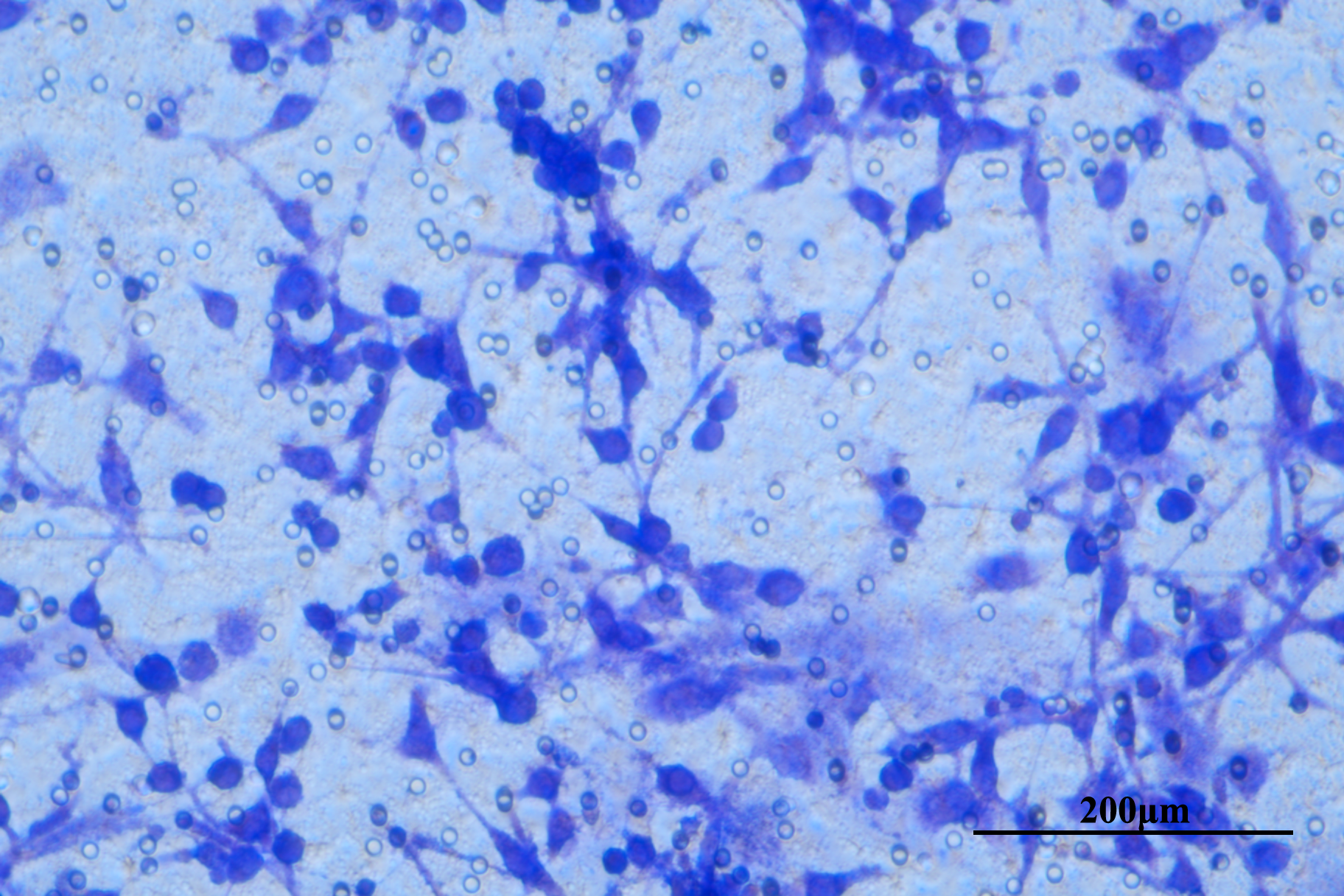

Supplement: Supplementary file 1 — Supplementary Information 1. [file 41598_2024_59725_MOESM1_ESM.zip › Original diagram of the cell experiment/fig3F/1.tif]

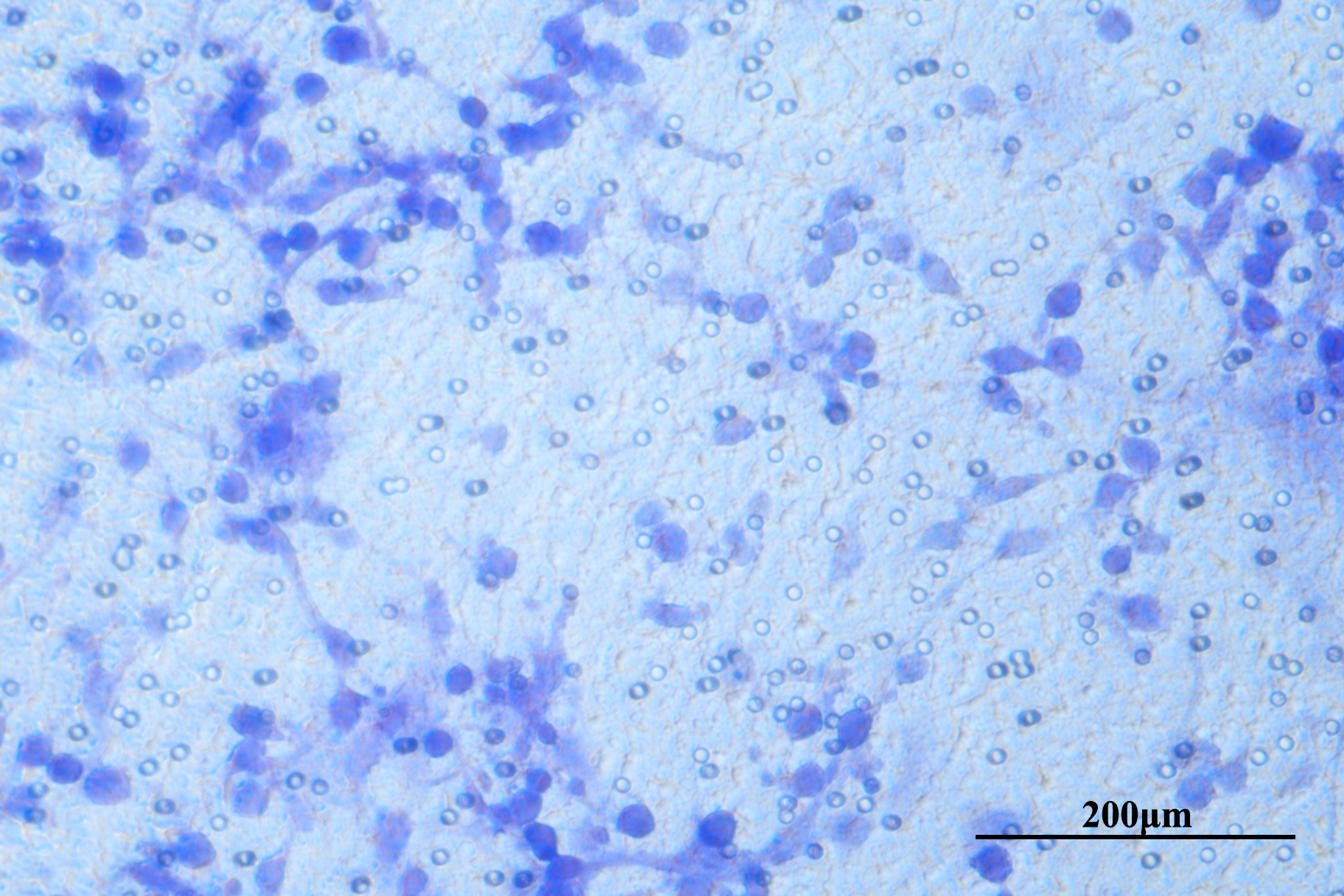

Supplement: Supplementary file 1 — Supplementary Information 1. [file 41598_2024_59725_MOESM1_ESM.zip › Original diagram of the cell experiment/fig3F/2.tif]

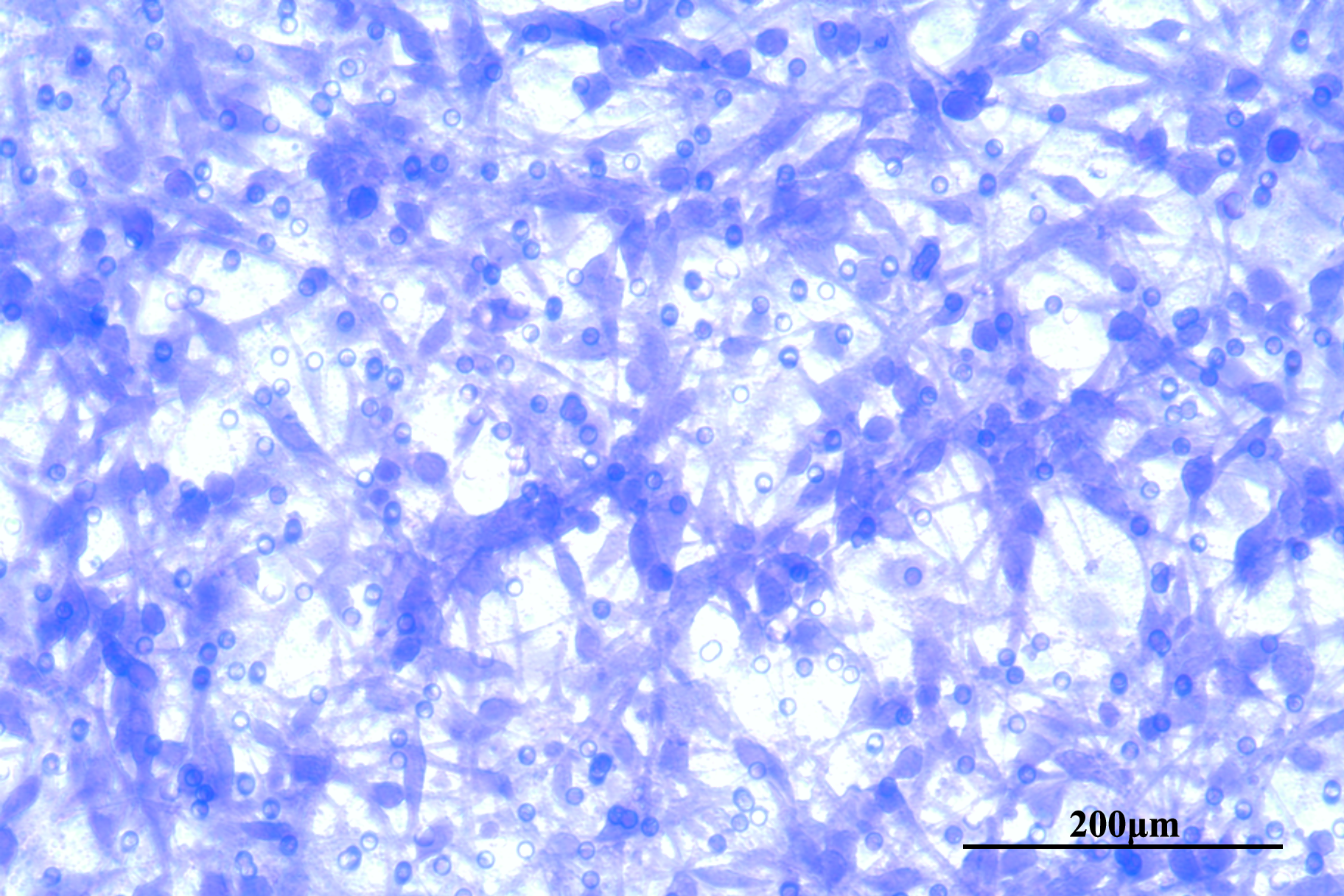

Supplement: Supplementary file 1 — Supplementary Information 1. [file 41598_2024_59725_MOESM1_ESM.zip › Original diagram of the cell experiment/fig3F/3.tif]

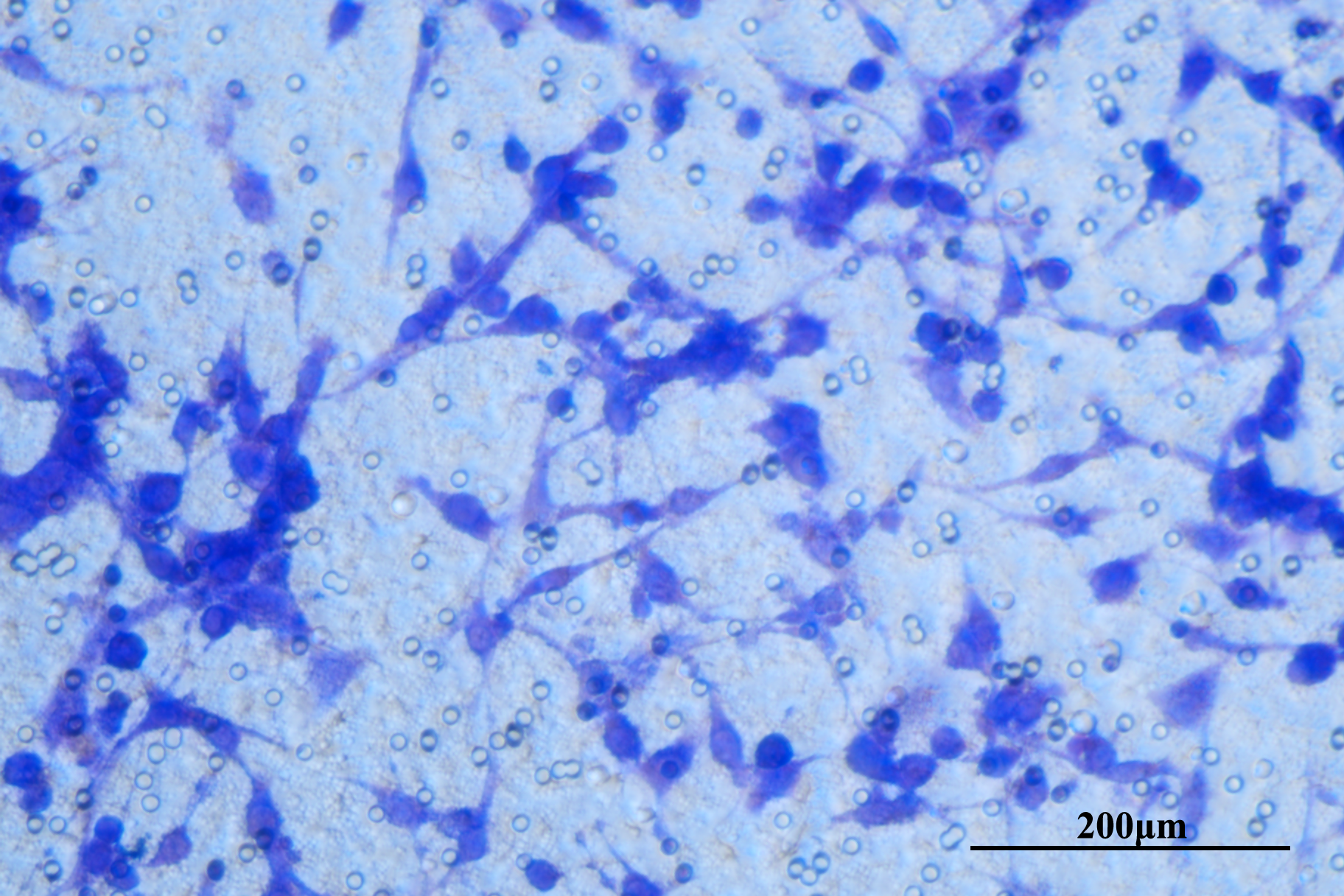

Supplement: Supplementary file 1 — Supplementary Information 1. [file 41598_2024_59725_MOESM1_ESM.zip › Original diagram of the cell experiment/fig3F/4.tif]

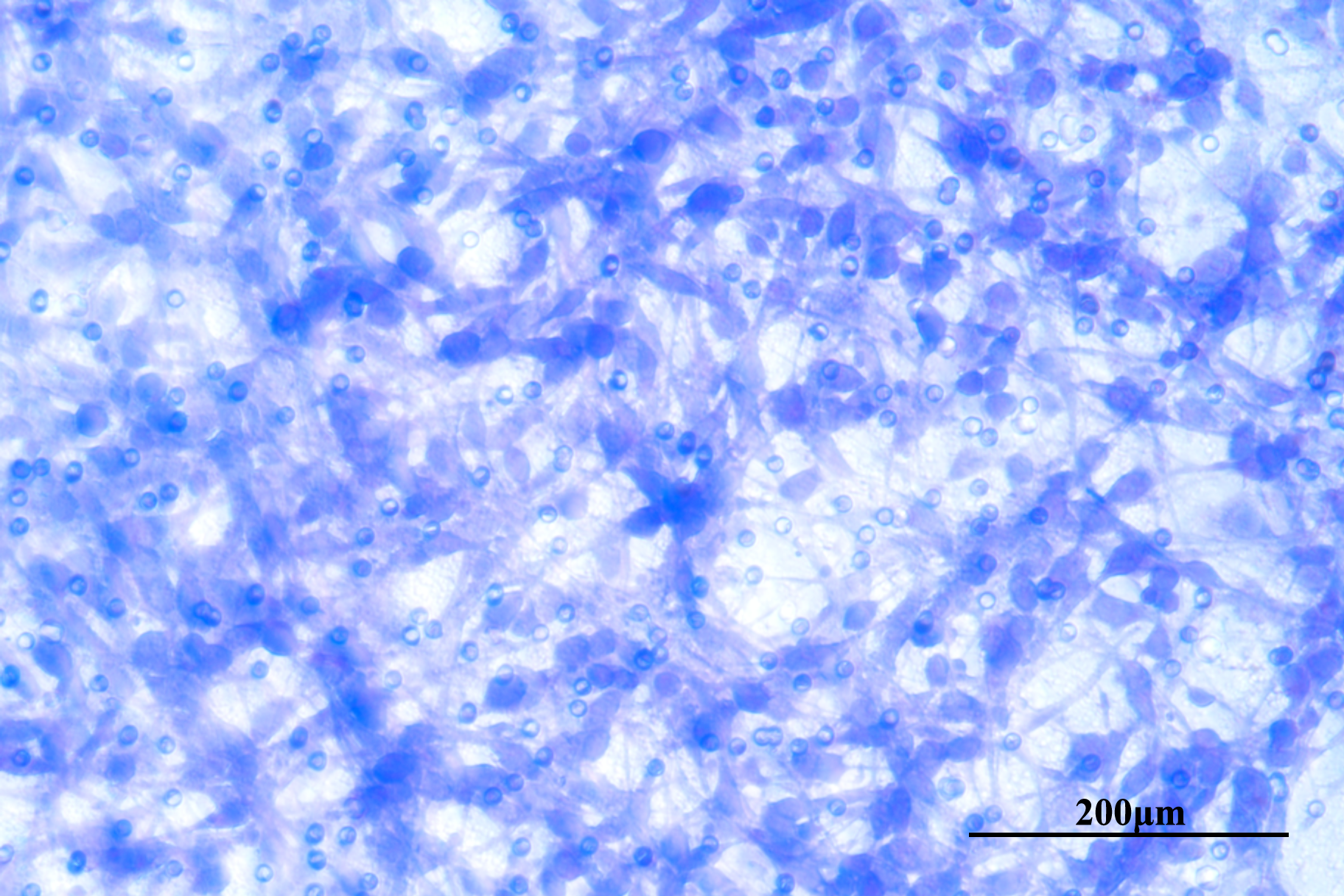

Supplement: Supplementary file 1 — Supplementary Information 1. [file 41598_2024_59725_MOESM1_ESM.zip › Original diagram of the cell experiment/fig3F/5.tif]

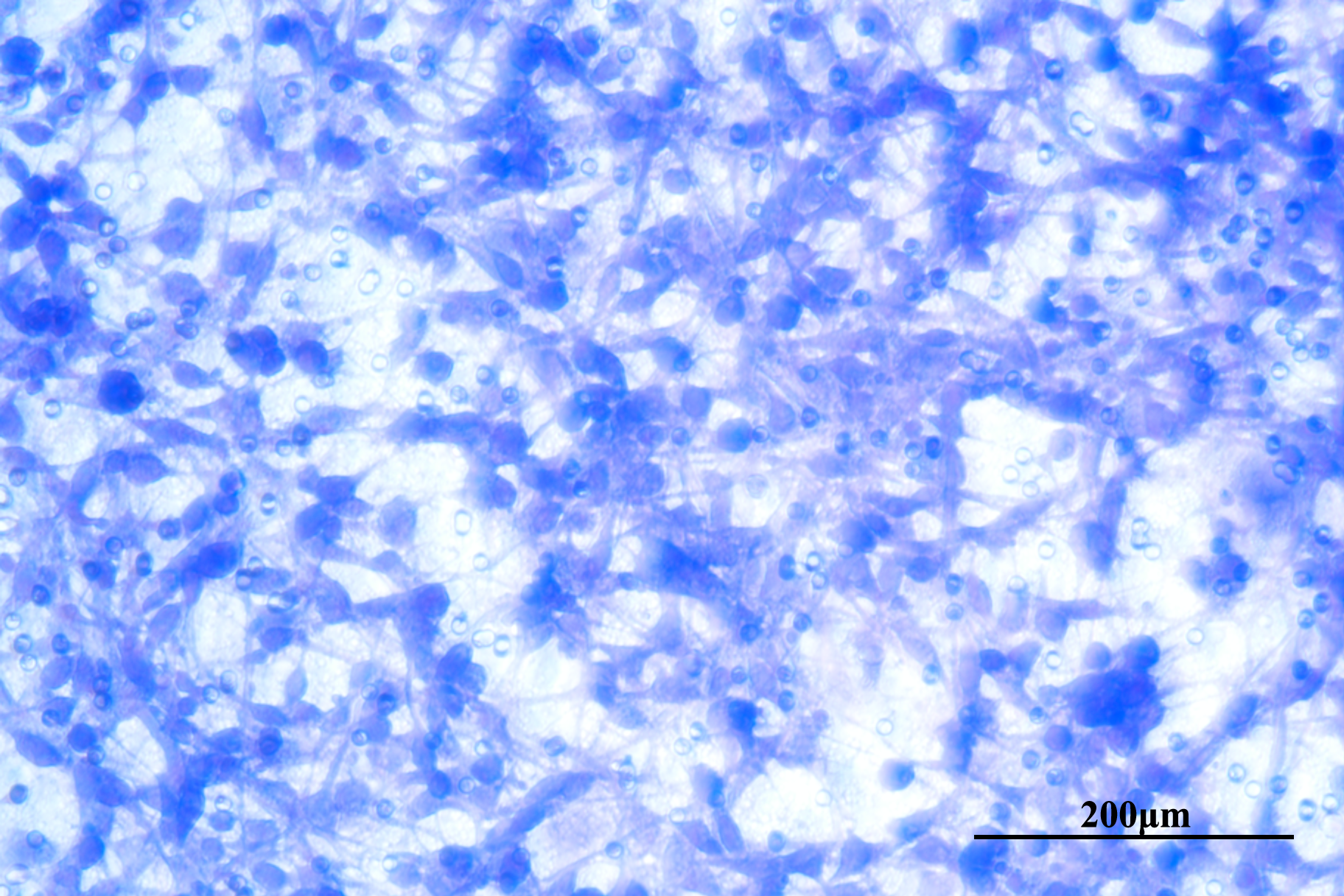

Supplement: Supplementary file 1 — Supplementary Information 1. [file 41598_2024_59725_MOESM1_ESM.zip › Original diagram of the cell experiment/fig3F/6.tif]

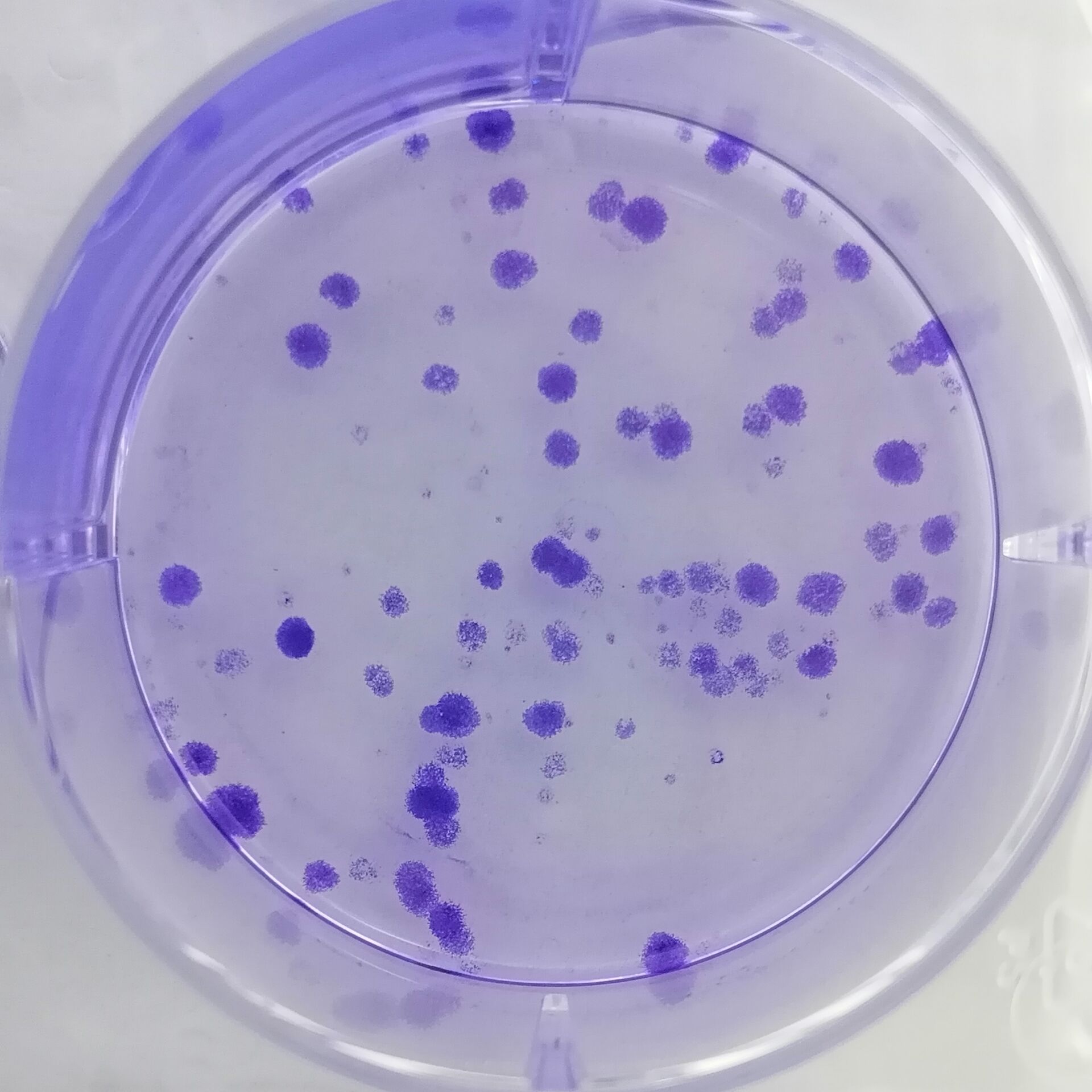

Supplement: Supplementary file 1 — Supplementary Information 1. [file 41598_2024_59725_MOESM1_ESM.zip › Original diagram of the cell experiment/fig4B/1.jpg]

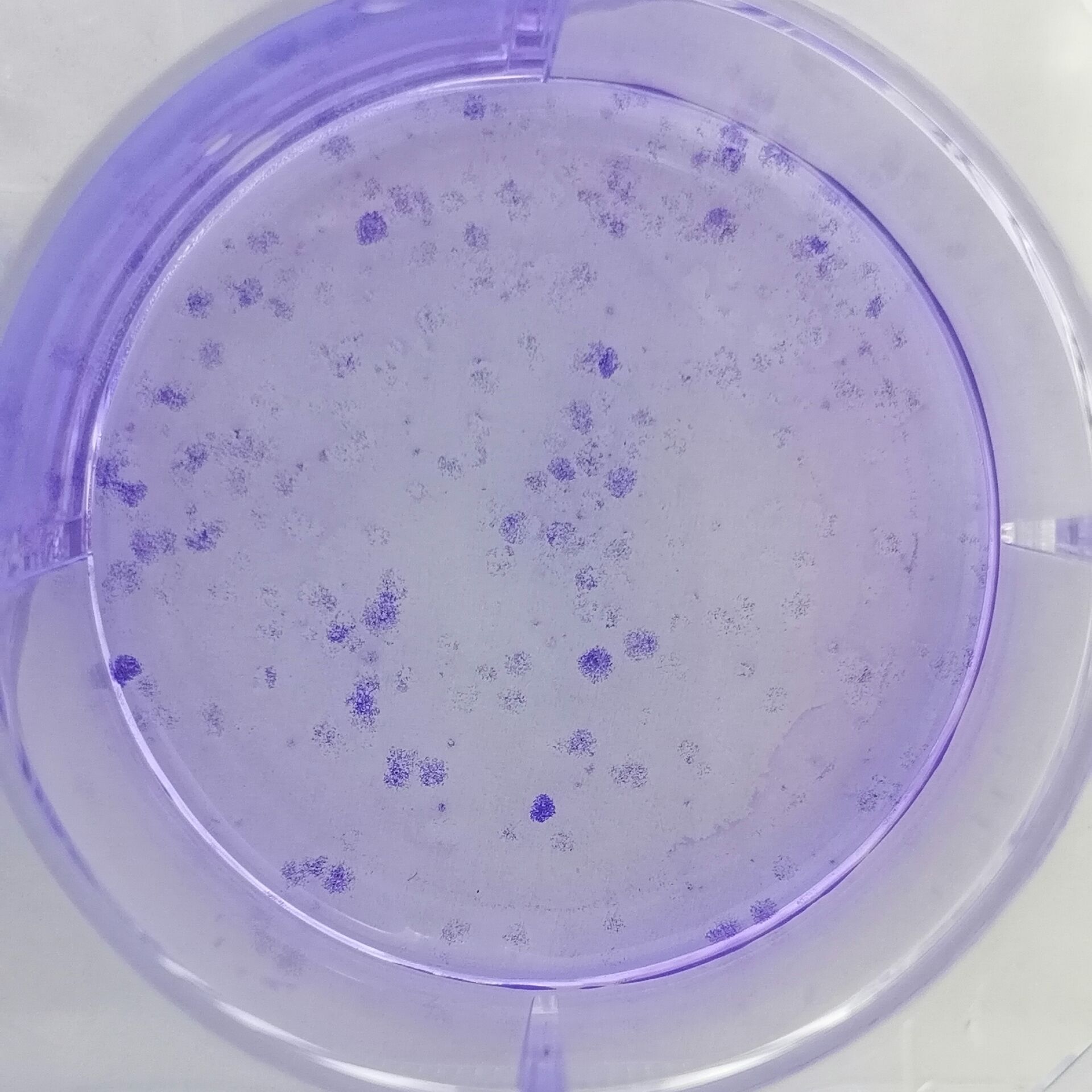

Supplement: Supplementary file 1 — Supplementary Information 1. [file 41598_2024_59725_MOESM1_ESM.zip › Original diagram of the cell experiment/fig4B/2.jpg]

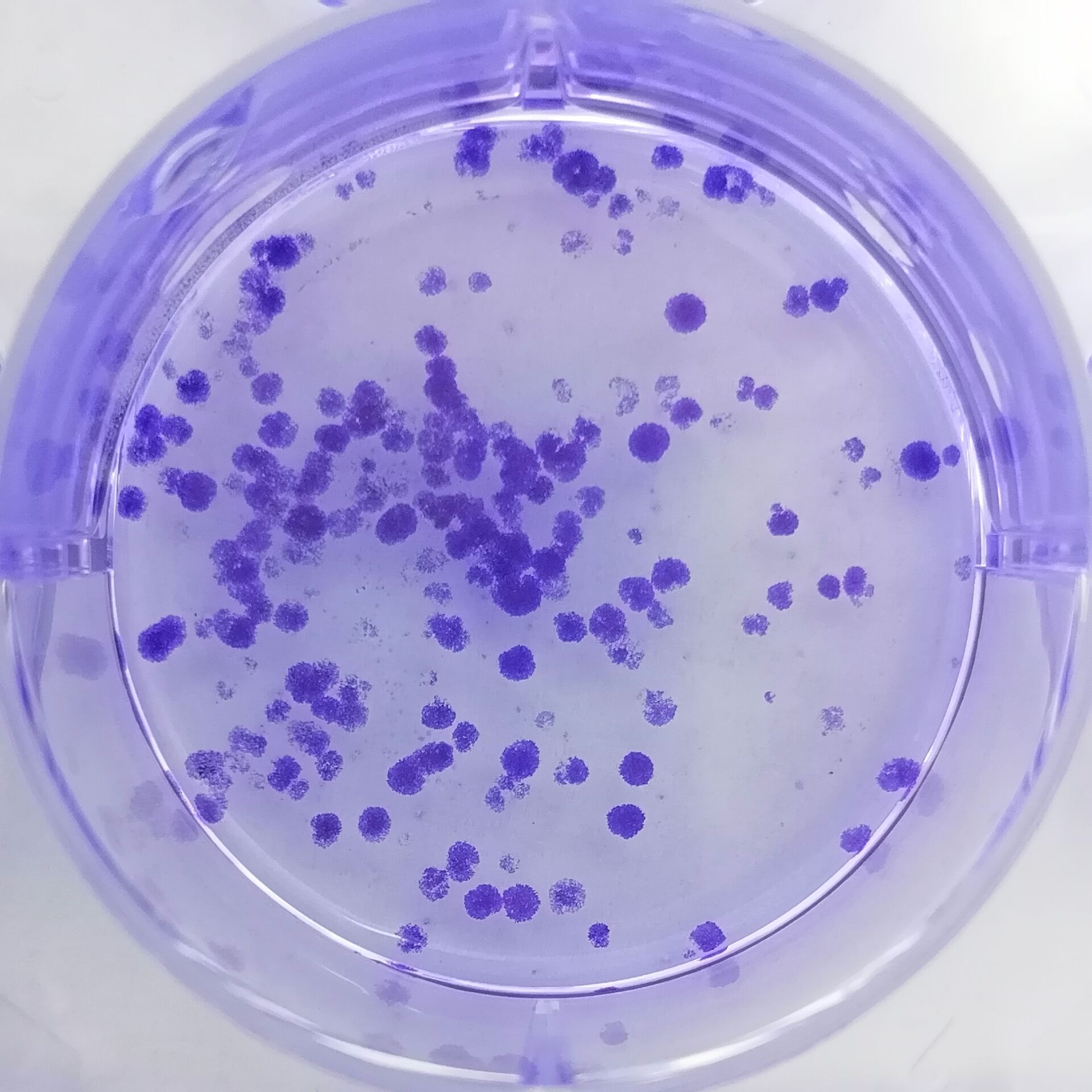

Supplement: Supplementary file 1 — Supplementary Information 1. [file 41598_2024_59725_MOESM1_ESM.zip › Original diagram of the cell experiment/fig4B/3.jpg]

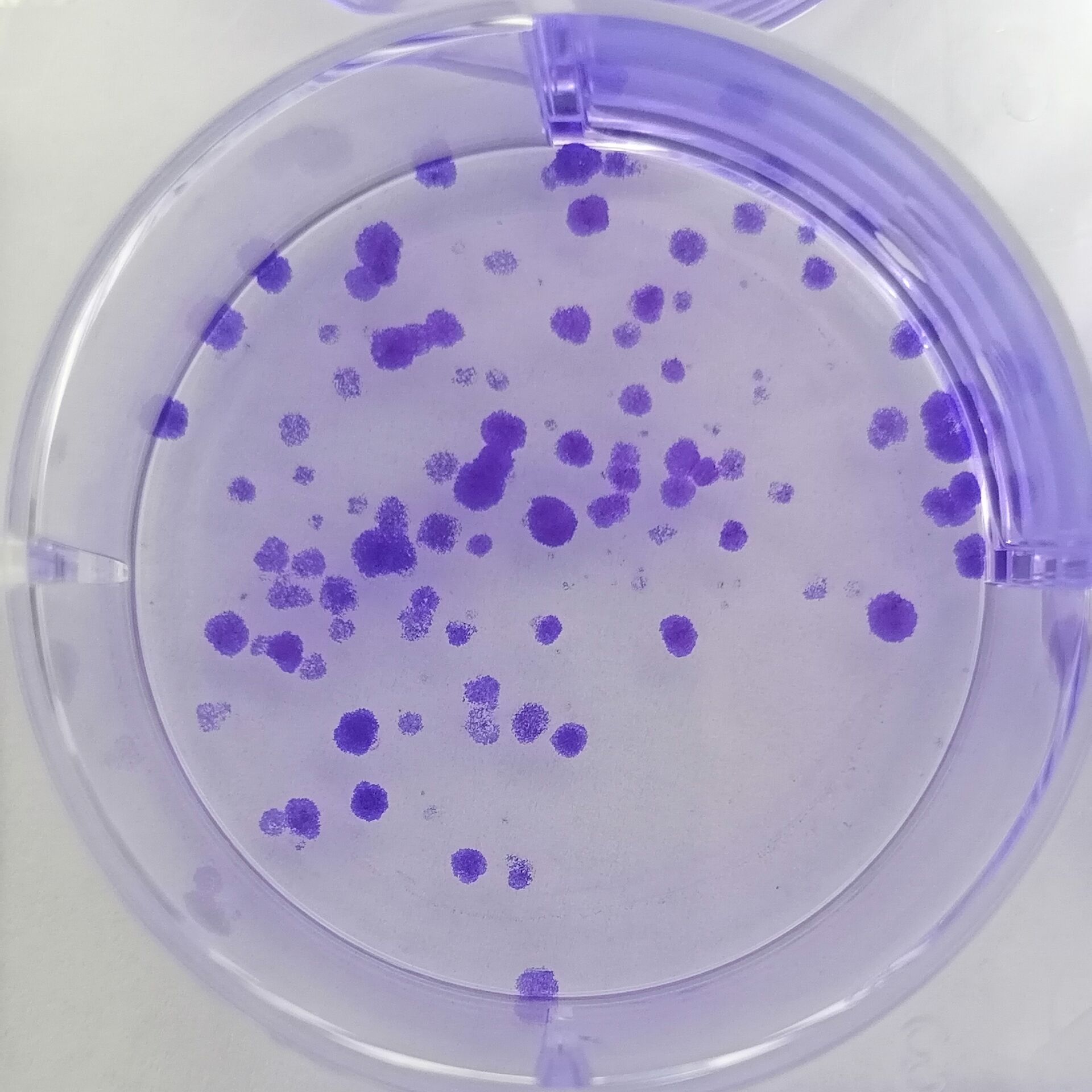

Supplement: Supplementary file 1 — Supplementary Information 1. [file 41598_2024_59725_MOESM1_ESM.zip › Original diagram of the cell experiment/fig4B/4.jpg]

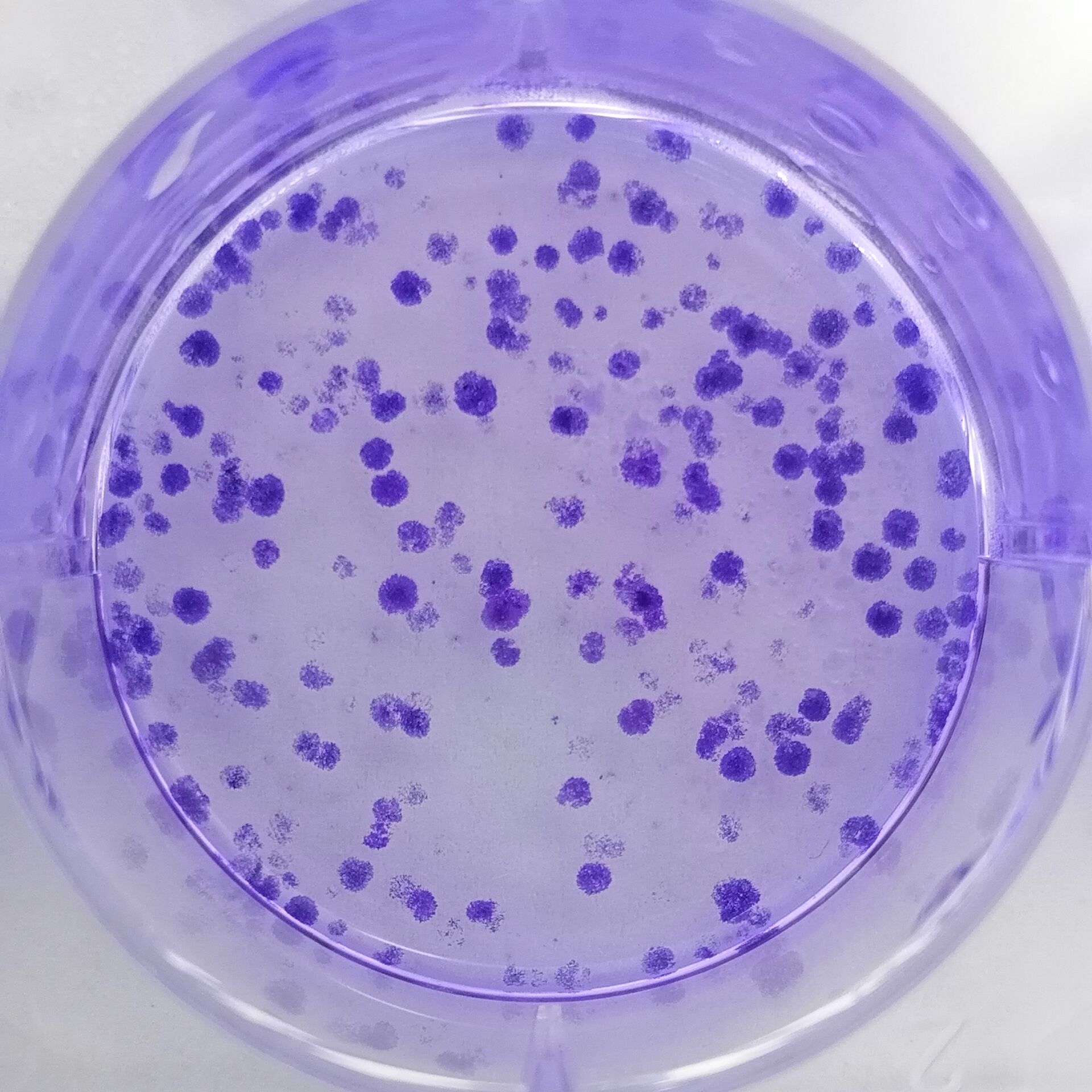

Supplement: Supplementary file 1 — Supplementary Information 1. [file 41598_2024_59725_MOESM1_ESM.zip › Original diagram of the cell experiment/fig4B/5.jpg]

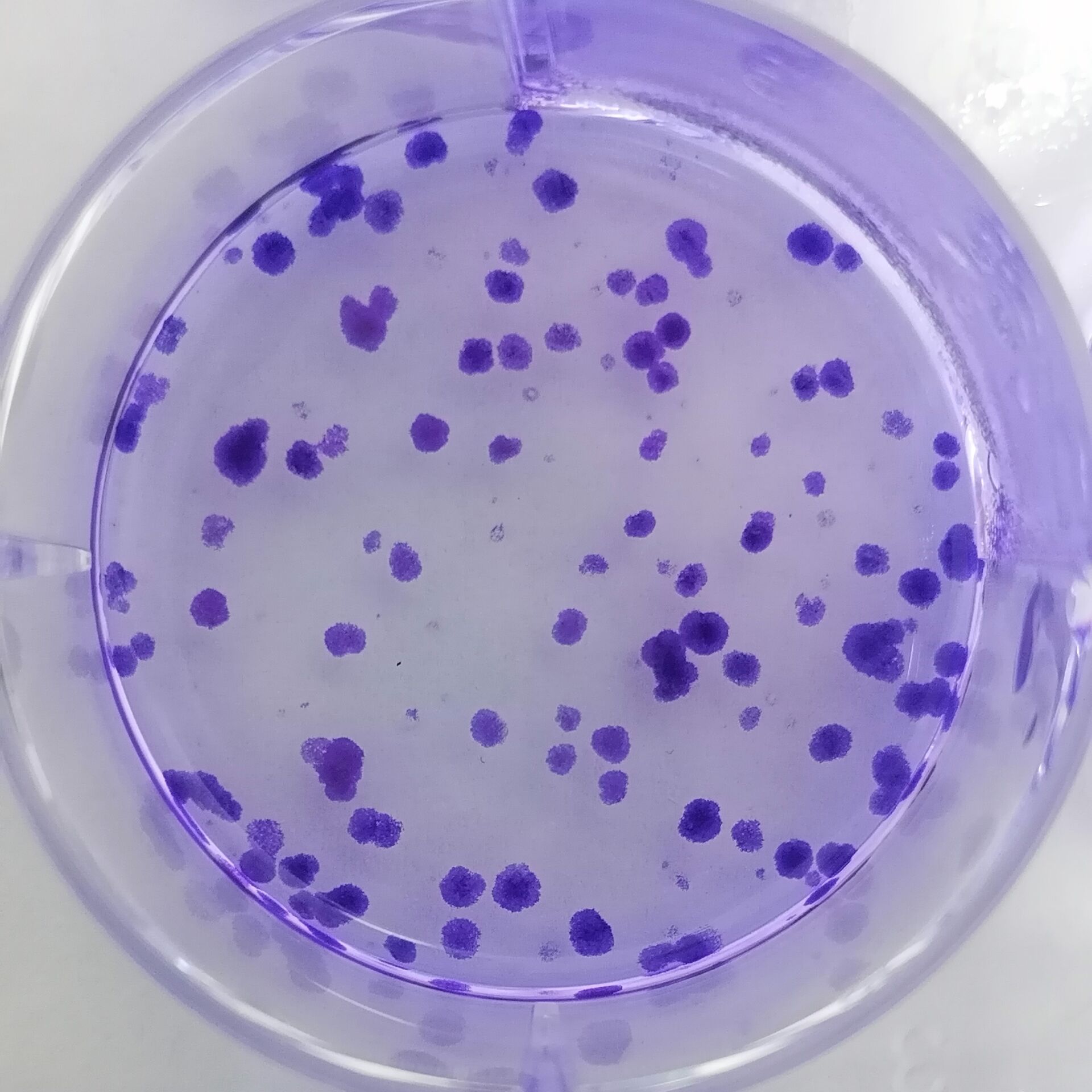

Supplement: Supplementary file 1 — Supplementary Information 1. [file 41598_2024_59725_MOESM1_ESM.zip › Original diagram of the cell experiment/fig4B/6.jpg]

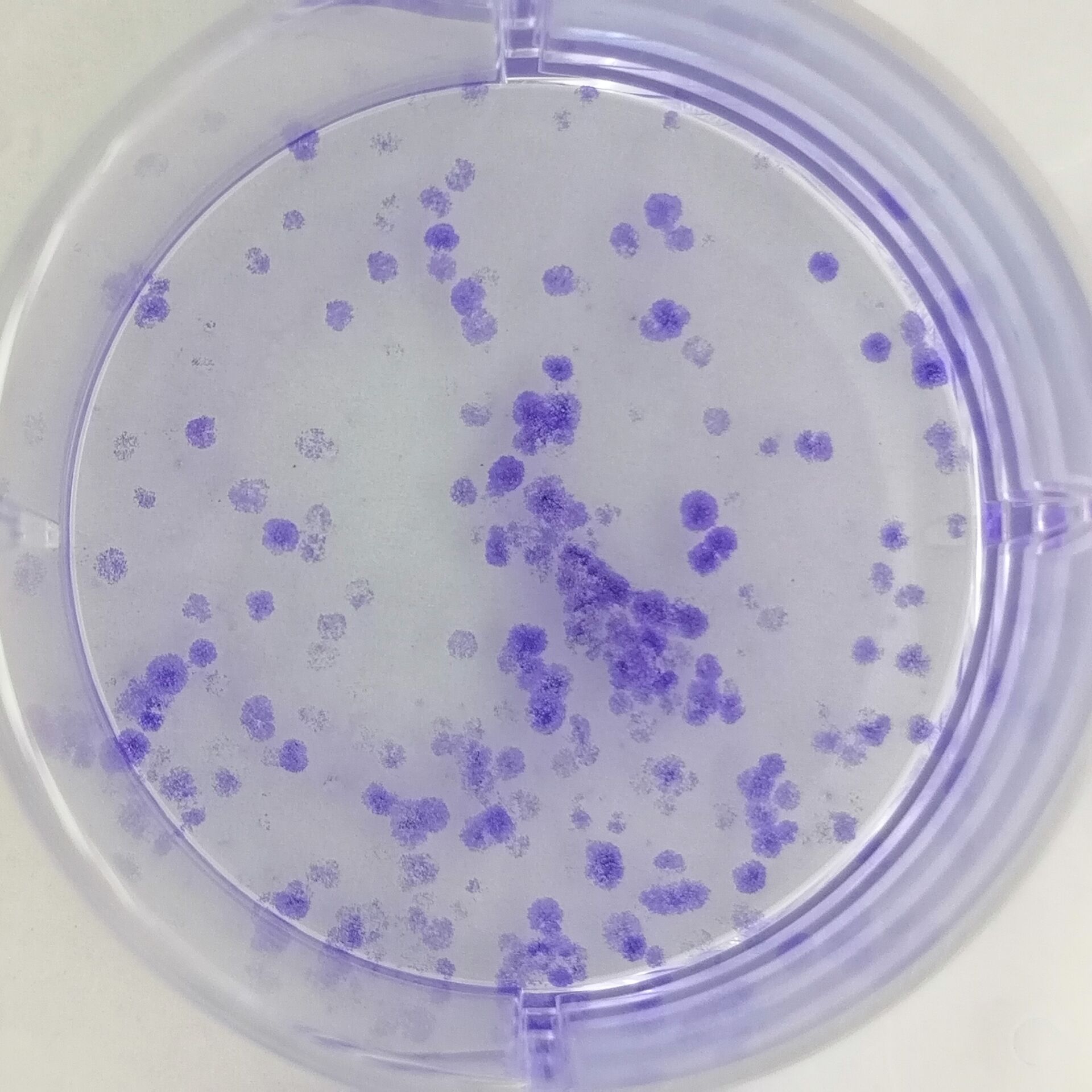

Supplement: Supplementary file 1 — Supplementary Information 1. [file 41598_2024_59725_MOESM1_ESM.zip › Original diagram of the cell experiment/fig4D/1.jpg]

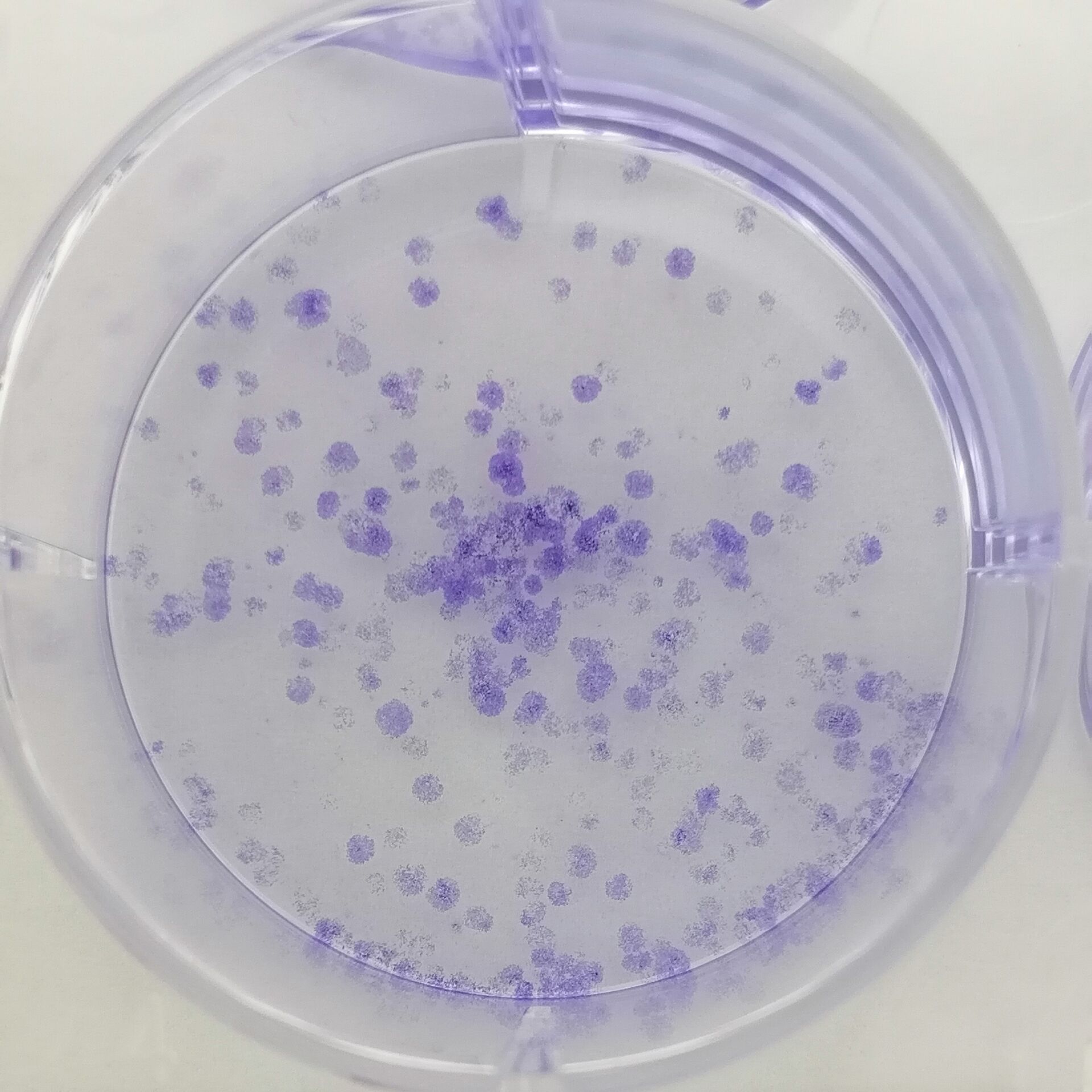

Supplement: Supplementary file 1 — Supplementary Information 1. [file 41598_2024_59725_MOESM1_ESM.zip › Original diagram of the cell experiment/fig4D/2.jpg]

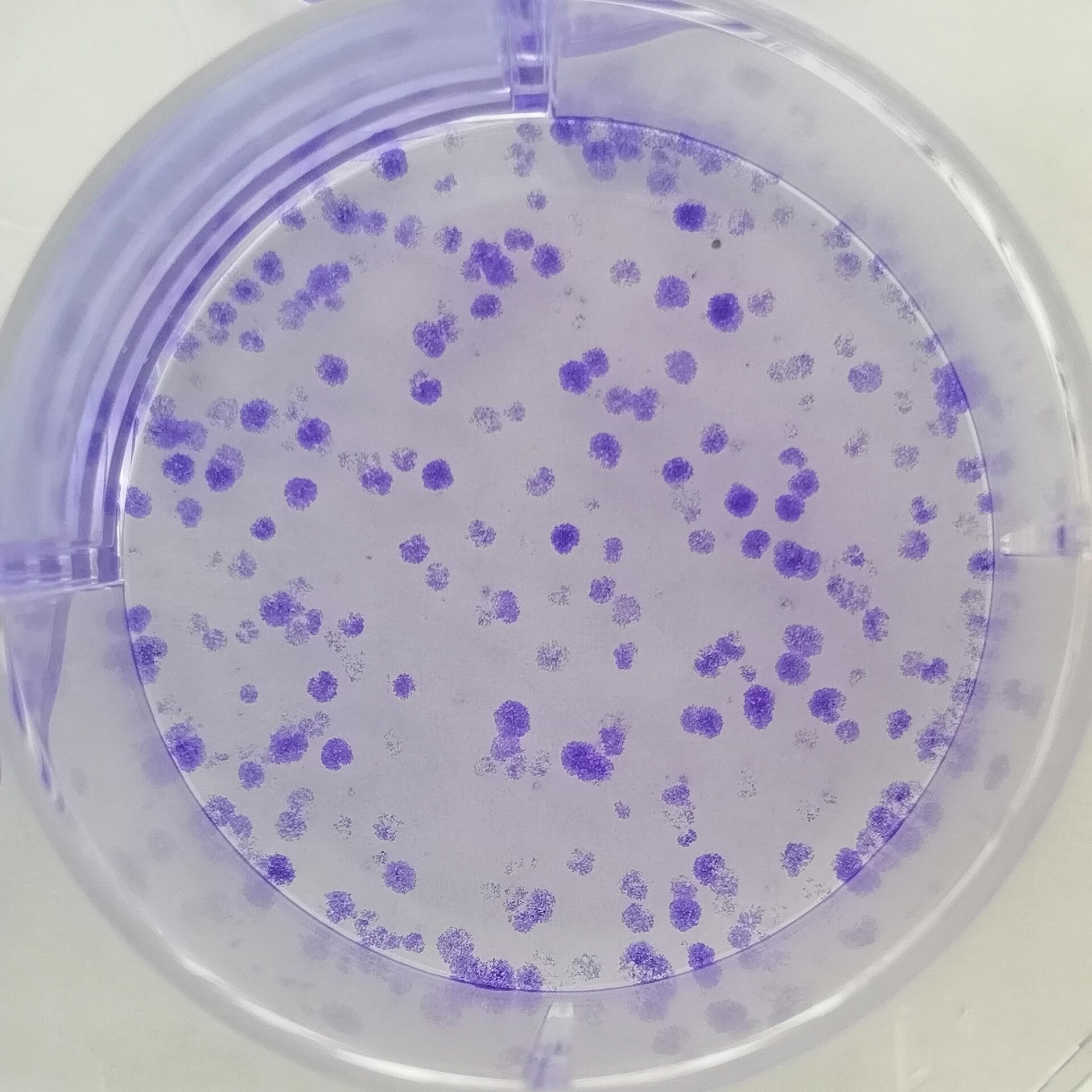

Supplement: Supplementary file 1 — Supplementary Information 1. [file 41598_2024_59725_MOESM1_ESM.zip › Original diagram of the cell experiment/fig4D/3.jpg]

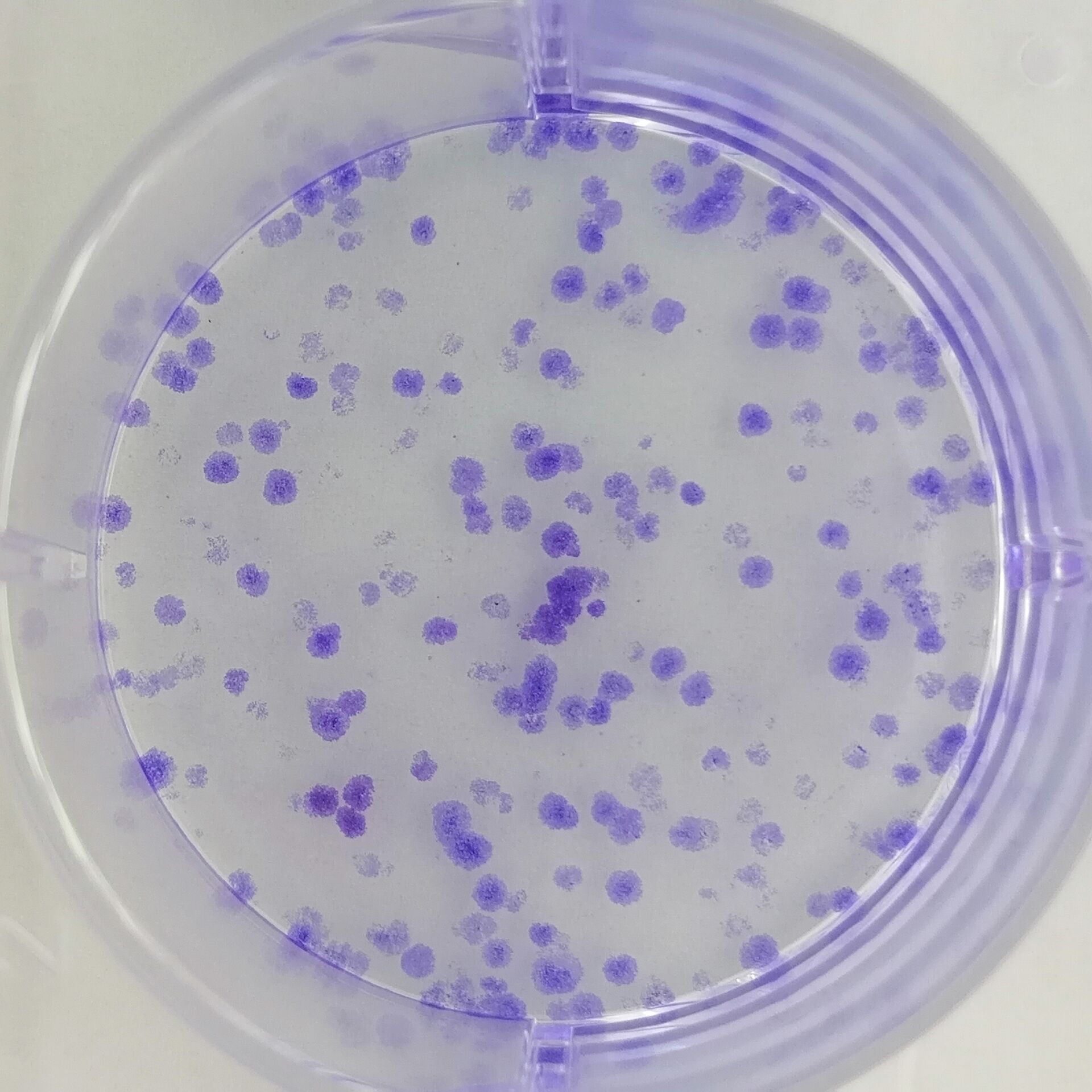

Supplement: Supplementary file 1 — Supplementary Information 1. [file 41598_2024_59725_MOESM1_ESM.zip › Original diagram of the cell experiment/fig4D/4.jpg]

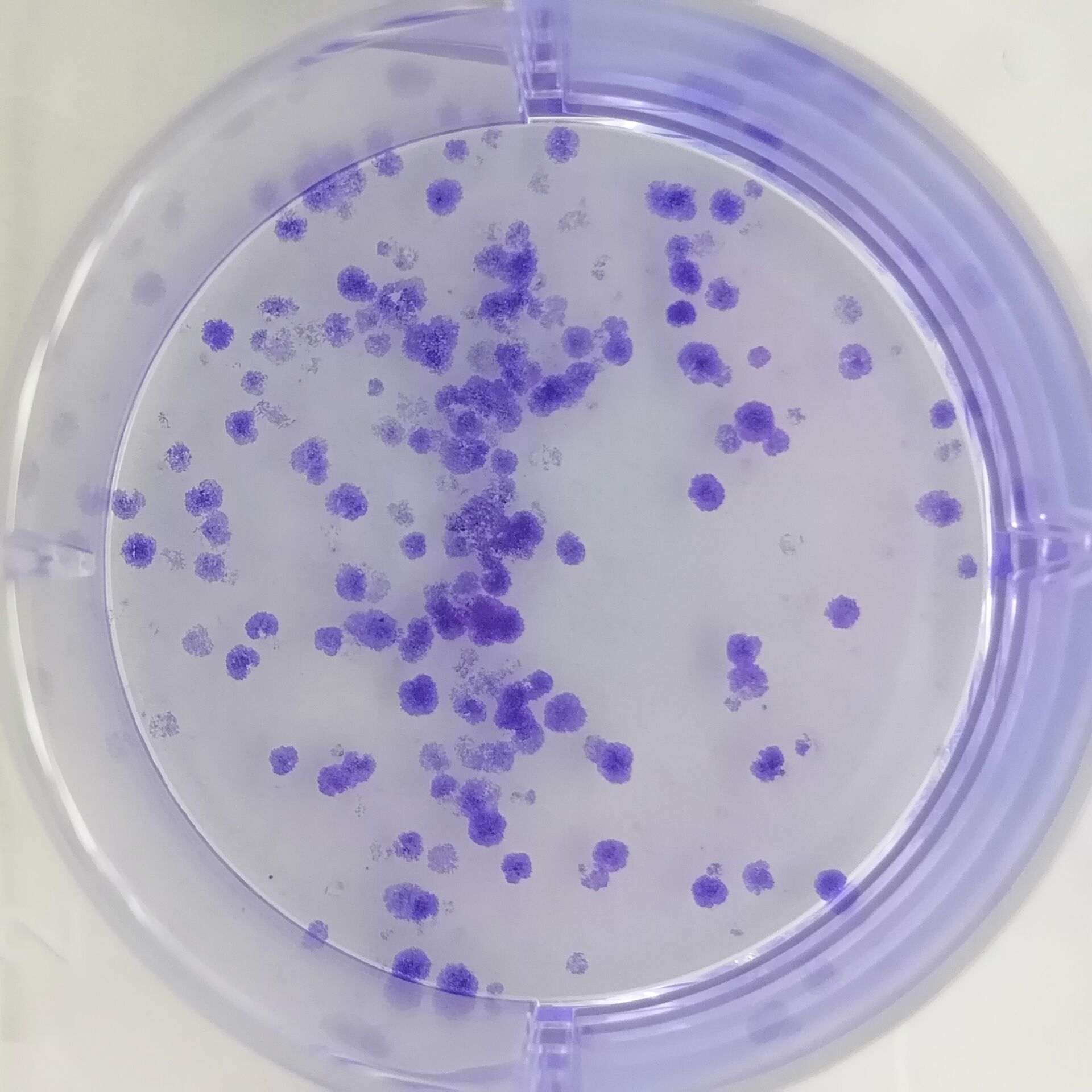

Supplement: Supplementary file 1 — Supplementary Information 1. [file 41598_2024_59725_MOESM1_ESM.zip › Original diagram of the cell experiment/fig4D/5.jpg]

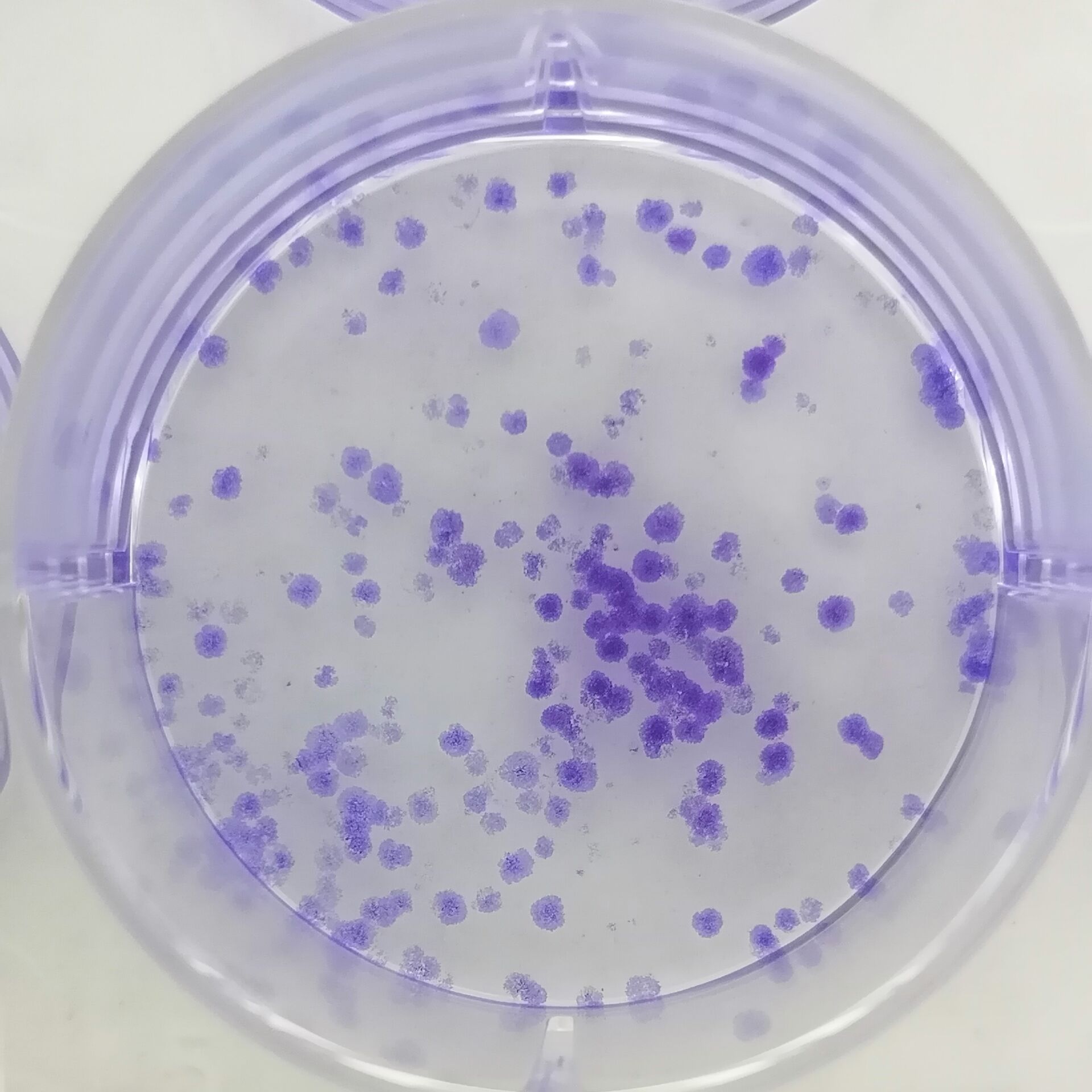

Supplement: Supplementary file 1 — Supplementary Information 1. [file 41598_2024_59725_MOESM1_ESM.zip › Original diagram of the cell experiment/fig4D/6.jpg]

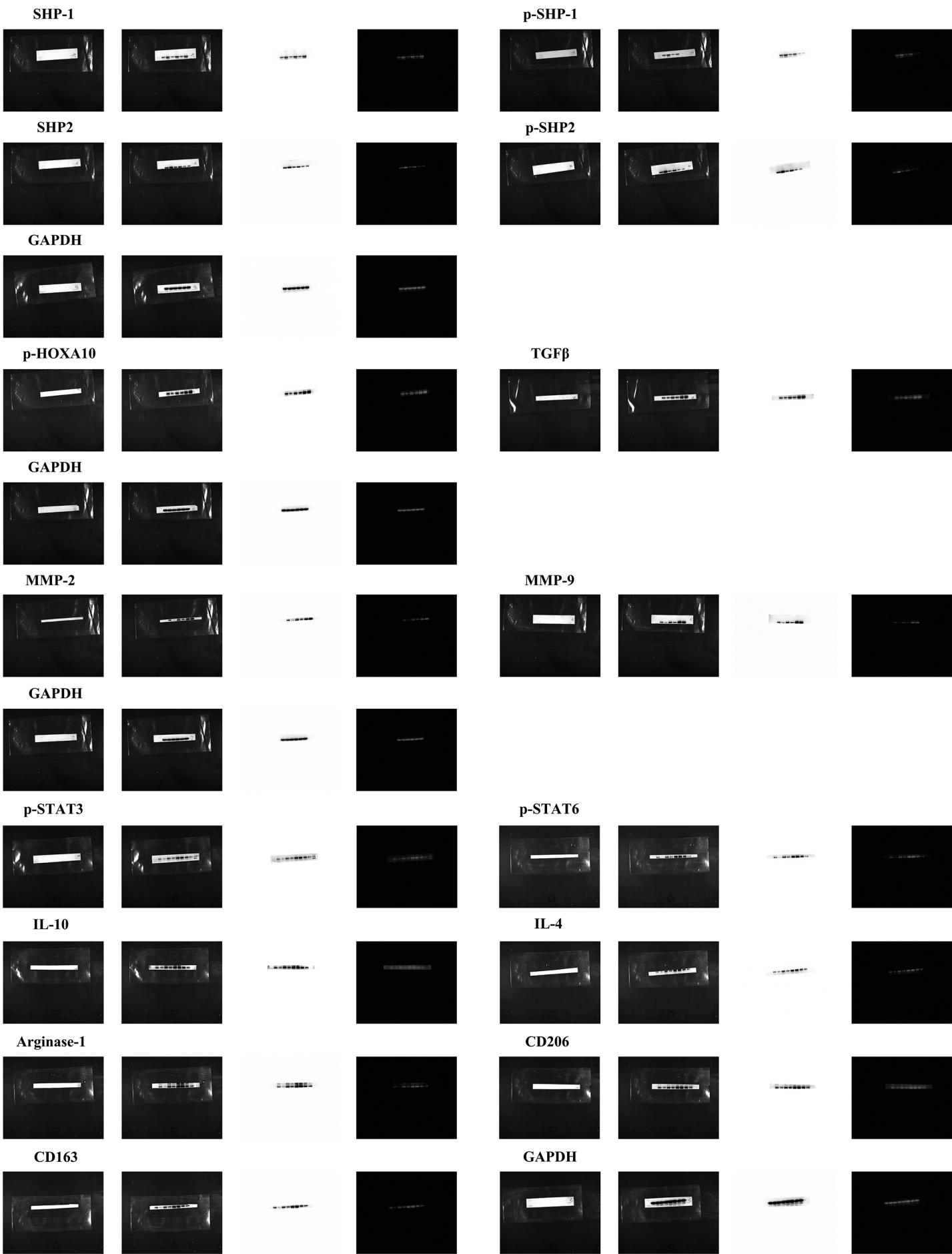

Supplement: Supplementary file 2 — Supplementary Information 2. [file 41598_2024_59725_MOESM2_ESM.pdf]
